# Prediction of individual lifetime cardiovascular risk and potential treatment benefit: development and recalibration of the LIFE-CVD2 model to four European risk regions

**DOI:** 10.1093/eurjpc/zwae174

**Published:** 2024-05-16

**Authors:** Steven H J Hageman, Stephen Kaptoge, Tamar I de Vries, Wentian Lu, Janet M Kist, Hendrikus J A van Os, Mattijs E Numans, Kristi Läll, Martin Bobak, Hynek Pikhart, Ruzena Kubinova, Sofia Malyutina, Andrzej Pająk, Abdonas Tamosiunas, Raimund Erbel, Andreas Stang, Börge Schmidt, Sara Schramm, Thomas R Bolton, Sarah Spackman, Stephan J L Bakker, Michael Blaha, Jolanda M A Boer, Amélie Bonnefond, Hermann Brenner, Eric J Brunner, Nancy R Cook, Karina Davidson, Elaine Dennison, Chiara Donfrancesco, Marcus Dörr, James S Floyd, Ian Ford, Michael Fu, Ron T Gansevoort, Simona Giampaoli, Richard F Gillum, Agustín Gómez-de-la-Cámara, Lise Lund Håheim, Per-Olof Hansson, Peter Harms, Steve E Humphries, M Kamran Ikram, J Wouter Jukema, Maryam Kavousi, Stefan Kiechl, Anna Kucharska-Newton, David Lora Pablos, Kunihiro Matsushita, Haakon E Meyer, Karel G M Moons, Martin Bødtker Mortensen, Mirthe Muilwijk, Børge G Nordestgaard, Chris Packard, Luigi Pamieri, Demosthenes Panagiotakos, Annette Peters, Louis Potier, Rui Providencia, Bruce M Psaty, Paul M Ridker, Beatriz Rodriguez, Annika Rosengren, Naveed Sattar, Ben Schöttker, Joseph E Schwartz, Steven Shea, Martin J Shipley, Reecha Sofat, Barbara Thorand, W M Monique Verschuren, Henry Völzke, Nicholas J Wareham, Leo Westbury, Peter Willeit, Bin Zhou, John Danesh, Frank L J Visseren, Emanuele Di Angelantonio, Lisa Pennells, Jannick A N Dorresteijn

**Affiliations:** Department of Vascular Medicine, University Medical Center Utrecht, Utrecht, The Netherlands; Department of Public Health and Primary Care, University of Cambridge, CambridgeUK; Department of Vascular Medicine, University Medical Center Utrecht, Utrecht, The Netherlands; Department of Epidemiology and Public Health, University College London, London, UK; Health Campus The Hague, Leiden University Medical Center, The Hague, the Netherlands; National eHealth Living Lab, Leiden University Medical Center, The Hague, the Netherlands; Health Campus The Hague, Leiden University Medical Center, The Hague, the Netherlands; Health Campus The Hague, Leiden University Medical Center, The Hague, the Netherlands; Estonian Genome Centre, Institute of Genomics, University of Tartu, Tartu, Estonia; Department of Epidemiology and Public Health, University College London, London, UK; RECETOX, Masaryk University, Brno, Czech Republic; Department of Epidemiology and Public Health, University College London, London, UK; RECETOX, Masaryk University, Brno, Czech Republic; National Institute of Public Health, Prague, Czech Republic; Independent Researcher; Department of Epidemiology and Population Studies, Institute of Public Health, Faculty of Health Sciences, Jagiellonian University Medical College, Kraków, Poland; Institute of Cardiology, Lithuanian University of Health Sciences, Kaunas, Lithuania; Institute for Medical Informatics, Biometry and Epidemiology, University Hospital Essen, University Duisburg-Essen, Essen, Germany; Institute for Medical Informatics, Biometry and Epidemiology, University Hospital Essen, University Duisburg-Essen, Essen, Germany; Institute for Medical Informatics, Biometry and Epidemiology, University Hospital Essen, University Duisburg-Essen, Essen, Germany; Institute for Medical Informatics, Biometry and Epidemiology, University Hospital Essen, University Duisburg-Essen, Essen, Germany; British Heart Foundation Data Science Centre, Health Data Research UK, London, UK; British Heart Foundation Cardiovascular Epidemiology Unit, Department of Public Health and Primary Care, University of Cambridge, Cambridge, UK; British Heart Foundation Cardiovascular Epidemiology Unit, Department of Public Health and Primary Care, University of Cambridge, Cambridge, UK; Victor Phillip Dahdaleh Heart and Lung Research Institute, University of Cambridge, CambridgeUK; Department of Internal Medicine, University Medical Centre Groningen, University of Groningen, Groningen, Netherlands; Johns Hopkins Ciccarone Center for the Prevention of Heart Disease, Johns Hopkins Hospital, Baltimore, MD, USA; Centre for Prevention, Lifestyle and Health, National Institute for Public Health and the Environment, Bilthoven, The Netherlands; Inserm/CNRS UMR 1283/8199, Pasteur Institute of Lille, EGID, Lille, France; University of Lille, Lille, France; Department of Metabolism, Digestion and Reproduction, Imperial College London, London, UK; Division of Clinical Epidemiology and Aging Research, German Cancer Research Center, Heidelberg, Germany; Network Aging Research, University of Heidelberg, Heidelberg, Germany; Department of Epidemiology and Public Health, University College London, London, UK; Brigham & Women’s Hospital, Harvard Medical School Harvard University, Boston, MA, USA; Feinstein Institutes for Medical Research, Northwell Health, New York, NY, USA; MRC Lifecourse Epidemiology Unit, University of Southampton, Southampton, UK; Department of Cardiovascular, Endocrine-Metabolic Diseases and Aging, Istituto Superiore di Sanita’, Rome, Italy; Institute for Community Medicine, University Medicine Greifswald, University of Greifswald, Greifswald, Germany; German Centre for Cardiovascular Disease (DZHK), Partner Site Greifswald; German Centre for Cardiovascular Disease (DZD), Site Greifswald, Greifswald, Germany; Cardiovascular Health Research Unit, Departments of Medicine and Epidemiology, University of Washington, Seattle, WA, USA; Robertson Center for Biostatistics, University of Glasgow, Glasgow, UK; Department of Medicine, Sahlgrenska University Hospital/Östra Hospital, Gothenburg, Sweden; Department of Internal Medicine, University Medical Centre Groningen, University of Groningen, Groningen, Netherlands; former Istituto Superiore di Sanità, Rome, Italy; Howard University Hospital, Washington, DC, USA; Instituto de Investigación Hospital 12 de Octubre, Madrid, Spain; Institute of Oral Biology, Faculty of Dentistry, University of Oslo, Oslo, Norway; Department of Molecular and Clinical Medicine, Institute of Medicine, University of Gothenburg, Sahlgrenska Academy, Gothenburg, Sweden; Department of General Practice, Amsterdam University Medical Center, Amsterdam, Netherlands; Institute of Cardiovascular Science, Faculty of Population Health Sciences, University College London, London, UK; Department of Epidemiology, Erasmus MC, University Medical Center Rotterdam, Rotterdam, Netherlands; Department of Cardiology, Leiden University Medical Center, The Netherlands; Netherlands Heart Institute, Leiden, the Netherlands; Department of Epidemiology, Erasmus MC, University Medical Center Rotterdam, Rotterdam, Netherlands; Department of Neurology, Innsbruck Medical University and VASCage, Research Centre on Vascular Ageing and Stroke, Innsbruck, Austria; College of Public Health, Department of Epidemiology, University of Kentucky, KY, USA; Instituto de Investigación Hospital 12 de Octubre, Universidad Complutense de Madrid (UCM), Madrid, Spain; Bloomberg School of Public Health, Johns Hopkins University, Baltimore, MD, USA; Norwegian Institute of Public Health, Oslo, Norway; Julius Center for Health Sciences and Primary Care, UMC Utrecht, Utrecht University, Utrecht, The Netherlands; Department of Clinical Biochemistry, Copenhagen University Hospital – Herlev Gentofte, Copenhagen, Denmark; Department of Clinical Medicine, Faculty of Health and Medical Sciences, University of Copenhagen, Copenhagen, Denmark; Department of Epidemiology and Data Science, Amsterdam University Medical Center, Amsterdam, Netherlands; Department of Clinical Biochemistry, Copenhagen University Hospital – Herlev Gentofte, Copenhagen, Denmark; Department of Clinical Medicine, Faculty of Health and Medical Sciences, University of Copenhagen, Copenhagen, Denmark; School of Cardiovascular & Metabolic Health, University of Glasgow, Glasgow, UK; Department of Cardiovascular, Dysmetabolic and Aging-associated Diseases, Istituto Superiore di Sanità, Rome, Italy; Harokopio University, Athens, Greece; Institute of Epidemiology, Helmholtz Zentrum München, German Research Center for Environmental Health, Neuherberg, Germany; IBE, Pettenkofer School of Public Health, Medical Faculty, Ludwig-Maximilians-Universität, Munich, Germany; German Centre for Cardiovascular Research (DZHK e.V.), partner site Munich Heart Alliance, Munich, Germany; Université Paris City, Paris, France; Department of Diabetology, Endocrinology and Nutrition, Assistance Publique - Hôpitaux de Paris, Bichat Hospital, Paris, France; Institute of Health Informatics Research, University College London, London, UK; Cardiovascular Health Research Unit, University of Washington, Seattle, WA, USA; Brigham & Women’s Hospital, Harvard Medical School Harvard University, Boston, MA, USA; University of Hawaii and Tecnologico de Monterrey, Honolulu, HI, USA; Sahlgrenska University Hospital and Östra Hospital, Göteborg, Sweden; School of Cardiovascular and Metabolic Health, University of Glasgow, Glasgow, UK; Division of Clinical Epidemiology and Aging Research, German Cancer Research Center, Heidelberg, Germany; Network Aging Research, University of Heidelberg, Heidelberg, Germany; Columbia University, New York, NY, USA; College of Physicians & Surgeons and Mailman School of Public Health, Columbia University, NY, USA; Department of Epidemiology and Public Health, University College London, London, UK; Department of Pharmacology and Therapeutics, University of Liverpool, Liverpool, UK; Institute of Epidemiology, Helmholtz Zentrum München, German Research Center for Environmental Health, Neuherberg, Germany; IBE, Pettenkofer School of Public Health, Medical Faculty, Ludwig-Maximilians-Universität, Munich, Germany; Centre for Prevention, Lifestyle and Health, National Institute for Public Health and the Environment, Bilthoven, The Netherlands; Julius Center for Health Sciences and Primary Care, UMC Utrecht, Utrecht University, Utrecht, The Netherlands; Institute for Community Medicine, University Medicine Greifswald, University of Greifswald, Greifswald, Germany; German Centre for Cardiovascular Disease (DZHK), Partner Site Greifswald; German Centre for Cardiovascular Disease (DZD), Site Greifswald, Greifswald, Germany; MRC Epidemiology Unit, University of Cambridge School of Clinical Medicine, University of Cambridge, Cambridge, UK; MRC Lifecourse Epidemiology Unit, University of Southampton, Southampton, UK; British Heart Foundation Cardiovascular Epidemiology Unit, Department of Public Health and Primary Care, University of Cambridge, Cambridge, UK; Department of Medical Statistics, Informatics and Health Economics, Medical University of Innsbruck, Innsbruck, Austria; Faculty of Medicine, School of Public Health, Imperial College London, London, UK; British Heart Foundation Cardiovascular Epidemiology Unit, Department of Public Health and Primary Care, University of Cambridge, Cambridge, UK; Victor Phillip Dahdaleh Heart and Lung Research Institute, University of Cambridge, CambridgeUK; Department of Vascular Medicine, University Medical Center Utrecht, Utrecht, The Netherlands; Department of Public Health and Primary Care, University of Cambridge, CambridgeUK; Department of Public Health and Primary Care, University of Cambridge, CambridgeUK; Department of Vascular Medicine, University Medical Center Utrecht, Utrecht, The Netherlands

**Keywords:** Risk prediction, Lifetime, Prevention, Cardiovascular disease, Primary prevention

## Abstract

**Aims:**

The 2021 European Society of Cardiology prevention guidelines recommend the use of (lifetime) risk prediction models to aid decisions regarding initiation of prevention. We aimed to update and systematically recalibrate the LIFEtime-perspective CardioVascular Disease (LIFE-CVD) model to four European risk regions for the estimation of lifetime CVD risk for apparently healthy individuals.

**Methods and results:**

The updated LIFE-CVD (i.e. LIFE-CVD2) models were derived using individual participant data from 44 cohorts in 13 countries (687 135 individuals without established CVD, 30 939 CVD events in median 10.7 years of follow-up). LIFE-CVD2 uses sex-specific functions to estimate the lifetime risk of fatal and non-fatal CVD events with adjustment for the competing risk of non-CVD death and is systematically recalibrated to four distinct European risk regions. The updated models showed good discrimination in external validation among 1 657 707 individuals (61 311 CVD events) from eight additional European cohorts in seven countries, with a pooled C-index of 0.795 (95% confidence interval 0.767–0.822). Predicted and observed CVD event risks were well calibrated in population-wide electronic health records data in the UK (Clinical Practice Research Datalink) and the Netherlands (Extramural LUMC Academic Network). When using LIFE-CVD2 to estimate potential gain in CVD-free life expectancy from preventive therapy, projections varied by risk region reflecting important regional differences in absolute lifetime risk. For example, a 50-year-old smoking woman with a systolic blood pressure (SBP) of 140 mmHg was estimated to gain 0.9 years in the low-risk region vs. 1.6 years in the very high-risk region from lifelong 10 mmHg SBP reduction. The benefit of smoking cessation for this individual ranged from 3.6 years in the low-risk region to 4.8 years in the very high-risk region.

**Conclusion:**

By taking into account geographical differences in CVD incidence using contemporary representative data sources, the recalibrated LIFE-CVD2 model provides a more accurate tool for the prediction of lifetime risk and CVD-free life expectancy for individuals without previous CVD, facilitating shared decision-making for cardiovascular prevention as recommended by 2021 European guidelines.

**See the editorial comment for this article ‘The complexities of modelling lifetime risk in the general population’, by M. Bahls and S. Groß, https://doi.org/10.1093/eurjpc/zwae152.**

## Introduction

Cardiovascular diseases (CVDs), which include coronary heart disease and stroke, are the most common fatal non-communicable diseases globally, responsible for an estimated 18.6 million deaths in 2019.^1^ Cardiovascular disease remains a major cause of morbidity and mortality in Europe. A key strategy in the prevention of CVD is the use of risk prediction algorithms to target preventive interventions on people who benefit from them most.^2,3^ Recent European Society of Cardiology (ESC) CVD prevention guidelines recommend an individualized strategy regarding initiation of preventive treatment, in which personal preferences, expected treatment side effects, predicted 10-year CVD risk, and/or lifetime risk estimates are all taken into account in a shared decision-making process.^4^ Since age is the primary driver of 10-year CVD risk, younger individuals with a high-risk factor burden may remain below treatment thresholds based on 10-year risk estimates. However, the potential long-term gain in CVD-free life expectancy from preventive treatment in such individuals may be substantial.^5^ Conversely, older individuals often have high 10-year CVD risks, but can have limited treatment benefit due to shorter remaining life expectancy. Use of lifetime risk predictions and associated projected lifetime benefits of preventive therapies can therefore support patient–doctor communication and shared decision-making, an approach that is especially recommended for younger people in the 2021 ESC CVD prevention guidelines.^4^

For apparently healthy individuals, the previously published LIFE-CVD model was derived in the MESA study to predict 10-year and lifetime CVD risk.^5^ Risk prediction algorithms developed in one population may over- or underestimate risk in another population (i.e. they may not be well ‘calibrated’), since CVD event rates and average risk factor levels vary with time and geographic region. Performance can be improved by statistical adjustment (recalibration) to contemporary CVD event rates and risk factor levels.^6^ No existing risk prediction model for estimation of lifetime CVD risk has been systematically recalibrated to estimate risk in different regions of Europe.

Here, we describe the development of the LIFEtime-perspective CardioVascular Disease 2 (LIFE-CVD2) model, which is a major update of the previous LIFE-CVD model in two ways.^5^ First, additional data sets from multiple countries are included in model derivation to ensure generalizability; second, the models were recalibrated to four European risk regions using contemporary and representative risk factor levels and CVD incidence. The recalibration approach was aligned with similar methods used for 10-year CVD risk algorithms currently recommended by international guidelines.^7–9^

## Methods

### Study design

**Figure 1:**
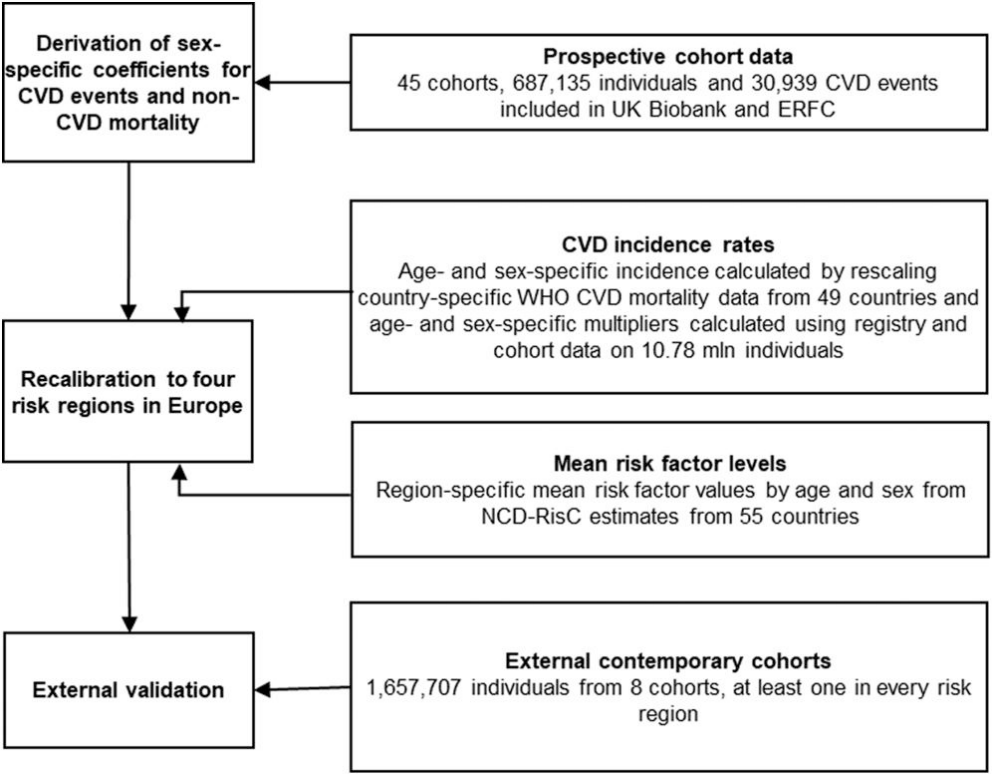
Study design. CVD, cardiovascular disease; ERFC, Emerging Risk Factor Collaboration; WHO, World Health Organization; NCD-RisC, NCD Risk Factor Collaboration.

The updated LIFE-CVD2 model involved multiple data sources for all stages of the project (*Figure 1*). First, to enable reliable estimation of age- and sex-specific relative risks, prediction models for cardiovascular events and non-CVD mortality were derived using individual participant data from 44 prospective cohorts involving 687 135 participants in 13 countries, including the MESA study in which the original LIFE-CVD model was derived. Second, to adapt risk prediction models to the circumstances of four European risk region (grouped on age- and sex-standardized CVD mortality rates, as with the SCORE2 risk algorithms),^8^ the derived risk models were recalibrated using estimated contemporary age- and sex-specific incidences and risk factor distributions. Third, to enhance validity and generalizability, we completed external validation using individual participant data from a further eight European prospective cohorts (i.e. studies not used in the model derivation) from seven countries, involving 1 657 707 participants without prior CVD.

### Derivation and internal validation data

The updated LIFE-CVD2 model was derived using individual participant data from cohorts included in the Emerging Risk Factor Collaboration (ERFC) and the UK Biobank (UKB).^10,11^ The ERFC has collated and harmonized individual participant data from many long-term prospective cohort studies of CVD risk factors and outcomes. Prospective studies in the ERFC were included in this analysis if they met all the following criteria: had recorded baseline information on risk factors necessary to derive risk prediction models [age, sex, current smoking status (vs. never of former smoking), history of diabetes mellitus (DM), systolic blood pressure (SBP), and total and HDL cholesterol]; were approximately population based [i.e. did not select participants on the basis of having previous disease (e.g. case–control studies) and were not active treatment arms of intervention studies]; had a median year of baseline survey after 1990; and had recorded cause-specific deaths and/or non-fatal CVD events (i.e. non-fatal myocardial infarction or stroke) for at least 1 year of median follow-up. The UKB is a single large prospective cohort study with individual participant data on ∼500 000 participants aged >40 years recruited across 23 UK-based assessment centres during 2006–10 and followed-up for cause-specific morbidity and mortality through linkages to routinely available national data sets and disease-specific registers. Individuals with prior CVD at baseline were excluded from model derivation. Details of contributing cohorts are provided in Supplementary material online, *Table S1*.

### External validation data

For external validation, eight additional data sources were used: the Clinical Practice Research Datalink (CPRD, UK, low-risk region), the Extramural LUMC Academic Network (ELAN, the Netherlands, low-risk region),^12^ Heinz Nixdorf Recall study (HNR, Germany, moderate-risk region),^13^ the Estonian Biobank (high-risk region),^14^ and the Health, Alcohol and Psychosocial factors In Eastern Europe study [HAPIEE, including cohorts from Poland and the Czech Republic (high-risk region), Russia, and Lithuania (very high-risk region)].^15^

The CPRD is a UK-based primary care database of anonymized medical records from 674 general practices, with coverage of over 11.3 million patients, and is broadly representative of the general population in terms of age, sex, and ethnicity. The data used for this study are restricted to the region of England with baseline during the period 1 April 2004–2006 and follow-up to 30 November 2017. Incident non-fatal events are obtained from linkage with Hospital Episode Statistics and deaths from the Office for National Statistics. ELAN is a prospective, population-based study using routine healthcare data from general practitioners with a baseline in 2010, linked to hospital and registry data from Statistics Netherlands, in the region of The Hague and Leiden, the Netherlands. HNR is a population-based study in the large, heavily industrialized Ruhr area, Germany. From December 2000 to August 2003, random samples of men and women aged 45–75 were drawn from mandatory residency lists of three cities in the Ruhr area of north-west Germany. The Estonian Biobank is a population-based biobank of the Estonian Genome Center of the University of Tartu. Follow-up of incident fatal and non-fatal coronary heart disease and stroke events of a subset of the cohort is on-going as our database is being linked with the national healthcare registries and regional and central hospital databases. The HAPIEE study comprises four prospective urban population-based cohorts from Eastern Europe, located in Novosibirsk (Russia), Krakow (Poland), Kaunas (Lithuania), and six cities of the Czech Republic. Each cohort recruited a random sample of men and women aged 45–69 years at baseline conducted in 2002–05 (2005–08 in Lithuania), stratified by sex and 5-year age groups. From these cohorts, all individuals aged 35 and older without prior CVD or DM were included.

### Statistical analysis

The LIFE-CVD2 model comprised complimentary sex-specific Cox proportional hazards models for cardiovascular events and non-CVD mortality, respectively. These models used age as the time axis (i.e. left truncation), were stratified by cohort, and included the same predictors as the ESC recommended SCORE2 and SCORE2-OP 10-year risk models,^8^ namely age, SBP, total and HDL cholesterol, current smoking status, and DM and their interactions with age at entry. While the LIFE-CVD2 risk models are not intended for use in individuals with diabetes, participants with a history of diabetes were included at the model derivation stage (with appropriate adjustment for diabetes status), since it was not possible to exclude people with diabetes from population-level mortality statistics and risk factor data used in recalibration. Lifetime predictions are generated by calculating individual participant specific cumulative survival estimates from the CVD and non-CVD models repetitively in 1-year windows across all future life years using a life table approach,^16^ which incorporates adjustment for the competing risk of non-CVD death. This approach has been shown to yield accurate CVD risk predictions beyond the original cohort follow-up for up to 17-year risk predictions.^16^ The CVD-free life expectancy can be read from this life table and is defined as the median survival without a CVD event or death, the age at which the cumulative survival probability becomes <0.5. The 45–90-year age range of the original LIFE-CVD algorithm has been extended to 35–100 years for LIFE-CVD2 to allow for lifetime predictions for younger individuals and to be able to directly model beyond a survival probability of <0.5 for older individuals. Lifetime risk was defined as the risk of having a CVD event before the age of 80 years of life and was calculated by summing all yearly predicted event risks from the current age up until the age of 80 years. Since not all data sources included in the derivation data had follow-up across the complete LIFE-CVD2 age range, the ‘un-calibrated’ baseline hazard was based upon the low-risk region incidence data [World Health Organization (WHO) CVD mortality rates converted to CVD incidence] to ensure smooth baseline survival curves. The primary outcome predicted by LIFE-CVD2 was defined as a composite of cardiovascular mortality, non-fatal myocardial infarction, and non-fatal stroke. The competing non-CVD endpoint was defined as death from any non-cardiovascular cause not included in the primary outcome. Details of the different ICD-10 codes included in both the fatal and non-fatal components of the endpoint are provided in Supplementary material online, *Table S2*.

LIFE-CVD2 was recalibrated to four risk regions of Europe using methods and values previously applied for recalibration of the SCORE2 and SCORE2-OP models, with adaptation for the lifetime risk context (see Supplementary material online, *Methods*).^8,9^ Briefly, risk regions were defined according to CVD mortality obtained from WHO mortality statistics (see Supplementary material online, *Figure S1*) and recalibration involved the use of region-, 5 year age group-, and sex-specific CVD incidence, non-CVD mortality incidence, and average risk factor values. To obtain CVD incidence estimates that included non-fatal as well as fatal events, annual CVD mortality rates derived from the WHO mortality database^17^ were converted to total CVD rates using a ‘multiplier approach’, applying multipliers previously published for the SCORE2 and SCORE2-OP risk algorithms.^8,9^ For non-CVD deaths, annual mortality rates derived from the WHO mortality database^8,9^ corrected for the presence of individuals with established ASCVD in national mortality statistics using similar multiplier approach. Age group- and sex-specific risk factor values were obtained from the Non-Communicable Disease Risk Factor Collaboration (NCD-RisC).^18,19^ Sex-specific recalibration of both the CVD and non-CVD mortality risk models was performed by regressing the observed annual risks on the model predicted 1-year risks (using age group-specific risk factor values) across 5-year age groups (see Supplementary material online, *Methods*). The recalibrated CVD and non-CVD death risks were then combined in the life table.

Discrimination was assessed using Harrell’s C-indices corrected for competing risks.^20^ Calibration was assessed by visual inspection of predicted vs. observed risk plots in deciles of predicted risk using external validation data from CPRD and ELAN, as these were the only cohorts deemed approximately nationally representative.^20^ Since none of the cohorts used for external validation had lifelong follow-up, discrimination and calibration were assessed at 10 years of follow-up, or at the latest full year with at least 80% of the individuals still under follow-up, using time in study as the timescale. Calibration of lifetime risk predictions was also assessed in CPRD using age as the timescale.

The handling of missing data is more extensively described in the Supplementary material online, *Methods*. In brief, predictors were imputed by single regression imputation with predictive mean matching for all cohort data. As the routine care data sources (CPRD and ELAN) had much higher rates of missing data, which were more likely to be associated to CVD outcomes, these were handled using multiple imputations with fully conditional specification in five imputed data sets. All analyses were performed with R statistical programming (version 3.5.2, R Foundation for Statistical Computing, Vienna, Austria) or Stata (version 15.1, StataCorp, College Station, TX, USA). All R and Stata code is available from the corresponding author upon request.

### Estimation of preventive intervention effects

The alteration of patient characteristics in individual predictions to simulate treatment effects provides an observational, rather than causal, assessment of risk factor changes.^21^ Therefore, to estimate the lifelong effect of risk factor management (blood pressure lowering, lipid lowering, and smoking cessation), causal evidence was used from trials and meta-analyses,^5,21^ which were combined with the annual risks of CVD events and non-CVD mortality as estimated in the updated LIFE-CVD2 model. This process is described in detail in the Supplementary material online, *Methods*. Accordingly, the potential gain in CVD-free life expectancy was estimated by applying causal hazard ratio (HR) of 0.78 per 1 mmol/L reduction in LDL cholesterol^22,23^ and 0.80 per 10 mmHg SBP reduction^24^ to the CVD event rates. The benefit of smoking cessation was applied using an HR of 0.60 for CVD event rates and 0.73 for non-CVD mortality rates.^25,26^ A stable HR was assumed over time in the examples of the lifetime benefit of risk factor reduction.

## Results

### Model derivation

Model derivation involved 687 135 individuals without previous CVD recruited between 1990 and 2009 into prospective cohorts in Europe (36 cohorts, 610 353 participants) and North America (9 cohorts, 76 782 participants). Mean age at recruitment was 57 (SD 9) years, and 298 408 (43%) were male (*Table 1*). During median follow-up of 10.7 (5th, 95th percentile: 5.0, 18.5) years, a total of 30 939 CVD events and 34 284 non-CVD deaths were recorded. Model HRs are summarized in *Table 2*, with the unrounded log HRs and baseline hazard shown in Supplementary material online, *Tables S3* and *S4*. The HRs for most risk predictors decreased with increasing age of participants.

**Table 1:** Summary statistics of the model derivation population

	*n* (%) or mean (SD)
Total participants	687 135
Male sex	298 408 (43%)
Age (years)	58 (8)
Current smoker	104 471 (15%)
Systolic blood pressure (mmHg)	135 (19)
Diabetes mellitus	32 234 (4.7%)
Total cholesterol (mmol/L)	5.9 (1.1)
HDL cholesterol (mmol/L)	1.4 (0.4)
Follow-up (years, 5th/95th percentile)	10.7 (5.0–18.5)
Cardiovascular events	30 939
Non-cardiovascular deaths	34 284

**Table 2:** Hazard ratios of the LIFE-CVD2 models

	Men		Women	
Models to predict CVD events	Main effect	Age interaction term (per 5 years)	Main effect	Age interaction term (per 5 years)
Age (per 5 years)*	1.10 (1.08, 1.13)		1.13 (1.10, 1.16)	
Current smoking (vs. never/former)	1.90 (1.83, 1.97)	0.94 (0.92, 0.96)	2.27 (2.17, 2.38)	0.90 (0.88, 0.93)
SBP (per 20 mmHg)	1.34 (1.31, 1.36)	0.96 (0.96, 0.97)	1.40 (1.37, 1.43)	0.95 (0.94, 0.96)
Total cholesterol (per 1 mmol/L)	1.16 (1.15, 1.18)	0.97 (0.96, 0.98)	1.11 (1.09, 1.13)	0.97 (0.96, 0.98)
HDL cholesterol (per 0.5 mmol/L)	0.76 (0.74, 0.78)	1.04 (1.03, 1.06)	0.78 (0.76, 0.80)	1.04 (1.03, 1.05)
History of diabetes mellitus^a^	1.94 (1.84, 2.04)	0.91 (0.89, 0.94)	2.35 (2.21, 2.50)	0.89 (0.86, 0.91)

Models to predict non-CVD mortality	Main effect	Age interaction term (per 5 years)	Main effect	Age interaction term (per 5 years)
Age (per 5 years)*	0.99 (0.97, 1.02)		0.90 (0.88, 0.92)	
Current smoking (vs. never/former)	2.22 (2.14, 2.30)	0.93 (0.92, 0.95)	2.18 (2.10, 2.27)	0.98 (0.97, 1.00)
SBP (per 20 mmHg)	1.08 (1.06, 1.10)	0.96 (0.96, 0.97)	1.06 (1.04, 1.08)	0.98 (0.97, 0.99)
Total cholesterol (per 1 mmol/L)	0.92 (0.90, 0.93)	1.00 (0.99, 1.01)	0.95 (0.94, 0.97)	0.98 (0.98, 0.99)
HDL cholesterol (per 0.5 mmol/L)	1.10 (1.08, 1.13)	0.98 (0.97, 0.99)	0.95 (0.93, 0.97)	1.01 (1.00, 1.03)
History of diabetes mellitus^a^	1.61 (1.52, 1.70)	0.92 (0.89, 0.94)	1.72 (1.61, 1.83)	0.94 (0.91, 0.97)

Sex-specific hazard ratios from the LIFE-CVD2 models predicting the risk of fatal and non-fatal CVD events or the risk of non-CVD mortality. (Baseline) age was centred at 60 years, systolic blood pressure at 120 mmHg, total cholesterol at 6 mmol/L, and HDL cholesterol at 1.3 mmol/L.

SBP, systolic blood pressure.

^a^Diabetes mellitus was included in the modelling as diabetes patients are included in the recalibration data. For use in clinical practice, this coefficient should be ignored.

Using the age-, sex-, and region-specific mean risk factor levels and incidence data, the LIFE-CVD2 model was recalibrated to four European risk regions (see Supplementary material online, *Figure S2*). After recalibration, predicted risks based on mean risk factor levels showed good agreement with the expected incidences of CVD event and non-CVD mortality (see Supplementary material online, *Figures S3* and *S4*) and were also similar to incidence rates obtained from external national registries (see Supplementary material online, *Figure S5*). Regional sex- and age-specific multipliers for conversion of CVD mortality rates to incidence rates and for correction of those with prior CVD in national mortality statistics are shown in Supplementary material online, *Figures S6 and S7*. The ratio between the 1-year cumulative incidence of total to fatal CVD was similar to the ratio at 10 years (see Supplementary material online, *Figure S8*) supporting the use of the SCORE2 project multipliers previously estimated to convert 10-year CVD mortality to total CVD event risk.^8^

### External validation

**Figure 2:**
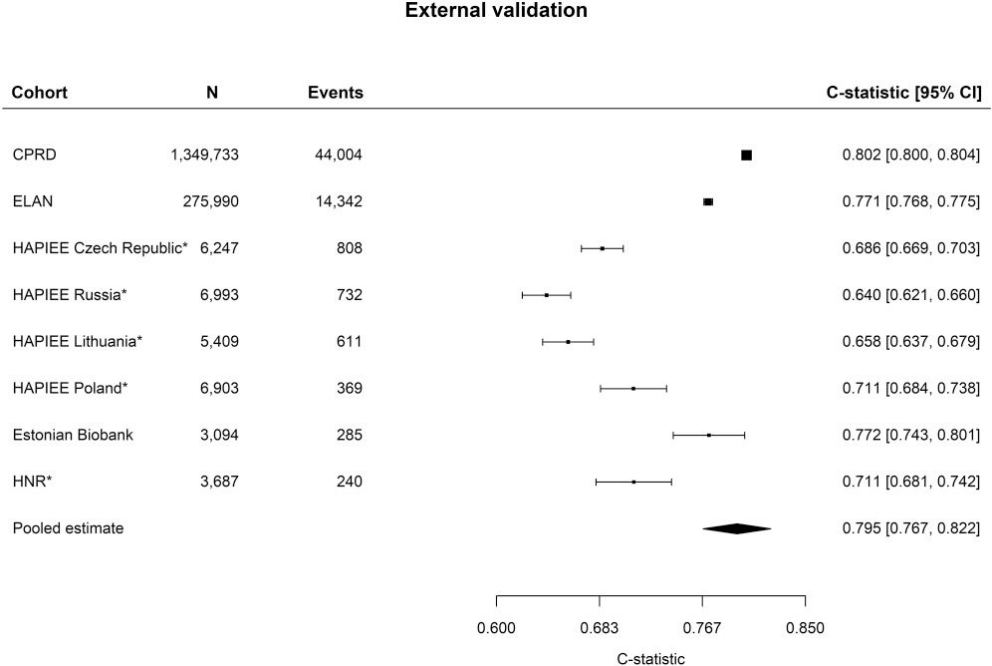
C-index of the recalibrated LIFE-CVD model to discriminate in external validation cohorts upon assessing cardiovascular disease events. *As discrimination result may be underestimated because the entire age range of the LIFE-CVD2 model could not be included from this cohort, this result is not included in the shown pooled estimate. The overall pooled estimate including all studies is 0.789 (95% confidence interval 0.703–0.875).

**Figure 3:**
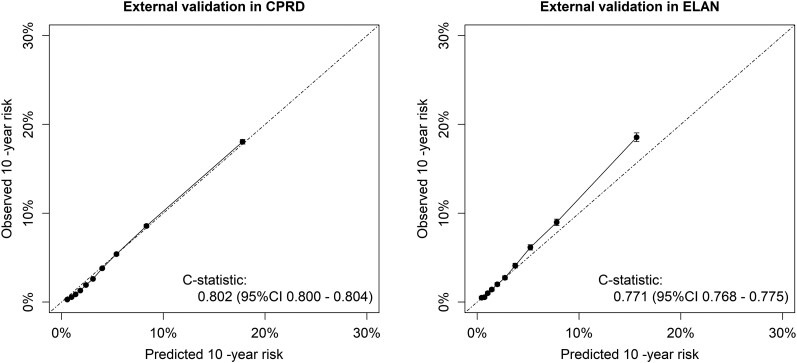
Calibration for prediction of cardiovascular disease events of the recalibrated LIFE-CVD2 model in Clinical Practice Research Datalink (*n* = 1 349 377) and Extramural LUMC Academic Network (*n* = 275 990). Predicted vs. observed cardiovascular disease risks in deciles of predicted risk for the recalibrated LIFE-CVD2 model.

External validation involved data from 1 657 707 individuals without previous CVD or DM from eight European cohorts. Of these individuals, 793 454 were male (48%) and the mean (SD) ages per cohort ranged from 48 (13) years in the Estonian Biobank to 59 (6) years in HNR (see Supplementary material online, *Table S6*). The median follow-up times per cohort ranged from 6.3 years [interquartile range (IQR) 6.0–6.9] in HAPIEE Poland to 14.0 years (IQR 10.5–15.6) in HNR. During this follow-up, a total of 61 311 CVD events and 65 867 non-CVD deaths were recorded. The pooled C-index for the prediction of CVD events was 0.795 [95% confidence interval (CI) 0.767–0.822] in the studies including the full LIFE-CVD2 age range, and the overall pooled estimate including all studies was 0.789 (95% CI 0.703–0.875; *Figure 2*). The pooled C-index for the competing endpoint of non-CVD mortality was 0.831 (95% CI 0.810–0.852) in the studies with the full age range and 0.824 (95% CI 0.711–0.937) when including all external validation studies (see Supplementary material online, *Figure S9*). C-indices for both endpoints were lower for cohorts that did not include patients over the complete age range at baseline. In the CPRD and ELAN data, the predicted 10-year CVD event risks agreed well with the observed risks (*Figure 3*), whereas there was some overestimation of non-CVD mortality risk in ELAN (see Supplementary material online, *Figure S10*). Sensitivity analysis evaluating the accuracy of lifetime risks in CPRD showed good agreement between predicted lifetime risk and the observed lifetime incidence of CVD for both men and women (see Supplementary material online, *Figure S11*). The LIFE-CVD2 individual predicted risks were well aligned with SCORE2 predictions. For example, among individuals below 70 in CPRD, the median difference between the SCORE2 and LIFE-CVD2 10-year predictions was −0.15% (IQR −0.36%; −0.02%).

### Estimation of treatment effects

**Figure 4:**
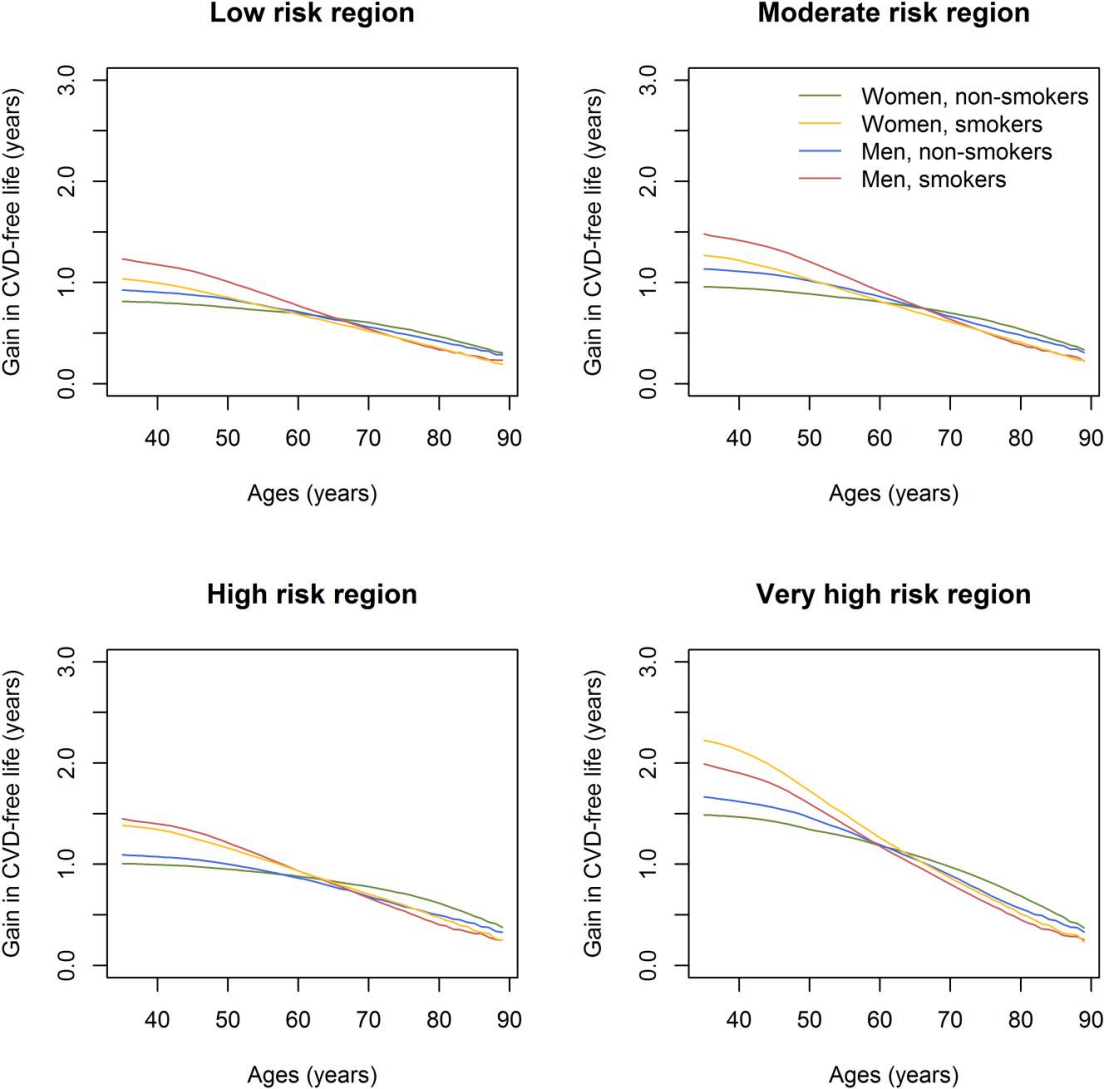
Predicted gain in cardiovascular disease–free life expectancy from 10 mmHg blood pressure reduction for an individual with total cholesterol concentrations of 5.5 mmol/L, HDL cholesterol of 1.3 mmol/L, and systolic blood pressure of 140 mmHg, for each region, stratified on smoking status.

*Figure 4* shows how the estimated gain in CVD-free life expectancy, conditional on age, from lifelong 10 mmHg blood pressure reduction estimated by the LIFE-CVD2 model differs across regions for an individual person with a SBP of 140 mmHg, total cholesterol of 5.5 mmol/L, and HDL cholesterol of 1.3 mmol/L. The estimated gain in CVD-free life expectancy in 40-year-old, non-smoking men ranged from 0.9 years in low-risk countries to 1.7 years in very high-risk countries. In women, it ranged from 0.9 years in low-risk countries to 1.6 years in very high-risk countries (*Figure 4*). In comparison, the individual gain in CVD-free life expectancy from smoking cessation in the same 40-year women ranged from 4.2 years in the low-risk region up to 4.8 years for 40-year-olds in the very high-risk region, and for 40-year-old men from 5.0 in the low-risk region up to 6.3 year in the very high-risk region (see Supplementary material online, *Figure S12*). An example of several 10-year and lifetime prediction measures has been illustrated across different ages in Supplementary material online, *Figure S13*.

The estimated individual gain in CVD-free life expectancy for each combination of risk factor levels is displayed in 2D risk charts in Supplementary material online, *Appendix S1*, showing the examples of 40% mmol/L LDL cholesterol reduction, SBP reduction to <140 mmHg, and smoking cessation in each of the four European risk regions. The highest gains in CVD-free life expectancy from prevention are in younger individuals with adverse risk factor values, who are often below recommended treatment thresholds. For example, a non-smoking 52-year-old woman, with a SBP of 150 mmHg, total cholesterol of 6.0 mmol/L, and HDL cholesterol of 1.1 mmol/L, would have SCORE2 10-year risks of 3.0% in the low-risk region ranging up until 8.2% in the very high-risk region, all below the guideline-recommended treatment thresholds.^4,8^ Based on the LIFE-CVD2 model, her gain in CVD-free life expectancy from 1 mmol/L LDL reduction would range from 1.1 years in the low-risk region until 1.9 years in the very high-risk region—a higher benefit than several other individuals above 10-year risk-based treatment thresholds (see Supplementary material online, *Appendix S1*).^4^

## Discussion

This report describes the development of LIFE-CVD2, a risk model to estimate lifetime risk and CVD-free life expectancy in individuals without previous CVD or DM in four European risk regions. It also demonstrates how LIFE-CVD2 can be used to illustrate potential gains in CVD-free life expectancy in response to preventative intervention, given reasonable assumptions about intervention effects. Recalibration was completed using an approach adapted from SCORE2 to develop 10-year CVD risk models for the same four risk regions. External validation was performed across different risk regions, and estimations of lifetime treatment benefits were illustrated for several risk factor profiles. The updated LIFE-CVD2 model confers several advantages over the originally published version of the model (LIFE-CVD).

First, it is systematically recalibrated using contemporary and representative data on CVD incidence and risk factor data, which broaden the generalizability of the LIFE-CVD2 model across European risk regions. Because the recalibration approach was based on registry data, the model can be readily updated to reflect future disease CVD incidences and risk factor profiles as soon as new updated data become available.^7,8^ Furthermore, since the recalibration approach was aligned with that used in the 10-year CVD SCORE2 and SCORE2-OP risk prediction models, synergy between 10-year and lifetime risk assessment is ensured.

Second, because models have been derived and recalibrated to be sex specific, LIFE-CVD2 is well adapted to the contemporary clinical practice for both sexes. The original LIFE-CVD model was not derived and recalibrated separately for both sexes, ignoring differences in the relative effects of certain predictors, and different evolution of risk with age between men and women.

Third, the models have been derived again using powerful, contemporary data from multiple different studies and registry sources. This enhances the accuracy, generalizability and validity of the approach. In particular, data on a total of >12.5 million individuals from dozens of countries were used for the development and recalibration of LIFE-CVD2.

Fourth, the age range of the model has been extended from 45–90 to the age range of 35–100 years. This allows the model to be applied to individuals with a current age between 35 and 90 years and improves the stability of estimates in people of all ages with a very high life expectancy. As the worldwide life expectancy continues to rise,^27^ this will be increasingly important.

Fifth, to improve risk communication, the lifetime treatment benefit, defined as the gain in CVD-free life expectancy from preventive therapy, can be estimated with the LIFE-CVD2 model. This has been shown to be an intuitive measure that lowers the decisional conflict among individuals considering preventive treatment.^28^ When using lifetime treatment benefit measures in the shared decision process, these should be weighed against the intended treatment duration. All of these of risk estimates should be taken into account when considering treatment initiation and should be used in conjunction with assessing potential risk modifiers relevant to the specific patient as well as patient preferences.

Finally, the current study highlights the importantly different expected gain in CVD-free life expectancy differed across geographical locations in Europe. This has been incorporated into benefit estimation by the integral recalibration step in model development, something other lifetime risk models have not considered.^21,29^ Our results have shown that interventions are expected to lead to a higher absolute treatment benefit in Eastern European countries, reflecting higher disease incidences in this region.

The LIFE-CVD2 model can be used in a simplified form via the 2D risk charts as provided in Supplementary material online, *Appendix S1*, predicting the benefit from lifelong lipid lowering, blood pressure lowering, and smoking cessation. However, to accommodate more accurate predictions and to calculate a wider range of possible treatment options, the LIFE-CVD2 model will be integrated in the CE-marked U-Prevent medical device, available from www.U-Prevent.com. Because of the time required for implementation in a CE-marked medical device, the LIFE-CVD2 model is integrated in an R-shiny app for scientific purposes only (i.e. no clinical use) from https://hagemanshj.shinyapps.io/LIFECVD2/.

The potential limitations of this study merit consideration. Calibration of the LIFE-CVD2 model was only assessed in the large nationally representative data set from the CPRD and ELAN, because the other cohorts used for external validation do not necessarily reflect contemporary absolute risk levels across European regions (all cohort data, including the cohorts involved, may not be nationally representative, reflecting past periods of time or self-selected participants such as healthy volunteers). In the CPRD and ELAN, however, good agreement was observed between 10-year predicted and observed CVD incidences. Furthermore, estimated CVD rates agreed well with national incidence rates from available independent external registries from several countries and the discrimination was evaluated in all European risk regions.

Another potential limitation of the current study is that data on medication use, family history, socio-economic status, nutrition, physical activity, renal function, or ethnicity were not available in cohorts and registries used for model derivation and recalibration. Hence, interpretation of LIFE-CVD2 estimates may require clinical judgement, especially for individuals in whom these factors may be relevant.

For the derivation of the LIFE-CVD2 model, data from relatively high-income countries were included. Ideally, however, the derivation of risk models for use in high- and very high-risk countries would have involved large nationally representative, prospective cohorts in these countries, coupled with prolonged follow-up and validation of fatal and non-fatal CVD endpoints. Unfortunately, such data do not yet generally exist. Indeed, even in low- and moderate-risk regions, the cohorts involved may not be nationally representative, reflecting past periods of time or self-selected participants such as healthy volunteers. While healthy volunteer bias can lead to low estimates of absolute risk, relative risks are generally unaffected.^7^ Furthermore, our approach makes the assumption that the relative risks obtained in the derivation data set are transferable across different populations, which was further supported by the satisfactory discrimination results in high- and very high-risk region validation cohorts as observed in the current study.

Another potential limitation is the fact that validation was mostly performed with 10-year risks, as it is not feasible to perform validation of life expectancy measures within the scope of cohort follow-up durations. In CPRD, sensitivity analyses using age as the timescale were performed and showed adequate agreement between predicted and observed lifetime risks. However, similar methodological adaptations to calculate discrimination are not possible when taking a lifetime perspective. Previous studies have shown the validity of CVD risk predictions for up to 17 years.^16^ When longer-term data become available, the model could profit from validations at even longer timescales to further validate the underlying methodology.

## Conclusions

In conclusion, by taking into account geographical differences in CVD incidence, the recalibrated LIFE-CVD2 model provides a more accurate tool for the prediction of lifetime risk and CVD-free life expectancy for individuals without previous CVD across Europe, ensuring synergy with 10-year risk estimation and facilitating shared decision-making on Step 2 cardiovascular prevention options as recommended by the 2021 European prevention guidelines.

## Supplementary Material

zwae174_Supplementary_Data

## Data Availability

The data underlying this article were provided by representatives of all included cohorts. Data from each cohort may be shared on request to the respective representatives, depending on cohort-specific policies. All R and Stata code is available from the corresponding author upon request.

## Appendix

### Investigators of the Emerging Risk Factors Collaboration

**ATENA:** Salvatore Panico, Giovanni Veronesi, and Chiara Donfrancesco; **ATTICA:** Christina Chrysohoou and Christos Pitsavos; **ARIC:** Anna Kucharska-Newton and Kunihiro Matsushita; **BRUN:** Gregorio Rungger, Christoph Leitner, and Johann Willeit; **BWHHS:** Julie Taylor, Caroline Dale, and Floriaan Schmidt; **CHS1:** Bruce M. Psaty and James S. Floyd; **CHS2:** Bruce M. Psaty and James S. Floyd; **COPEN:** Marianne Benn, Anne Langsted, and Pia R. Kamstrup; **DESIR:** Beverley Balkau, Fabrice Bonnet, and Frederic Fumeron; **DRECE:** Pilar Cancelas-Navia, Cristina Martín-Arriscado, and Miguel Menéndez-Orenga; **EMOFRI:** Salvatore Panico, Giovanni Veronesi, and Chiara Donfrancesco; **EPICNOR:** Nicholas J. Wareham; **ESTHER:** Bernd Holleczek, Kira Trares, and Hannah Stocker; **FINNMARK:** Inger Ariansen; **GOTO43:** George Lappas; **HCS:** Cyrus Cooper and Nicholas Harvey; **HONOL:** Beatriz Rodriguez; **HOORN:** Femke Rutters and Marieke T. Blom; **HUBRO:** Inger Ariansen; **LEADER:** Jackie A. Cooper; **MATISS93:** Benedetta Marcozzi, Cecilia Damiano, and Cinzia Lo Noce; **MESA:** Karol Watson, Wendy Post, and James Pankow; **MIDFAM:** Naveed Sattar; **MONFRI94:** Salvatore Panico, Giovanni Veronesi, and Chiara Donfrancesco; **MONICA_KORA:** Annette Peters and Barbara Thorand; **MORGEN:** Anneke Blokstra; **MOSWEGOT:** George Lappas; **NHANESIII:** Richard F. Gillum; **NSHS:** Karina W. Davidson and Joseph E. Schwartz; **OPPHED:** Inger Ariansen; **OSLO2:** Lise Lund Håheim; **PREVEND:** Harold Snieder and Kevin Damman; **ProspectEPIC:** Karel G.M. Moons; **PROSPER:** Stella Trompet, Chris Packard, and Ian Ford; **RS_I:** M. Arfan Ikram, Banafsheh Arshi, and Elif Aribas; **RS_II:** M. Arfan Ikram, Banafsheh Arshi, and Elif Aribas; **SHIP:** Henry Völzke, Marcello P. Markus, Carsten O. Schmidt, and Stephan B. Felix; **TROMS:** Inger Ariansen; **USPHS2:** Julie Buring; **WHITEII:** Eric J. Brunner and Mika Kivimaki; **WHITEI:** Eric J. Brunner and Mika Kivimaki; **WHS:** Julie Buring; **WOSCOPS:** Michele Robertson, Robin Young, and Peter Macfarlane; **ZARAGOZA:** Alejandro Marín Ibañez.
