# Correction to: Prediction of individual lifetime cardiovascular risk and potential treatment benefit: development and recalibration of the LIFE-CVD2 model to four European risk regions

**DOI:** 10.1093/eurjpc/zwae274

**Published:** 2024-08-27

This is a **correction** to:

Steven H J Hageman, Stephen Kaptoge, Tamar I de Vries, Wentian Lu, Janet M Kist, Hendrikus J A van Os, Mattijs E Numans, Kristi Läll, Martin Bobak, Hynek Pikhart, Ruzena Kubinova, Sofia Malyutina, Andrzej Pająk, Abdonas Tamosiunas, Raimund Erbel, Andreas Stang, Börge Schmidt, Sara Schramm, Thomas R Bolton, Sarah Spackman, Stephan J L Bakker, Michael Blaha, Jolanda M A Boer, Amélie Bonnefond, Hermann Brenner, Eric J Brunner, Nancy R Cook, Karina Davidson, Elaine Dennison, Chiara Donfrancesco, Marcus Dörr, James S Floyd, Ian Ford, Michael Fu, Ron T Gansevoort, Simona Giampaoli, Richard F Gillum, Agustín Gómez-de-la-Cámara, Lise Lund Håheim, Per-Olof Hansson, Peter Harms, Steve E Humphries, M Kamran Ikram, J Wouter Jukema, Maryam Kavousi, Stefan Kiechl, Anna Kucharska-Newton, David Lora Pablos, Kunihiro Matsushita, Haakon E Meyer, Karel G M Moons, Martin Bødtker Mortensen, Mirthe Muilwijk, Børge G Nordestgaard, Chris Packard, Luigi Pamieri, Demosthenes Panagiotakos, Annette Peters, Louis Potier, Rui Providencia, Bruce M Psaty, Paul M Ridker, Beatriz Rodriguez, Annika Rosengren, Naveed Sattar, Ben Schöttker, Joseph E Schwartz, Steven Shea, Martin J Shipley, Reecha Sofat, Barbara Thorand, W M Monique Verschuren, Henry Völzke, Nicholas J Wareham, Leo Westbury, Peter Willeit, Bin Zhou, John Danesh, Frank L J Visseren, Emanuele Di Angelantonio, Lisa Pennells, Jannick A N Dorresteijn, Prediction of individual lifetime cardiovascular risk and potential treatment benefit: development and recalibration of the LIFE-CVD2 model to four European risk regions, *European Journal of Preventive Cardiology*, Volume 31, Issue 14, October 2024, Pages 1690–1699, https://doi.org/10.1093/eurjpc/zwae174

Names of the authors Stephen Kaptoge, Jolanda M.A. Boer and Paul M. Ridker have been corrected.

