# Supplementary material for: Prediction of individual lifetime cardiovascular risk and potential treatment benefit: development and recalibration of the LIFE-CVD2 model to four European risk regions
Source: Eur J Prev Cardiol. 2024 May 16;31(14):1690–9. doi: 10.1093/eurjpc/zwae174 (PMC11464100; doi:10.1093/eurjpc/zwae174)
Supplement: zwae174_Supplementary_Data [file zwae174_supplementary_data.zip › Appendix_1_sbp_withtitleandrisk-gecombineerd.pdf.pdf]

Geographic recalibration to four European risk regions of the LIFE-CVD model for prediction of lifetime risk and individual lifetime treatment benefit in apparently healthy people

Appendix 1

LIFE-CVD2 model

Lifetime risk in the low risk region (risk from current age up to age 80 years)

Lifetime risk until 80	
Green	<20%
Orange	20 to <30%
Red	≥30%

Age		Women								Men							
		Non-smoking				Smoking				Non-smoking				Smoking			
		3.0-3.9	4.0-4.9	5.0-5.9	6.0-6.9	3.0-3.9	4.0-4.9	5.0-5.9	6.0-6.9	3.0-3.9	4.0-4.9	5.0-5.9	6.0-6.9	3.0-3.9	4.0-4.9	5.0-5.9	6.0-6.9
75 - 79	160-179	4%	4%	4%	4%	5%	5%	5%	5%	5%	5%	6%	6%	7%	7%	8%	8%
	140-159	3%	3%	3%	3%	5%	5%	5%	5%	5%	5%	5%	5%	6%	6%	7%	7%
	120-139	3%	3%	3%	3%	4%	4%	4%	4%	4%	4%	4%	5%	5%	6%	6%	6%
	100-119	3%	3%	3%	3%	4%	4%	4%	4%	4%	4%	4%	4%	5%	5%	5%	5%
70 - 74	160-179	8%	8%	8%	8%	11%	11%	11%	11%	11%	11%	12%	13%	14%	15%	16%	17%
	140-159	7%	7%	7%	7%	9%	10%	10%	10%	9%	10%	10%	11%	12%	13%	14%	15%
	120-139	6%	6%	6%	6%	8%	8%	9%	9%	8%	9%	9%	10%	11%	12%	12%	13%
	100-119	5%	6%	6%	6%	7%	7%	8%	8%	7%	8%	8%	8%	10%	10%	11%	11%
65 - 69	160-179	11%	11%	11%	11%	14%	14%	15%	15%	14%	15%	16%	17%	19%	20%	22%	23%
	140-159	9%	9%	10%	10%	12%	13%	13%	13%	13%	13%	14%	15%	16%	18%	19%	20%
	120-139	8%	8%	8%	8%	11%	11%	11%	12%	11%	12%	12%	13%	14%	15%	16%	18%
	100-119	8%	8%	8%	9%	11%	11%	12%	12%	11%	12%	13%	13%	14%	15%	17%	18%
60 - 64	160-179	12%	13%	13%	13%	17%	17%	18%	19%	17%	18%	20%	21%	22%	24%	26%	28%
	140-159	11%	11%	11%	12%	14%	15%	16%	16%	15%	16%	17%	18%	19%	21%	22%	24%
	120-139	9%	10%	10%	10%	12%	13%	13%	14%	13%	14%	15%	16%	17%	18%	19%	21%
	100-119	8%	8%	8%	9%	11%	11%	12%	12%	11%	12%	13%	13%	14%	15%	17%	18%
55 - 59	160-179	14%	14%	15%	15%	19%	20%	20%	21%	19%	21%	22%	24%	25%	27%	29%	32%
	140-159	12%	12%	13%	13%	16%	17%	18%	18%	16%	18%	19%	21%	21%	23%	25%	27%
	120-139	10%	10%	11%	11%	14%	14%	15%	16%	14%	15%	16%	18%	18%	20%	22%	24%
	100-119	9%	9%	9%	10%	12%	12%	13%	13%	12%	13%	14%	15%	16%	17%	19%	20%
50 - 54	160-179	15%	15%	16%	17%	20%	22%	23%	24%	21%	23%	24%	27%	27%	29%	32%	35%
	140-159	13%	13%	14%	14%	17%	18%	19%	20%	18%	19%	21%	23%	23%	25%	28%	30%
	120-139	11%	11%	12%	12%	15%	16%	16%	17%	15%	16%	18%	19%	20%	22%	24%	26%
	100-119	9%	10%	10%	10%	13%	13%	14%	15%	13%	14%	15%	16%	17%	19%	20%	22%
45 - 49	160-179	16%	17%	17%	18%	22%	23%	25%	26%	22%	24%	26%	29%	29%	32%	35%	38%
	140-159	13%	14%	15%	15%	19%	20%	21%	22%	19%	20%	22%	24%	25%	27%	30%	33%
	120-139	11%	12%	12%	13%	16%	17%	18%	18%	16%	17%	19%	21%	21%	23%	26%	28%
	100-119	10%	10%	10%	11%	13%	14%	15%	16%	13%	15%	16%	17%	18%	20%	22%	24%
40 - 44	160-179	16%	17%	18%	19%	23%	25%	26%	28%	23%	25%	28%	30%	30%	33%	37%	40%
	140-159	14%	14%	15%	16%	20%	21%	22%	23%	19%	21%	23%	25%	25%	28%	31%	35%
	120-139	12%	12%	13%	13%	16%	17%	18%	20%	16%	18%	20%	21%	22%	24%	27%	29%
	100-119	10%	10%	11%	11%	14%	15%	15%	16%	14%	15%	16%	18%	18%	20%	23%	25%

Non-HDL cholesterol (mmol/L)

150 200 250
mg/dL

LIFE-CVD2 model

Lifetime risk in the moderate risk region (risk from current age up to age 80 years)

Lifetime risk until 80

- Green: <20%
- Yellow: 20 to <30%
- Red: ≥30%

Age	Systolic BP	Women Non-smoking 3.0-3.9	Women Non-smoking 4.0-4.9	Women Non-smoking 5.0-5.9	Women Non-smoking 6.0-6.9	Women Smoking 3.0-3.9	Women Smoking 4.0-4.9	Women Smoking 5.0-5.9	Women Smoking 6.0-6.9	Men Non-smoking 3.0-3.9	Men Non-smoking 4.0-4.9	Men Non-smoking 5.0-5.9	Men Non-smoking 6.0-6.9	Men Smoking 3.0-3.9	Men Smoking 4.0-4.9	Men Smoking 5.0-5.9	Men Smoking 6.0-6.9
75 - 79	160-179	5%	5%	5%	5%	7%	7%	7%	7%	7%	7%	7%	8%	9%	10%	10%	10%
	140-159	4%	4%	4%	4%	6%	6%	6%	6%	6%	6%	6%	7%	8%	9%	9%	9%
	120-139	4%	4%	4%	4%	5%	5%	5%	5%	5%	5%	6%	6%	7%	8%	8%	8%
	100-119	4%	4%	4%	4%	5%	5%	5%	5%	5%	5%	5%	5%	6%	7%	7%	7%
70 - 74	160-179	10%	10%	10%	10%	14%	14%	14%	14%	14%	15%	16%	16%	19%	20%	21%	22%
	140-159	9%	9%	9%	9%	12%	12%	12%	13%	12%	13%	14%	14%	16%	17%	18%	19%
	120-139	8%	8%	8%	8%	11%	11%	11%	11%	11%	11%	12%	13%	14%	15%	16%	17%
	100-119	7%	7%	7%	7%	9%	10%	10%	10%	9%	10%	10%	11%	12%	13%	14%	15%
65 - 69	160-179	13%	14%	14%	14%	18%	18%	19%	19%	19%	20%	21%	22%	24%	26%	28%	30%
	140-159	12%	12%	12%	12%	16%	16%	16%	17%	16%	17%	18%	19%	21%	23%	24%	26%
	120-139	10%	10%	10%	11%	14%	14%	14%	15%	14%	15%	16%	17%	19%	20%	21%	23%
	100-119	10%	10%	11%	11%	14%	14%	15%	15%	14%	15%	16%	17%	18%	20%	21%	23%
60 - 64	160-179	16%	16%	16%	17%	21%	22%	22%	23%	22%	23%	25%	27%	28%	30%	33%	35%
	140-159	13%	14%	14%	15%	18%	19%	19%	20%	19%	20%	22%	23%	25%	26%	28%	31%
	120-139	12%	12%	12%	13%	16%	16%	17%	17%	16%	17%	19%	20%	21%	23%	25%	27%
	100-119	10%	10%	11%	11%	14%	14%	15%	15%	14%	15%	16%	17%	18%	20%	21%	23%
55 - 59	160-179	17%	18%	18%	19%	23%	24%	26%	27%	24%	26%	28%	30%	31%	34%	37%	40%
	140-159	15%	15%	16%	16%	20%	21%	22%	23%	21%	23%	24%	26%	27%	29%	32%	35%
	120-139	13%	13%	13%	14%	17%	18%	19%	20%	18%	19%	21%	22%	23%	25%	28%	30%
	100-119	11%	11%	12%	12%	15%	16%	16%	17%	15%	17%	18%	19%	20%	22%	24%	26%
50 - 54	160-179	19%	19%	20%	21%	26%	27%	28%	29%	26%	28%	31%	33%	34%	37%	40%	44%
	140-159	16%	16%	17%	18%	22%	23%	24%	25%	22%	24%	26%	29%	29%	32%	35%	38%
	120-139	13%	14%	14%	15%	19%	19%	20%	21%	19%	21%	23%	24%	25%	28%	30%	33%
	100-119	11%	12%	12%	13%	16%	17%	17%	18%	16%	18%	19%	21%	22%	24%	26%	28%
45 - 49	160-179	20%	20%	21%	22%	27%	29%	31%	32%	28%	30%	33%	36%	36%	39%	43%	47%
	140-159	17%	17%	18%	19%	23%	24%	26%	27%	24%	26%	28%	31%	31%	34%	37%	41%
	120-139	14%	15%	15%	16%	20%	21%	22%	23%	20%	22%	24%	26%	26%	29%	32%	35%
	100-119	12%	12%	13%	13%	17%	17%	18%	19%	17%	19%	20%	22%	23%	25%	27%	30%
40 - 44	160-179	20%	21%	22%	24%	29%	31%	33%	35%	29%	31%	34%	38%	37%	41%	45%	49%
	140-159	17%	18%	19%	20%	24%	26%	27%	29%	24%	27%	29%	32%	32%	35%	39%	43%
	120-139	14%	15%	16%	16%	20%	22%	23%	24%	21%	23%	25%	27%	27%	30%	33%	37%
	100-119	12%	13%	13%	14%	17%	18%	19%	20%	17%	19%	21%	23%	23%	26%	28%	31%

Non-HDL cholesterol (mmol/L)

150 200 250 mg/dL

LIFE-CVD2 model

Lifetime risk in the high risk region (risk from current age up to age 80 years)

Lifetime risk until 80	
Green	<20%
Orange	20 to <30%
Red	≥30%

Age	Systolic BP	Women, Non-smoking 3.0-3.9	Women, Non-smoking 4.0-4.9	Women, Non-smoking 5.0-5.9	Women, Non-smoking 6.0-6.9	Women, Smoking 3.0-3.9	Women, Smoking 4.0-4.9	Women, Smoking 5.0-5.9	Women, Smoking 6.0-6.9	Men, Non-smoking 3.0-3.9	Men, Non-smoking 4.0-4.9	Men, Non-smoking 5.0-5.9	Men, Non-smoking 6.0-6.9	Men, Smoking 3.0-3.9	Men, Smoking 4.0-4.9	Men, Smoking 5.0-5.9	Men, Smoking 6.0-6.9
75 - 79	160-179	7%	7%	7%	7%	10%	10%	10%	10%	8%	8%	9%	9%	11%	12%	12%	13%
	140-159	6%	6%	6%	6%	9%	9%	9%	9%	7%	7%	8%	8%	10%	10%	11%	11%
	120-139	5%	5%	5%	5%	8%	8%	8%	8%	6%	6%	7%	7%	8%	9%	9%	10%
	100-119	5%	5%	5%	5%	7%	7%	7%	7%	5%	5%	6%	6%	7%	8%	8%	8%
70 - 74	160-179	14%	14%	14%	14%	20%	20%	20%	21%	16%	17%	18%	19%	22%	23%	24%	26%
	140-159	12%	12%	12%	12%	17%	17%	18%	18%	14%	14%	15%	16%	19%	20%	21%	22%
	120-139	10%	10%	10%	11%	15%	15%	15%	15%	12%	12%	13%	14%	16%	17%	18%	19%
	100-119	9%	9%	9%	9%	13%	13%	13%	13%	10%	11%	11%	12%	14%	15%	16%	17%
65 - 69	160-179	17%	18%	18%	19%	25%	26%	26%	27%	21%	22%	23%	25%	27%	30%	32%	34%
	140-159	15%	15%	16%	16%	21%	22%	23%	23%	18%	19%	20%	21%	24%	25%	27%	29%
	120-139	13%	13%	13%	13%	18%	19%	19%	20%	15%	16%	17%	18%	20%	22%	23%	25%
	100-119	12%	12%	13%	13%	17%	18%	19%	19%	14%	15%	17%	18%	19%	21%	23%	25%
60 - 64	160-179	20%	20%	21%	22%	28%	29%	30%	32%	23%	25%	27%	29%	31%	34%	36%	39%
	140-159	17%	17%	18%	18%	24%	25%	26%	27%	20%	22%	23%	25%	27%	29%	31%	34%
	120-139	14%	15%	15%	15%	21%	21%	22%	23%	17%	18%	20%	21%	23%	25%	27%	29%
	100-119	12%	12%	13%	13%	17%	18%	19%	19%	14%	15%	17%	18%	19%	21%	23%	25%
55 - 59	160-179	22%	22%	23%	24%	31%	32%	34%	35%	26%	28%	30%	33%	34%	37%	40%	44%
	140-159	18%	19%	19%	20%	26%	27%	29%	30%	22%	24%	26%	28%	29%	32%	35%	38%
	120-139	15%	16%	16%	17%	22%	23%	24%	25%	18%	20%	22%	24%	24%	27%	29%	32%
	100-119	13%	13%	14%	14%	19%	19%	20%	21%	15%	17%	18%	20%	21%	23%	25%	27%
50 - 54	160-179	23%	24%	25%	26%	33%	35%	36%	38%	27%	30%	33%	36%	36%	39%	43%	48%
	140-159	19%	20%	20%	21%	28%	29%	31%	32%	23%	25%	28%	30%	30%	34%	37%	41%
	120-139	16%	16%	17%	18%	23%	24%	25%	27%	19%	21%	23%	25%	26%	29%	32%	35%
	100-119	13%	14%	14%	15%	19%	20%	21%	22%	16%	18%	19%	21%	22%	24%	27%	30%
45 - 49	160-179	24%	25%	26%	27%	35%	37%	39%	41%	29%	32%	35%	38%	38%	42%	46%	51%
	140-159	20%	20%	21%	22%	29%	31%	32%	34%	24%	27%	29%	32%	32%	36%	39%	44%
	120-139	16%	17%	18%	18%	24%	25%	27%	28%	20%	22%	24%	27%	27%	30%	33%	37%
	100-119	13%	14%	15%	15%	20%	21%	22%	23%	17%	18%	20%	22%	23%	25%	28%	31%
40 - 44	160-179	24%	26%	27%	28%	36%	38%	41%	43%	29%	33%	36%	40%	38%	43%	48%	53%
	140-159	20%	21%	22%	23%	30%	32%	34%	36%	25%	27%	30%	33%	33%	37%	41%	45%
	120-139	16%	17%	18%	19%	25%	26%	28%	29%	20%	23%	25%	28%	27%	31%	34%	38%
	100-119	14%	14%	15%	15%	20%	21%	23%	24%	17%	19%	21%	23%	23%	26%	29%	32%

Non-HDL cholesterol (mmol/L)

150 200 250
mg/dL

LIFE-CVD2 model

Lifetime risk in the very high risk region (risk from current age up to age 80 years)

Lifetime risk until 80	
Green	<20%
Orange	20 to <30%
Red	≥30%

Age	Systolic blood pressure	Women Non-smoking 3.0-3.9	4.0-4.9	5.0-5.9	6.0-6.9	Women Smoking 3.0-3.9	4.0-4.9	5.0-5.9	6.0-6.9	Men Non-smoking 3.0-3.9	4.0-4.9	5.0-5.9	6.0-6.9	Men Smoking 3.0-3.9	4.0-4.9	5.0-5.9	6.0-6.9
75 - 79	160-179	13%	13%	13%	13%	18%	18%	18%	18%	13%	13%	14%	14%	17%	18%	19%	20%
	140-159	11%	11%	11%	11%	16%	16%	16%	16%	11%	12%	12%	13%	15%	16%	17%	17%
	120-139	10%	10%	10%	10%	14%	14%	14%	14%	10%	10%	11%	11%	14%	14%	15%	15%
	100-119	9%	9%	9%	9%	13%	13%	13%	13%	9%	9%	9%	10%	12%	12%	13%	14%
70 - 74	160-179	24%	25%	25%	25%	34%	34%	34%	35%	25%	26%	27%	29%	33%	34%	36%	38%
	140-159	21%	22%	22%	22%	30%	30%	30%	31%	22%	23%	24%	25%	29%	30%	32%	34%
	120-139	19%	19%	19%	19%	26%	26%	27%	27%	19%	20%	21%	22%	25%	27%	28%	30%
	100-119	16%	16%	17%	17%	23%	23%	24%	24%	17%	17%	18%	19%	22%	23%	25%	26%
65 - 69	160-179	30%	31%	31%	32%	41%	42%	43%	44%	32%	34%	36%	38%	41%	43%	46%	48%
	140-159	26%	27%	27%	28%	36%	37%	38%	39%	28%	29%	31%	33%	36%	38%	41%	43%
	120-139	23%	23%	24%	24%	32%	33%	33%	34%	24%	26%	27%	29%	32%	34%	36%	38%
	100-119	22%	22%	23%	24%	31%	32%	33%	33%	23%	25%	27%	29%	30%	33%	35%	38%
60 - 64	160-179	34%	35%	36%	37%	46%	47%	49%	50%	36%	38%	41%	43%	45%	48%	51%	55%
	140-159	30%	30%	31%	32%	40%	42%	43%	44%	31%	33%	36%	38%	40%	43%	46%	49%
	120-139	26%	26%	27%	27%	35%	36%	37%	39%	27%	29%	31%	33%	35%	38%	40%	43%
	100-119	22%	22%	23%	24%	31%	32%	33%	33%	23%	25%	27%	29%	30%	33%	35%	38%
55 - 59	160-179	37%	38%	39%	40%	49%	51%	53%	55%	39%	42%	45%	48%	48%	52%	56%	59%
	140-159	32%	33%	34%	35%	43%	45%	46%	48%	34%	37%	39%	42%	43%	46%	50%	53%
	120-139	27%	28%	29%	30%	38%	39%	40%	42%	29%	32%	34%	37%	37%	41%	44%	47%
	100-119	23%	24%	25%	25%	32%	34%	35%	36%	25%	27%	29%	32%	33%	35%	38%	41%
50 - 54	160-179	38%	40%	41%	43%	52%	54%	56%	58%	41%	45%	48%	52%	51%	55%	59%	63%
	140-159	33%	34%	35%	37%	45%	47%	49%	51%	36%	39%	42%	45%	45%	49%	53%	57%
	120-139	28%	29%	30%	31%	39%	41%	42%	44%	31%	33%	36%	39%	39%	43%	46%	50%
	100-119	24%	25%	26%	26%	34%	35%	36%	38%	26%	29%	31%	34%	34%	37%	41%	44%
45 - 49	160-179	40%	41%	43%	45%	54%	56%	59%	61%	43%	47%	51%	54%	53%	57%	62%	66%
	140-159	34%	35%	37%	38%	47%	49%	51%	53%	37%	41%	44%	48%	47%	51%	55%	60%
	120-139	29%	30%	31%	32%	40%	42%	44%	46%	32%	35%	38%	41%	41%	45%	49%	53%
	100-119	24%	25%	26%	27%	34%	36%	38%	39%	27%	30%	32%	35%	35%	39%	42%	46%
40 - 44	160-179	41%	43%	44%	47%	56%	58%	61%	64%	44%	48%	52%	56%	54%	59%	63%	68%
	140-159	35%	36%	38%	39%	48%	50%	53%	55%	38%	41%	45%	49%	47%	52%	57%	61%
	120-139	29%	30%	32%	33%	41%	43%	45%	47%	33%	36%	39%	42%	41%	45%	50%	54%
	100-119	25%	26%	27%	28%	35%	37%	38%	40%	28%	30%	33%	36%	36%	39%	43%	47%

Non-HDL cholesterol (mmol/L)

150 200 250
mg/dL

LIFE-CVD2 model

CVD-free lifetime gain (in years) from lifelong SBP reduction to <140 mmHg in the low risk region

- < 0.5 years
- 0.5 - 0.9 years
- 1.0 - 1.4 years
- 1.5 - 2.0 years
- ≥ 2.0 years

Age	SBP	Women								Men							
		Non-smoking				Smoking				Non-smoking				Smoking			
		3.0-3.9	4.0-4.9	5.0-5.9	6.0-6.9	3.0-3.9	4.0-4.9	5.0-5.9	6.0-6.9	3.0-3.9	4.0-4.9	5.0-5.9	6.0-6.9	3.0-3.9	4.0-4.9	5.0-5.9	6.0-6.9
85 - 89	160-179	1.2	1.4	1.6	1.8	0.5	0.6	0.8	0.9	1.2	1.3	1.4	1.6	0.8	0.9	1.0	1.1
	140-159	0.5	0.6	0.7	0.8	0.2	0.3	0.3	0.4	0.5	0.5	0.5	0.6	0.3	0.3	0.4	0.4
	120-139	-	-	-	-	-	-	-	-	-	-	-	-	-	-	-	-
	100-119	-	-	-	-	-	-	-	-	-	-	-	-	-	-	-	-
80 - 84	160-179	1.5	1.7	2.0	2.2	0.8	0.9	1.1	1.2	1.5	1.6	1.8	2.0	1.1	1.2	1.3	1.5
	140-159	0.7	0.8	0.9	1.0	0.3	0.4	0.5	0.6	0.6	0.7	0.7	0.8	0.4	0.5	0.5	0.6
	120-139	-	-	-	-	-	-	-	-	-	-	-	-	-	-	-	-
	100-119	-	-	-	-	-	-	-	-	-	-	-	-	-	-	-	-
75 - 79	160-179	1.8	2.0	2.3	2.6	1.0	1.2	1.4	1.6	1.8	2.0	2.2	2.4	1.4	1.5	1.7	1.9
	140-159	0.8	0.9	1.0	1.1	0.5	0.5	0.6	0.7	0.7	0.8	0.9	1.0	0.6	0.6	0.7	0.8
	120-139	-	-	-	-	-	-	-	-	-	-	-	-	-	-	-	-
	100-119	-	-	-	-	-	-	-	-	-	-	-	-	-	-	-	-
70 - 74	160-179	2.0	2.3	2.5	2.9	1.3	1.5	1.7	2.0	2.1	2.3	2.5	2.8	1.7	1.9	2.2	2.4
	140-159	0.9	1.0	1.1	1.2	0.6	0.7	0.8	0.9	0.9	0.9	1.0	1.1	0.7	0.8	0.9	1.0
	120-139	-	-	-	-	-	-	-	-	-	-	-	-	-	-	-	-
	100-119	-	-	-	-	-	-	-	-	-	-	-	-	-	-	-	-
65 - 69	160-179	2.2	2.5	2.8	3.1	1.6	1.8	2.1	2.3	2.4	2.6	2.9	3.2	2.1	2.4	2.7	3.0
	140-159	1.0	1.1	1.2	1.4	0.7	0.8	0.9	1.0	1.0	1.1	1.2	1.3	0.9	1.0	1.1	1.3
	120-139	-	-	-	-	-	-	-	-	-	-	-	-	-	-	-	-
	100-119	-	-	-	-	-	-	-	-	-	-	-	-	-	-	-	-
60 - 64	160-179	2.4	2.7	3.0	3.3	1.9	2.1	2.4	2.7	2.6	2.9	3.2	3.6	2.6	2.9	3.3	3.7
	140-159	1.1	1.2	1.3	1.4	0.8	0.9	1.0	1.2	1.1	1.2	1.3	1.5	1.1	1.2	1.4	1.5
	120-139	-	-	-	-	-	-	-	-	-	-	-	-	-	-	-	-
	100-119	-	-	-	-	-	-	-	-	-	-	-	-	-	-	-	-
55 - 59	160-179	2.6	2.9	3.2	3.5	2.2	2.5	2.8	3.1	2.9	3.2	3.6	4.0	3.0	3.4	3.9	4.4
	140-159	1.1	1.2	1.4	1.5	0.9	1.0	1.2	1.3	1.2	1.3	1.5	1.6	1.2	1.4	1.6	1.8
	120-139	-	-	-	-	-	-	-	-	-	-	-	-	-	-	-	-
	100-119	-	-	-	-	-	-	-	-	-	-	-	-	-	-	-	-
50 - 54	160-179	2.7	3.0	3.4	3.7	2.5	2.8	3.2	3.6	3.1	3.5	3.9	4.4	3.5	4.0	4.6	5.2
	140-159	1.2	1.3	1.4	1.6	1.0	1.2	1.3	1.5	1.3	1.4	1.6	1.8	1.4	1.6	1.9	2.1
	120-139	-	-	-	-	-	-	-	-	-	-	-	-	-	-	-	-
	100-119	-	-	-	-	-	-	-	-	-	-	-	-	-	-	-	-
45 - 49	160-179	2.8	3.1	3.5	3.9	2.8	3.2	3.6	4.1	3.3	3.7	4.2	4.8	3.9	4.5	5.2	6.0
	140-159	1.2	1.3	1.5	1.7	1.1	1.3	1.5	1.7	1.4	1.5	1.7	1.9	1.6	1.8	2.1	2.4
	120-139	-	-	-	-	-	-	-	-	-	-	-	-	-	-	-	-
	100-119	-	-	-	-	-	-	-	-	-	-	-	-	-	-	-	-
40 - 44	160-179	2.9	3.2	3.6	4.1	3.0	3.5	4.0	4.6	3.5	3.9	4.4	5.0	4.2	4.9	5.7	6.7
	140-159	1.2	1.4	1.5	1.7	1.2	1.4	1.6	1.8	1.4	1.6	1.8	2.0	1.7	2.0	2.3	2.7
	120-139	-	-	-	-	-	-	-	-	-	-	-	-	-	-	-	-
	100-119	-	-	-	-	-	-	-	-	-	-	-	-	-	-	-	-

Non-HDL cholesterol (mmol/L)

150 200 250 mg/dL

LIFE-CVD2 model

CVD-free lifetime gain (in years) from lifelong SBP reduction to <140 mmHg in the moderate risk region

Colour key
< 0.5 years
0.5 - 0.9 years
1.0 - 1.4 years
1.5 - 2.0 years
≥ 2.0 years

Women

Age	SBP	Non-smoking 3.0-3.9	Non-smoking 4.0-4.9	Non-smoking 5.0-5.9	Non-smoking 6.0-6.9	Smoking 3.0-3.9	Smoking 4.0-4.9	Smoking 5.0-5.9	Smoking 6.0-6.9
85 - 89	160-179	1.4	1.6	1.9	2.1	0.6	0.8	0.9	1.1
	140-159	0.6	0.7	0.8	0.9	0.3	0.3	0.4	0.5
	120-139	-	-	-	-	-	-	-	-
	100-119	-	-	-	-	-	-	-	-
80 - 84	160-179	1.8	2.0	2.3	2.6	1.0	1.1	1.3	1.5
	140-159	0.8	0.9	1.0	1.1	0.4	0.5	0.6	0.7
	120-139	-	-	-	-	-	-	-	-
	100-119	-	-	-	-	-	-	-	-
75 - 79	160-179	2.2	2.4	2.7	3.0	1.3	1.5	1.7	2.0
	140-159	1.0	1.1	1.2	1.3	0.6	0.7	0.8	0.9
	120-139	-	-	-	-	-	-	-	-
	100-119	-	-	-	-	-	-	-	-
70 - 74	160-179	2.5	2.7	3.0	3.4	1.6	1.9	2.1	2.4
	140-159	1.1	1.2	1.3	1.5	0.7	0.8	0.9	1.0
	120-139	-	-	-	-	-	-	-	-
	100-119	-	-	-	-	-	-	-	-
65 - 69	160-179	2.7	3.0	3.3	3.7	2.0	2.2	2.5	2.8
	140-159	1.2	1.3	1.4	1.6	0.9	1.0	1.1	1.2
	120-139	-	-	-	-	-	-	-	-
	100-119	-	-	-	-	-	-	-	-
60 - 64	160-179	2.9	3.2	3.6	4.0	2.3	2.6	3.0	3.3
	140-159	1.3	1.4	1.5	1.7	1.0	1.1	1.3	1.4
	120-139	-	-	-	-	-	-	-	-
	100-119	-	-	-	-	-	-	-	-
55 - 59	160-179	3.1	3.4	3.8	4.2	2.7	3.1	3.4	3.9
	140-159	1.3	1.5	1.6	1.8	1.2	1.3	1.5	1.6
	120-139	-	-	-	-	-	-	-	-
	100-119	-	-	-	-	-	-	-	-
50 - 54	160-179	3.2	3.6	4.0	4.5	3.1	3.5	4.0	4.5
	140-159	1.4	1.5	1.7	1.9	1.3	1.5	1.6	1.8
	120-139	-	-	-	-	-	-	-	-
	100-119	-	-	-	-	-	-	-	-
45 - 49	160-179	3.4	3.8	4.2	4.7	3.5	4.0	4.5	5.1
	140-159	1.4	1.6	1.8	2.0	1.4	1.6	1.8	2.1
	120-139	-	-	-	-	-	-	-	-
	100-119	-	-	-	-	-	-	-	-
40 - 44	160-179	3.5	3.9	4.4	4.9	3.8	4.4	5.0	5.8
	140-159	1.5	1.6	1.8	2.0	1.5	1.8	2.0	2.3
	120-139	-	-	-	-	-	-	-	-
	100-119	-	-	-	-	-	-	-	-

Men

Age	SBP	Non-smoking 3.0-3.9	Non-smoking 4.0-4.9	Non-smoking 5.0-5.9	Non-smoking 6.0-6.9	Smoking 3.0-3.9	Smoking 4.0-4.9	Smoking 5.0-5.9	Smoking 6.0-6.9
85 - 89	160-179	1.4	1.6	1.7	1.9	1.0	1.1	1.3	1.4
	140-159	0.5	0.6	0.7	0.7	0.4	0.4	0.5	0.5
	120-139	-	-	-	-	-	-	-	-
	100-119	-	-	-	-	-	-	-	-
80 - 84	160-179	1.8	2.0	2.1	2.3	1.3	1.5	1.6	1.8
	140-159	0.7	0.8	0.9	0.9	0.5	0.6	0.6	0.7
	120-139	-	-	-	-	-	-	-	-
	100-119	-	-	-	-	-	-	-	-
75 - 79	160-179	2.2	2.4	2.6	2.8	1.7	1.9	2.1	2.3
	140-159	0.9	1.0	1.0	1.1	0.7	0.8	0.8	0.9
	120-139	-	-	-	-	-	-	-	-
	100-119	-	-	-	-	-	-	-	-
70 - 74	160-179	2.5	2.8	3.0	3.3	2.2	2.4	2.7	2.9
	140-159	1.0	1.1	1.2	1.3	0.9	1.0	1.1	1.2
	120-139	-	-	-	-	-	-	-	-
	100-119	-	-	-	-	-	-	-	-
65 - 69	160-179	2.9	3.2	3.5	3.8	2.7	3.0	3.3	3.7
	140-159	1.2	1.3	1.4	1.6	1.1	1.2	1.4	1.5
	120-139	-	-	-	-	-	-	-	-
	100-119	-	-	-	-	-	-	-	-
60 - 64	160-179	3.3	3.6	4.0	4.4	3.2	3.6	4.0	4.5
	140-159	1.4	1.5	1.6	1.8	1.3	1.5	1.7	1.9
	120-139	-	-	-	-	-	-	-	-
	100-119	-	-	-	-	-	-	-	-
55 - 59	160-179	3.6	4.0	4.5	4.9	3.8	4.2	4.8	5.4
	140-159	1.5	1.7	1.8	2.0	1.6	1.8	2.0	2.2
	120-139	-	-	-	-	-	-	-	-
	100-119	-	-	-	-	-	-	-	-
50 - 54	160-179	4.0	4.4	4.9	5.5	4.3	4.9	5.6	6.3
	140-159	1.6	1.8	2.0	2.3	1.8	2.0	2.3	2.6
	120-139	-	-	-	-	-	-	-	-
	100-119	-	-	-	-	-	-	-	-
45 - 49	160-179	4.2	4.8	5.4	6.1	4.8	5.5	6.4	7.3
	140-159	1.7	1.9	2.2	2.4	2.0	2.3	2.6	3.0
	120-139	-	-	-	-	-	-	-	-
	100-119	-	-	-	-	-	-	-	-
40 - 44	160-179	4.4	5.0	5.7	6.5	5.2	6.0	7.0	8.1
	140-159	1.8	2.0	2.3	2.6	2.1	2.4	2.8	3.3
	120-139	-	-	-	-	-	-	-	-
	100-119	-	-	-	-	-	-	-	-

Non-HDL cholesterol (mmol/L)

150 200 250 mg/dL

LIFE-CVD2 model

CVD-free lifetime gain (in years) from lifelong SBP reduction to <140 mmHg in the high risk region

Legend
< 0.5 years
0.5 - 0.9 years
1.0 - 1.4 years
1.5 - 2.0 years
≥ 2.0 years

Age		Women								Men							
		Non-smoking				Smoking				Non-smoking				Smoking			
		3.0-3.9	4.0-4.9	5.0-5.9	6.0-6.9	3.0-3.9	4.0-4.9	5.0-5.9	6.0-6.9	3.0-3.9	4.0-4.9	5.0-5.9	6.0-6.9	3.0-3.9	4.0-4.9	5.0-5.9	6.0-6.9
85 - 89	160-179	1.8	2.0	2.3	2.5	1.1	1.2	1.4	1.6	1.4	1.5	1.6	1.7	1.0	1.1	1.2	1.3
	140-159	0.7	0.8	0.9	1.0	0.5	0.5	0.6	0.7	0.5	0.6	0.6	0.7	0.4	0.4	0.5	0.5
	120-139	-	-	-	-	-	-	-	-	-	-	-	-	-	-	-	-
	100-119	-	-	-	-	-	-	-	-	-	-	-	-	-	-	-	-
80 - 84	160-179	2.3	2.5	2.8	3.0	1.5	1.7	1.9	2.1	1.7	1.9	2.0	2.2	1.3	1.4	1.5	1.7
	140-159	1.0	1.1	1.2	1.3	0.6	0.7	0.8	0.9	0.7	0.7	0.8	0.9	0.5	0.6	0.6	0.7
	120-139	-	-	-	-	-	-	-	-	-	-	-	-	-	-	-	-
	100-119	-	-	-	-	-	-	-	-	-	-	-	-	-	-	-	-
75 - 79	160-179	2.7	3.0	3.2	3.5	1.9	2.1	2.3	2.6	2.1	2.2	2.4	2.6	1.7	1.8	2.0	2.2
	140-159	1.2	1.3	1.4	1.5	0.8	0.9	1.0	1.1	0.8	0.9	1.0	1.1	0.7	0.7	0.8	0.9
	120-139	-	-	-	-	-	-	-	-	-	-	-	-	-	-	-	-
	100-119	-	-	-	-	-	-	-	-	-	-	-	-	-	-	-	-
70 - 74	160-179	3.0	3.3	3.6	3.9	2.3	2.5	2.8	3.1	2.4	2.7	2.9	3.1	2.1	2.3	2.6	2.8
	140-159	1.3	1.4	1.5	1.6	1.0	1.1	1.2	1.3	1.0	1.1	1.2	1.3	0.9	1.0	1.1	1.2
	120-139	-	-	-	-	-	-	-	-	-	-	-	-	-	-	-	-
	100-119	-	-	-	-	-	-	-	-	-	-	-	-	-	-	-	-
65 - 69	160-179	3.3	3.6	3.9	4.2	2.7	2.9	3.3	3.6	2.8	3.1	3.4	3.7	2.6	2.9	3.2	3.6
	140-159	1.4	1.5	1.6	1.8	1.1	1.2	1.4	1.5	1.2	1.3	1.4	1.5	1.1	1.2	1.3	1.5
	120-139	-	-	-	-	-	-	-	-	-	-	-	-	-	-	-	-
	100-119	-	-	-	-	-	-	-	-	-	-	-	-	-	-	-	-
60 - 64	160-179	3.5	3.8	4.1	4.5	3.1	3.4	3.8	4.2	3.2	3.5	3.9	4.3	3.0	3.4	3.9	4.3
	140-159	1.5	1.6	1.7	1.9	1.3	1.4	1.6	1.7	1.3	1.5	1.6	1.8	1.3	1.4	1.6	1.8
	120-139	-	-	-	-	-	-	-	-	-	-	-	-	-	-	-	-
	100-119	-	-	-	-	-	-	-	-	-	-	-	-	-	-	-	-
55 - 59	160-179	3.7	4.0	4.4	4.8	3.5	3.9	4.4	4.8	3.5	3.9	4.4	4.9	3.5	4.0	4.6	5.1
	140-159	1.5	1.7	1.8	2.0	1.5	1.6	1.8	2.0	1.4	1.6	1.8	2.0	1.4	1.6	1.9	2.1
	120-139	-	-	-	-	-	-	-	-	-	-	-	-	-	-	-	-
	100-119	-	-	-	-	-	-	-	-	-	-	-	-	-	-	-	-
50 - 54	160-179	3.8	4.2	4.6	5.0	4.0	4.4	5.0	5.6	3.8	4.3	4.9	5.5	4.0	4.6	5.3	6.0
	140-159	1.6	1.7	1.9	2.0	1.6	1.8	2.0	2.2	1.5	1.7	1.9	2.2	1.6	1.9	2.2	2.5
	120-139	-	-	-	-	-	-	-	-	-	-	-	-	-	-	-	-
	100-119	-	-	-	-	-	-	-	-	-	-	-	-	-	-	-	-
45 - 49	160-179	3.9	4.3	4.8	5.2	4.4	5.0	5.6	6.4	4.1	4.6	5.3	6.0	4.5	5.2	6.0	6.9
	140-159	1.6	1.8	1.9	2.1	1.7	2.0	2.2	2.5	1.6	1.8	2.1	2.4	1.8	2.1	2.4	2.8
	120-139	-	-	-	-	-	-	-	-	-	-	-	-	-	-	-	-
	100-119	-	-	-	-	-	-	-	-	-	-	-	-	-	-	-	-
40 - 44	160-179	4.0	4.4	4.9	5.4	4.8	5.5	6.3	7.2	4.2	4.8	5.5	6.4	4.8	5.6	6.6	7.7
	140-159	1.6	1.8	2.0	2.2	1.9	2.1	2.4	2.7	1.7	1.9	2.2	2.5	1.9	2.3	2.6	3.1
	120-139	-	-	-	-	-	-	-	-	-	-	-	-	-	-	-	-
	100-119	-	-	-	-	-	-	-	-	-	-	-	-	-	-	-	-

Non-HDL cholesterol (mmol/L)

150 200 250
mg/dL

LIFE-CVD2 model

CVD-free lifetime gain (in years) from lifelong SBP reduction to <140 mmHg in the very high risk region

Shade	Range
lightest	< 0.5 years
light	0.5 - 0.9 years
medium	1.0 - 1.4 years
dark	1.5 - 2.0 years
darkest	≥ 2.0 years

Age	SBP	Women Non-smoking 3.0-3.9	4.0-4.9	5.0-5.9	6.0-6.9	Women Smoking 3.0-3.9	4.0-4.9	5.0-5.9	6.0-6.9	Men Non-smoking 3.0-3.9	4.0-4.9	5.0-5.9	6.0-6.9	Men Smoking 3.0-3.9	4.0-4.9	5.0-5.9	6.0-6.9
85 - 89	160-179	2.1	2.2	2.4	2.6	1.5	1.6	1.8	2.0	1.7	1.8	1.9	2.0	1.3	1.4	1.5	1.6
	140-159	0.8	0.9	0.9	1.0	0.6	0.7	0.7	0.8	0.6	0.7	0.7	0.8	0.5	0.5	0.6	0.6
	120-139	-	-	-	-	-	-	-	-	-	-	-	-	-	-	-	-
	100-119	-	-	-	-	-	-	-	-	-	-	-	-	-	-	-	-
80 - 84	160-179	2.7	2.9	3.1	3.3	2.0	2.2	2.4	2.5	2.1	2.2	2.4	2.5	1.7	1.8	1.9	2.0
	140-159	1.1	1.2	1.2	1.3	0.8	0.9	1.0	1.0	0.8	0.9	0.9	1.0	0.7	0.7	0.7	0.8
	120-139	-	-	-	-	-	-	-	-	-	-	-	-	-	-	-	-
	100-119	-	-	-	-	-	-	-	-	-	-	-	-	-	-	-	-
75 - 79	160-179	3.3	3.5	3.7	3.9	2.6	2.8	3.0	3.2	2.6	2.8	2.9	3.1	2.2	2.4	2.5	2.7
	140-159	1.4	1.4	1.5	1.6	1.1	1.2	1.2	1.3	1.1	1.1	1.2	1.3	0.9	1.0	1.0	1.1
	120-139	-	-	-	-	-	-	-	-	-	-	-	-	-	-	-	-
	100-119	-	-	-	-	-	-	-	-	-	-	-	-	-	-	-	-
70 - 74	160-179	3.8	4.0	4.3	4.5	3.2	3.4	3.6	3.9	3.3	3.5	3.7	3.9	2.8	3.0	3.2	3.4
	140-159	1.6	1.7	1.8	1.9	1.3	1.4	1.5	1.6	1.4	1.4	1.5	1.6	1.2	1.2	1.3	1.4
	120-139	-	-	-	-	-	-	-	-	-	-	-	-	-	-	-	-
	100-119	-	-	-	-	-	-	-	-	-	-	-	-	-	-	-	-
65 - 69	160-179	4.3	4.5	4.8	5.1	3.8	4.1	4.3	4.6	3.9	4.2	4.5	4.8	3.4	3.7	4.0	4.2
	140-159	1.8	1.9	2.0	2.1	1.6	1.7	1.8	1.9	1.6	1.7	1.9	2.0	1.4	1.5	1.7	1.8
	120-139	-	-	-	-	-	-	-	-	-	-	-	-	-	-	-	-
	100-119	-	-	-	-	-	-	-	-	-	-	-	-	-	-	-	-
60 - 64	160-179	4.7	5.0	5.3	5.6	4.5	4.8	5.1	5.5	4.4	4.8	5.2	5.6	4.0	4.3	4.7	5.0
	140-159	1.9	2.1	2.2	2.3	1.9	2.0	2.1	2.3	1.8	2.0	2.2	2.3	1.7	1.8	2.0	2.1
	120-139	-	-	-	-	-	-	-	-	0	0	0	0	0	0	0	0
	100-119	-	-	-	-	-	-	-	-	-	-	-	-	-	-	-	-
55 - 59	160-179	5.0	5.4	5.8	6.2	5.2	5.6	6.1	6.5	5.0	5.4	5.9	6.5	4.6	5.1	5.5	5.9
	140-159	2.1	2.2	2.4	2.5	2.2	2.3	2.5	2.7	2.1	2.2	2.4	2.7	1.9	2.1	2.3	2.5
	120-139	-	-	-	-	-	-	-	-	-	-	-	-	-	-	-	-
	100-119	-	-	-	-	-	-	-	-	-	-	-	-	-	-	-	-
50 - 54	160-179	5.4	5.8	6.2	6.7	6.0	6.5	7.1	7.6	5.5	6.1	6.7	7.4	5.3	5.8	6.4	6.9
	140-159	2.2	2.3	2.5	2.7	2.5	2.7	2.9	3.1	2.2	2.5	2.7	3.0	2.2	2.4	2.7	2.9
	120-139	-	-	-	-	-	-	-	-	-	-	-	-	-	-	-	-
	100-119	-	-	-	-	-	-	-	-	-	-	-	-	-	-	-	-
45 - 49	160-179	5.7	6.2	6.7	7.3	6.8	7.4	8.1	8.8	5.9	6.6	7.4	8.2	5.9	6.6	7.3	8.0
	140-159	2.3	2.5	2.7	2.9	2.7	3.0	3.3	3.6	2.4	2.7	3.0	3.3	2.5	2.8	3.0	3.3
	120-139	-	-	-	-	-	-	-	-	-	-	-	-	-	-	-	-
	100-119	-	-	-	-	-	-	-	-	-	-	-	-	-	-	-	-
40 - 44	160-179	5.9	6.5	7.1	7.8	7.5	8.2	9.1	9.9	6.3	7.0	7.9	8.9	6.4	7.2	8.1	8.8
	140-159	2.4	2.6	2.8	3.0	3.0	3.3	3.6	4.0	2.5	2.8	3.2	3.6	2.7	3.0	3.3	3.7
	120-139	-	-	-	-	-	-	-	-	-	-	-	-	-	-	-	-
	100-119	-	-	-	-	-	-	-	-	-	-	-	-	-	-	-	-

Non-HDL cholesterol (mmol/L)

150 200 250
mg/dL

LIFE-CVD2 model

CVD-free lifetime gain (in years) from lifelong 40% LDL cholesterol reduction in the low risk region

Legend
< 0.5 years
0.5 - 0.9 years
1.0 - 1.4 years
1.5 - 2.0 years
≥ 2.0 years

Age		Women								Men							
		Non-smoking				Smoking				Non-smoking				Smoking			
	Non-HDL cholesterol (mmol/L)	3.0-3.9	4.0-4.9	5.0-5.9	6.0-6.9	3.0-3.9	4.0-4.9	5.0-5.9	6.0-6.9	3.0-3.9	4.0-4.9	5.0-5.9	6.0-6.9	3.0-3.9	4.0-4.9	5.0-5.9	6.0-6.9
85 - 89	160-179	0.5	0.7	1.0	1.4	0.2	0.3	0.5	0.7	0.5	0.7	0.9	1.2	0.3	0.5	0.7	0.9
	140-159	0.4	0.6	0.9	1.3	0.2	0.3	0.4	0.6	0.4	0.5	0.7	1.0	0.3	0.4	0.5	0.7
	120-139	0.4	0.6	0.9	1.2	0.2	0.3	0.4	0.6	0.3	0.4	0.6	0.8	0.2	0.3	0.4	0.6
	100-119	0.3	0.5	0.8	1.1	0.2	0.2	0.4	0.5	0.2	0.3	0.5	0.6	0.2	0.3	0.3	0.4
80 - 84	160-179	0.6	0.9	1.3	1.7	0.3	0.5	0.7	1.0	0.6	0.8	1.2	1.5	0.4	0.6	0.9	1.1
	140-159	0.5	0.8	1.2	1.6	0.3	0.4	0.6	0.9	0.5	0.7	1.0	1.3	0.3	0.5	0.7	0.9
	120-139	0.5	0.7	1.1	1.4	0.3	0.4	0.6	0.8	0.4	0.6	0.8	1.0	0.3	0.4	0.6	0.8
	100-119	0.4	0.7	1.0	1.3	0.2	0.4	0.5	0.7	0.3	0.5	0.7	0.9	0.2	0.3	0.5	0.6
75 - 79	160-179	0.7	1.1	1.5	2.0	0.4	0.7	0.9	1.3	0.7	1.0	1.4	1.8	0.5	0.8	1.1	1.4
	140-159	0.7	1.0	1.3	1.8	0.4	0.6	0.8	1.1	0.6	0.9	1.2	1.5	0.5	0.7	0.9	1.2
	120-139	0.6	0.9	1.2	1.6	0.3	0.5	0.8	1.0	0.5	0.7	1.0	1.3	0.4	0.6	0.8	1.0
	100-119	0.5	0.8	1.1	1.5	0.3	0.5	0.7	0.9	0.4	0.6	0.8	1.1	0.3	0.5	0.6	0.9
70 - 74	160-179	0.8	1.2	1.7	2.2	0.5	0.8	1.1	1.5	0.8	1.2	1.6	2.1	0.7	1.0	1.4	1.9
	140-159	0.7	1.1	1.5	2.0	0.5	0.7	1.0	1.4	0.7	1.0	1.4	1.8	0.6	0.9	1.2	1.6
	120-139	0.7	1.0	1.4	1.8	0.4	0.6	0.9	1.2	0.6	0.9	1.2	1.5	0.5	0.7	1.0	1.3
	100-119	0.6	0.9	1.2	1.6	0.4	0.6	0.8	1.1	0.5	0.7	1.0	1.3	0.4	0.6	0.8	1.1
65 - 69	160-179	0.9	1.3	1.8	2.4	0.7	1.0	1.4	1.8	0.9	1.4	1.9	2.4	0.9	1.3	1.8	2.3
	140-159	0.8	1.2	1.6	2.1	0.6	0.9	1.2	1.6	0.8	1.2	1.6	2.0	0.7	1.1	1.5	2.0
	120-139	0.7	1.1	1.5	1.9	0.5	0.8	1.1	1.4	0.7	1.0	1.3	1.7	0.6	0.9	1.3	1.7
	100-119	0.7	1.0	1.4	1.8	0.5	0.7	1.0	1.4	0.6	0.9	1.2	1.6	0.6	0.9	1.2	1.6
60 - 64	160-179	1.0	1.4	2.0	2.6	0.8	1.2	1.6	2.1	1.1	1.6	2.1	2.7	1.0	1.6	2.2	2.9
	140-159	0.9	1.3	1.7	2.3	0.7	1.0	1.4	1.8	0.9	1.3	1.8	2.3	0.9	1.3	1.8	2.4
	120-139	0.8	1.1	1.5	2.0	0.6	0.9	1.2	1.6	0.7	1.1	1.5	1.9	0.7	1.1	1.5	2.0
	100-119	0.7	1.0	1.4	1.8	0.5	0.7	1.0	1.4	0.6	0.9	1.2	1.6	0.6	0.9	1.2	1.6
55 - 59	160-179	1.0	1.6	2.1	2.8	0.9	1.4	1.9	2.5	1.2	1.7	2.4	3.1	1.2	1.9	2.6	3.4
	140-159	0.9	1.3	1.8	2.4	0.8	1.1	1.6	2.1	1.0	1.4	1.9	2.5	1.0	1.5	2.1	2.8
	120-139	0.8	1.2	1.6	2.1	0.6	1.0	1.3	1.8	0.8	1.2	1.6	2.1	0.8	1.3	1.7	2.3
	100-119	0.7	1.0	1.4	1.9	0.6	0.8	1.1	1.5	0.7	1.0	1.3	1.7	0.7	1.0	1.4	1.9
50 - 54	160-179	1.1	1.6	2.2	2.9	1.0	1.6	2.1	2.8	1.3	1.9	2.6	3.4	1.4	2.1	3.0	4.1
	140-159	0.9	1.4	1.9	2.5	0.9	1.3	1.8	2.3	1.0	1.5	2.1	2.7	1.2	1.8	2.5	3.3
	120-139	0.8	1.2	1.7	2.2	0.7	1.1	1.5	1.9	0.9	1.2	1.7	2.2	0.9	1.4	2.0	2.7
	100-119	0.7	1.1	1.5	1.9	0.6	0.9	1.2	1.6	0.7	1.0	1.4	1.8	0.8	1.1	1.6	2.1
45 - 49	160-179	1.1	1.7	2.3	3.1	1.2	1.8	2.4	3.2	1.4	2.1	2.8	3.7	1.6	2.4	3.5	4.7
	140-159	1.0	1.5	2.0	2.6	0.9	1.4	2.0	2.6	1.1	1.6	2.2	2.9	1.3	2.0	2.8	3.8
	120-139	0.8	1.3	1.7	2.2	0.8	1.1	1.6	2.1	0.9	1.3	1.8	2.3	1.0	1.6	2.2	3.0
	100-119	0.7	1.1	1.5	1.9	0.6	0.9	1.3	1.7	0.7	1.0	1.4	1.9	0.8	1.2	1.7	2.3
40 - 44	160-179	1.2	1.8	2.4	3.2	1.3	2.0	2.7	3.6	1.5	2.2	3.0	4.0	1.7	2.7	3.8	5.2
	140-159	1.0	1.5	2.0	2.7	1.0	1.5	2.1	2.8	1.1	1.7	2.4	3.1	1.4	2.1	3.0	4.1
	120-139	0.9	1.3	1.8	2.3	0.8	1.2	1.7	2.2	0.9	1.3	1.9	2.4	1.1	1.7	2.4	3.2
	100-119	0.7	1.1	1.5	2.0	0.7	1.0	1.4	1.8	0.7	1.1	1.5	1.9	0.9	1.3	1.8	2.5

Non-HDL cholesterol (mmol/L)

150 200 250 mg/dL

LIFE-CVD2 model

CVD-free lifetime gain (in years) from lifelong 40% LDL cholesterol reduction in the moderate risk region

Legend
< 0.5 years
0.5 - 0.9 years
1.0 - 1.4 years
1.5 - 2.0 years
≥ 2.0 years

		Women								Men							
		Non-smoking				Smoking				Non-smoking				Smoking			
Age		3.0-3.9	4.0-4.9	5.0-5.9	6.0-6.9	3.0-3.9	4.0-4.9	5.0-5.9	6.0-6.9	3.0-3.9	4.0-4.9	5.0-5.9	6.0-6.9	3.0-3.9	4.0-4.9	5.0-5.9	6.0-6.9
85 - 89	160-179	0.5	0.8	1.2	1.6	0.3	0.4	0.6	0.8	0.5	0.8	1.1	1.4	0.4	0.6	0.8	1.0
	140-159	0.5	0.8	1.1	1.5	0.2	0.4	0.5	0.8	0.4	0.7	0.9	1.2	0.3	0.5	0.7	0.9
	120-139	0.4	0.7	1.0	1.4	0.2	0.3	0.5	0.7	0.4	0.5	0.8	1.0	0.3	0.4	0.5	0.7
	100-119	0.4	0.6	0.9	1.3	0.2	0.3	0.5	0.6	0.3	0.5	0.6	0.8	0.2	0.3	0.5	0.6
80 - 84	160-179	0.7	1.0	1.5	2.0	0.4	0.6	0.9	1.2	0.7	1.0	1.3	1.7	0.5	0.7	1.0	1.4
	140-159	0.6	1.0	1.4	1.8	0.3	0.5	0.8	1.1	0.6	0.9	1.2	1.5	0.4	0.6	0.9	1.2
	120-139	0.6	0.9	1.2	1.7	0.3	0.5	0.7	1.0	0.5	0.7	1.0	1.3	0.4	0.5	0.8	1.0
	100-119	0.5	0.8	1.1	1.5	0.3	0.5	0.6	0.9	0.4	0.6	0.8	1.1	0.3	0.5	0.6	0.8
75 - 79	160-179	0.8	1.3	1.7	2.3	0.5	0.8	1.1	1.5	0.8	1.2	1.6	2.1	0.7	1.0	1.3	1.7
	140-159	0.8	1.2	1.6	2.1	0.5	0.7	1.0	1.4	0.7	1.1	1.4	1.8	0.6	0.8	1.1	1.5
	120-139	0.7	1.1	1.5	1.9	0.4	0.7	0.9	1.2	0.6	0.9	1.2	1.6	0.5	0.7	1.0	1.3
	100-119	0.6	1.0	1.3	1.8	0.4	0.6	0.8	1.1	0.5	0.8	1.0	1.4	0.4	0.6	0.8	1.1
70 - 74	160-179	1.0	1.4	2.0	2.6	0.7	1.0	1.4	1.8	1.0	1.4	1.9	2.5	0.9	1.3	1.7	2.2
	140-159	0.9	1.3	1.8	2.3	0.6	0.9	1.2	1.7	0.9	1.2	1.7	2.2	0.7	1.1	1.5	1.9
	120-139	0.8	1.2	1.6	2.1	0.5	0.8	1.1	1.5	0.7	1.1	1.4	1.9	0.6	0.9	1.3	1.7
	100-119	0.7	1.1	1.5	1.9	0.5	0.7	1.0	1.3	0.6	0.9	1.2	1.6	0.5	0.8	1.1	1.4
65 - 69	160-179	1.1	1.6	2.2	2.8	0.8	1.2	1.7	2.2	1.2	1.7	2.3	2.9	1.1	1.6	2.2	2.8
	140-159	1.0	1.4	1.9	2.5	0.7	1.1	1.5	1.9	1.0	1.4	1.9	2.5	0.9	1.4	1.9	2.4
	120-139	0.9	1.3	1.7	2.3	0.6	0.9	1.3	1.7	0.8	1.2	1.7	2.1	0.8	1.2	1.6	2.1
	100-119	0.8	1.2	1.6	2.1	0.6	0.9	1.3	1.7	0.8	1.1	1.5	2.0	0.8	1.2	1.6	2.1
60 - 64	160-179	1.2	1.7	2.3	3.0	1.0	1.4	2.0	2.6	1.3	1.9	2.6	3.4	1.3	1.9	2.6	3.4
	140-159	1.0	1.5	2.1	2.7	0.8	1.2	1.7	2.2	1.1	1.6	2.2	2.8	1.1	1.6	2.2	3.0
	120-139	0.9	1.4	1.9	2.4	0.7	1.1	1.5	2.0	0.9	1.4	1.8	2.4	0.9	1.4	1.9	2.5
	100-119	0.8	1.2	1.6	2.1	0.6	0.9	1.3	1.7	0.8	1.1	1.5	2.0	0.8	1.2	1.6	2.1
55 - 59	160-179	1.2	1.8	2.5	3.3	1.1	1.7	2.3	3.0	1.5	2.2	3.0	3.8	1.5	2.2	3.1	4.1
	140-159	1.1	1.6	2.2	2.9	1.0	1.4	1.9	2.6	1.2	1.8	2.4	3.2	1.3	1.9	2.7	3.5
	120-139	1.0	1.4	1.9	2.5	0.8	1.2	1.6	2.2	1.0	1.5	2.0	2.6	1.1	1.6	2.2	2.9
	100-119	0.8	1.3	1.7	2.2	0.7	1.0	1.4	1.9	0.8	1.2	1.7	2.2	0.9	1.3	1.8	2.4
50 - 54	160-179	1.3	1.9	2.6	3.4	1.3	1.9	2.7	3.5	1.6	2.4	3.3	4.3	1.7	2.6	3.6	4.9
	140-159	1.1	1.7	2.3	3.0	1.1	1.6	2.2	2.9	1.3	2.0	2.7	3.5	1.4	2.2	3.1	4.1
	120-139	1.0	1.5	2.0	2.6	0.9	1.3	1.8	2.4	1.1	1.6	2.2	2.8	1.2	1.8	2.5	3.4
	100-119	0.9	1.3	1.8	2.3	0.7	1.1	1.5	2.0	0.9	1.3	1.8	2.3	1.0	1.5	2.1	2.7
45 - 49	160-179	1.4	2.0	2.8	3.6	1.5	2.2	3.0	4.0	1.7	2.6	3.6	4.8	1.9	2.9	4.2	5.6
	140-159	1.2	1.7	2.4	3.1	1.2	1.8	2.4	3.2	1.4	2.1	2.9	3.8	1.6	2.4	3.5	4.7
	120-139	1.0	1.5	2.1	2.7	1.0	1.4	2.0	2.6	1.1	1.7	2.3	3.0	1.3	2.0	2.8	3.8
	100-119	0.9	1.3	1.8	2.3	0.8	1.2	1.6	2.1	0.9	1.4	1.9	2.4	1.1	1.6	2.3	3.0
40 - 44	160-179	1.4	2.1	2.9	3.8	1.6	2.4	3.4	4.6	1.8	2.7	3.8	5.1	2.1	3.2	4.6	6.2
	140-159	1.2	1.8	2.4	3.2	1.3	1.9	2.7	3.5	1.5	2.2	3.0	4.0	1.7	2.6	3.8	5.1
	120-139	1.0	1.5	2.1	2.7	1.0	1.5	2.1	2.8	1.2	1.7	2.4	3.2	1.4	2.1	3.0	4.1
	100-119	0.9	1.3	1.8	2.4	0.8	1.2	1.7	2.3	0.9	1.4	1.9	2.5	1.1	1.7	2.4	3.2

Non-HDL cholesterol (mmol/L)

150 200 250
mg/dL

LIFE-CVD2 model

CVD-free lifetime gain (in years) from lifelong 40% LDL cholesterol reduction in the high risk region

Colour key
< 0.5 years
0.5 - 0.9 years
1.0 - 1.4 years
1.5 - 2.0 years
≥ 2.0 years

Age	Systolic BP	Women Non-smoking				Women Smoking				Men Non-smoking				Men Smoking			
		3.0-3.9	4.0-4.9	5.0-5.9	6.0-6.9	3.0-3.9	4.0-4.9	5.0-5.9	6.0-6.9	3.0-3.9	4.0-4.9	5.0-5.9	6.0-6.9	3.0-3.9	4.0-4.9	5.0-5.9	6.0-6.9
85 - 89	160-179	0.6	1.0	1.4	1.8	0.4	0.6	0.9	1.2	0.5	0.7	1.0	1.3	0.4	0.6	0.7	1.0
	140-159	0.6	0.9	1.3	1.7	0.4	0.6	0.8	1.1	0.4	0.6	0.9	1.1	0.3	0.5	0.7	0.8
	120-139	0.6	0.9	1.2	1.6	0.4	0.5	0.8	1.0	0.4	0.5	0.7	1.0	0.3	0.4	0.6	0.7
	100-119	0.5	0.8	1.2	1.6	0.3	0.5	0.7	1.0	0.3	0.5	0.6	0.8	0.2	0.3	0.5	0.6
80 - 84	160-179	0.8	1.2	1.7	2.2	0.5	0.8	1.2	1.6	0.6	0.9	1.3	1.6	0.5	0.7	1.0	1.3
	140-159	0.8	1.2	1.6	2.1	0.5	0.8	1.1	1.5	0.6	0.8	1.1	1.4	0.4	0.6	0.8	1.1
	120-139	0.7	1.1	1.5	2.0	0.5	0.7	1.0	1.4	0.5	0.7	1.0	1.2	0.4	0.5	0.7	1.0
	100-119	0.7	1.0	1.4	1.9	0.4	0.7	1.0	1.3	0.4	0.6	0.8	1.1	0.3	0.4	0.6	0.8
75 - 79	160-179	1.0	1.5	2.0	2.6	0.7	1.1	1.5	1.9	0.8	1.1	1.5	2.0	0.6	0.9	1.3	1.6
	140-159	0.9	1.4	1.9	2.4	0.7	1.0	1.4	1.8	0.7	1.0	1.3	1.7	0.6	0.8	1.1	1.4
	120-139	0.9	1.3	1.7	2.3	0.6	0.9	1.3	1.7	0.6	0.9	1.2	1.5	0.5	0.7	1.0	1.3
	100-119	0.8	1.2	1.6	2.1	0.6	0.8	1.2	1.5	0.5	0.7	1.0	1.3	0.4	0.6	0.8	1.1
70 - 74	160-179	1.1	1.6	2.2	2.9	0.9	1.3	1.8	2.3	0.9	1.4	1.8	2.4	0.8	1.2	1.6	2.1
	140-159	1.0	1.5	2.1	2.7	0.8	1.2	1.6	2.1	0.8	1.2	1.6	2.1	0.7	1.0	1.4	1.9
	120-139	1.0	1.4	1.9	2.5	0.7	1.1	1.5	1.9	0.7	1.0	1.4	1.8	0.6	0.9	1.3	1.6
	100-119	0.9	1.3	1.8	2.3	0.7	1.0	1.3	1.8	0.6	0.9	1.2	1.5	0.5	0.8	1.1	1.4
65 - 69	160-179	1.2	1.8	2.4	3.1	1.0	1.5	2.1	2.7	1.1	1.6	2.2	2.8	1.0	1.5	2.1	2.7
	140-159	1.1	1.6	2.2	2.9	0.9	1.4	1.9	2.4	1.0	1.4	1.9	2.4	0.9	1.3	1.8	2.4
	120-139	1.0	1.5	2.0	2.6	0.8	1.2	1.7	2.2	0.8	1.2	1.6	2.1	0.7	1.1	1.5	2.0
	100-119	1.0	1.4	1.9	2.5	0.8	1.2	1.6	2.1	0.8	1.1	1.5	1.9	0.7	1.1	1.5	2.0
60 - 64	160-179	1.3	1.9	2.6	3.4	1.2	1.8	2.4	3.2	1.3	1.9	2.6	3.3	1.2	1.8	2.5	3.3
	140-159	1.2	1.7	2.3	3.0	1.1	1.6	2.1	2.8	1.1	1.6	2.2	2.8	1.0	1.5	2.2	2.9
	120-139	1.1	1.6	2.1	2.7	0.9	1.4	1.9	2.4	0.9	1.3	1.8	2.3	0.9	1.3	1.8	2.4
	100-119	1.0	1.4	1.9	2.5	0.8	1.2	1.6	2.1	0.8	1.1	1.5	1.9	0.7	1.1	1.5	2.0
55 - 59	160-179	1.4	2.0	2.8	3.6	1.4	2.1	2.8	3.7	1.4	2.1	2.9	3.8	1.4	2.1	2.9	3.9
	140-159	1.2	1.8	2.5	3.2	1.2	1.8	2.4	3.1	1.2	1.7	2.4	3.1	1.2	1.8	2.5	3.4
	120-139	1.1	1.6	2.2	2.8	1.0	1.5	2.1	2.7	1.0	1.4	2.0	2.6	1.0	1.5	2.1	2.9
	100-119	1.0	1.4	2.0	2.5	0.9	1.3	1.8	2.3	0.8	1.2	1.6	2.1	0.8	1.3	1.8	2.4
50 - 54	160-179	1.5	2.1	2.9	3.8	1.6	2.3	3.2	4.3	1.5	2.3	3.2	4.2	1.6	2.4	3.4	4.6
	140-159	1.3	1.9	2.5	3.3	1.3	2.0	2.7	3.5	1.3	1.9	2.6	3.4	1.3	2.0	2.9	3.9
	120-139	1.1	1.7	2.2	2.9	1.1	1.6	2.2	2.9	1.0	1.5	2.1	2.8	1.1	1.7	2.4	3.3
	100-119	1.0	1.5	2.0	2.6	0.9	1.4	1.9	2.5	0.8	1.3	1.7	2.2	0.9	1.4	2.0	2.7
45 - 49	160-179	1.5	2.2	3.0	4.0	1.8	2.7	3.7	4.9	1.6	2.5	3.5	4.7	1.8	2.7	3.9	5.3
	140-159	1.3	1.9	2.6	3.4	1.4	2.1	3.0	3.9	1.3	2.0	2.8	3.7	1.5	2.3	3.3	4.5
	120-139	1.1	1.7	2.3	3.0	1.2	1.7	2.4	3.2	1.1	1.6	2.2	3.0	1.2	1.9	2.7	3.6
	100-119	1.0	1.5	2.0	2.6	1.0	1.4	2.0	2.6	0.9	1.3	1.8	2.4	1.0	1.5	2.1	2.9
40 - 44	160-179	1.6	2.3	3.2	4.1	1.9	3.0	4.2	5.6	1.7	2.6	3.7	5.0	1.9	3.0	4.3	5.8
	140-159	1.3	2.0	2.7	3.5	1.5	2.3	3.2	4.3	1.4	2.1	2.9	3.9	1.6	2.5	3.5	4.9
	120-139	1.2	1.7	2.3	3.0	1.2	1.8	2.5	3.4	1.1	1.7	2.3	3.1	1.3	2.0	2.9	3.9
	100-119	1.0	1.5	2.0	2.7	1.0	1.5	2.0	2.7	0.9	1.3	1.8	2.4	1.0	1.6	2.3	3.1

Non-HDL cholesterol (mmol/L)

150 200 250
mg/dL

LIFE-CVD2 model

CVD-free lifetime gain (in years) from lifelong 40% LDL cholesterol reduction in the very high risk region

Legend
< 0.5 years
0.5 - 0.9 years
1.0 - 1.4 years
1.5 - 2.0 years
≥ 2.0 years

	Women									Men							
	Non-smoking				Smoking				***Age***	Non-smoking				Smoking			
**160-179**	0.7	1.0	1.4	1.8	0.5	0.7	1.0	1.4	**85 - 89**	0.6	0.8	1.1	1.4	0.4	0.6	0.9	1.1
**140-159**	0.6	1.0	1.4	1.8	0.5	0.7	1.0	1.4		0.5	0.8	1.0	1.3	0.4	0.6	0.8	1.0
**120-139**	0.6	1.0	1.3	1.7	0.5	0.7	1.0	1.3		0.5	0.7	0.9	1.2	0.4	0.5	0.7	1.0
**100-119**	0.6	0.9	1.3	1.7	0.5	0.7	1.0	1.3		0.4	0.6	0.9	1.1	0.3	0.5	0.7	0.9
**160-179**	0.9	1.3	1.8	2.3	0.7	1.0	1.4	1.8	**80 - 84**	0.7	1.1	1.4	1.8	0.6	0.8	1.1	1.4
**140-159**	0.9	1.3	1.8	2.3	0.7	1.0	1.4	1.8		0.7	1.0	1.3	1.7	0.5	0.8	1.1	1.4
**120-139**	0.9	1.3	1.7	2.2	0.6	1.0	1.3	1.7		0.6	0.9	1.2	1.6	0.5	0.7	1.0	1.3
**100-119**	0.8	1.2	1.7	2.1	0.6	0.9	1.3	1.7		0.6	0.8	1.1	1.4	0.5	0.7	0.9	1.1
**160-179**	1.1	1.6	2.2	2.8	0.9	1.3	1.8	2.3	**75 - 79**	0.9	1.3	1.8	2.3	0.8	1.1	1.5	2.0
**140-159**	1.1	1.6	2.1	2.7	0.9	1.3	1.7	2.2		0.9	1.3	1.7	2.1	0.7	1.1	1.4	1.8
**120-139**	1.1	1.5	2.1	2.6	0.9	1.2	1.7	2.2		0.8	1.1	1.5	2.0	0.7	1.0	1.3	1.7
**100-119**	1.0	1.5	2.0	2.5	0.8	1.2	1.6	2.1		0.7	1.0	1.4	1.8	0.6	0.9	1.2	1.5
**160-179**	1.3	1.9	2.6	3.3	1.1	1.6	2.2	2.8	**70 - 74**	1.2	1.7	2.3	2.9	1.0	1.5	2.0	2.5
**140-159**	1.3	1.8	2.5	3.1	1.1	1.6	2.1	2.7		1.1	1.6	2.1	2.7	0.9	1.4	1.8	2.4
**120-139**	1.2	1.7	2.3	3.0	1.0	1.5	2.0	2.6		1.0	1.4	1.9	2.4	0.9	1.3	1.7	2.2
**100-119**	1.1	1.7	2.2	2.8	1.0	1.4	1.9	2.5		0.9	1.3	1.7	2.2	0.8	1.1	1.5	2.0
**160-179**	1.5	2.2	2.9	3.7	1.4	2.0	2.6	3.4	**65 - 69**	1.4	2.1	2.8	3.6	1.2	1.8	2.4	3.1
**140-159**	1.4	2.1	2.7	3.5	1.3	1.9	2.5	3.2		1.3	1.9	2.5	3.3	1.2	1.7	2.3	2.9
**120-139**	1.3	1.9	2.6	3.3	1.2	1.8	2.3	3.0		1.2	1.7	2.3	2.9	1.1	1.5	2.1	2.7
**100-119**	1.3	1.9	2.5	3.2	1.3	1.8	2.5	3.1		1.2	1.7	2.3	2.9	1.1	1.6	2.2	2.9
**160-179**	1.7	2.4	3.3	4.2	1.6	2.4	3.2	4.0	**60 - 64**	1.7	2.4	3.3	4.2	1.5	2.1	2.9	3.7
**140-159**	1.6	2.2	3.0	3.8	1.5	2.2	2.9	3.8		1.5	2.2	2.9	3.8	1.4	2.0	2.7	3.5
**120-139**	1.4	2.1	2.8	3.5	1.4	2.0	2.7	3.5		1.3	1.9	2.6	3.4	1.2	1.8	2.5	3.2
**100-119**	1.3	1.9	2.5	3.2	1.3	1.8	2.5	3.1		1.2	1.7	2.3	2.9	1.1	1.6	2.2	2.9
**160-179**	1.9	2.7	3.6	4.6	1.9	2.8	3.8	4.9	**55 - 59**	1.9	2.8	3.8	4.9	1.7	2.5	3.4	4.4
**140-159**	1.7	2.4	3.2	4.1	1.7	2.5	3.4	4.4		1.7	2.4	3.3	4.3	1.6	2.3	3.2	4.2
**120-139**	1.5	2.2	2.9	3.7	1.6	2.3	3.1	3.9		1.5	2.1	2.9	3.8	1.4	2.1	2.9	3.8
**100-119**	1.4	2.0	2.7	3.4	1.4	2.0	2.7	3.5		1.3	1.8	2.5	3.2	1.3	1.9	2.6	3.4
**160-179**	2.0	2.9	3.9	5.0	2.2	3.3	4.4	5.7	**50 - 54**	2.1	3.1	4.2	5.6	1.9	2.9	3.9	5.1
**140-159**	1.8	2.6	3.4	4.4	2.0	2.9	3.9	5.1		1.8	2.7	3.7	4.9	1.8	2.7	3.7	4.8
**120-139**	1.6	2.3	3.1	3.9	1.7	2.5	3.4	4.4		1.6	2.3	3.2	4.2	1.6	2.4	3.3	4.4
**100-119**	1.4	2.0	2.7	3.5	1.5	2.2	3.0	3.8		1.3	2.0	2.7	3.5	1.4	2.1	2.9	3.9
**160-179**	2.1	3.1	4.2	5.5	2.5	3.7	5.1	6.5	**45 - 49**	2.3	3.4	4.7	6.2	2.2	3.3	4.5	5.9
**140-159**	1.9	2.7	3.6	4.7	2.2	3.3	4.5	5.8		2.0	2.9	4.1	5.4	2.0	3.0	4.2	5.5
**120-139**	1.6	2.4	3.2	4.1	1.9	2.8	3.8	4.9		1.7	2.5	3.4	4.5	1.8	2.7	3.7	5.0
**100-119**	1.4	2.1	2.8	3.6	1.6	2.3	3.2	4.1		1.4	2.1	2.9	3.8	1.5	2.3	3.2	4.3
**160-179**	2.3	3.3	4.5	5.9	2.8	4.1	5.6	7.3	**40 - 44**	2.4	3.6	5.1	6.7	2.4	3.6	5.0	6.5
**140-159**	1.9	2.8	3.8	4.9	2.4	3.6	4.9	6.5		2.0	3.1	4.3	5.7	2.2	3.3	4.6	6.1
**120-139**	1.7	2.4	3.3	4.2	2.0	3.0	4.1	5.4		1.7	2.6	3.6	4.8	1.9	2.9	4.0	5.4
**100-119**	1.5	2.1	2.9	3.7	1.7	2.5	3.4	4.4		1.4	2.1	3.0	3.9	1.6	2.4	3.5	4.7
	**3.0-3.9**	**4.0-4.9**	**5.0-5.9**	**6.0-6.9**	**3.0-3.9**	**4.0-4.9**	**5.0-5.9**	**6.0-6.9**		**3.0-3.9**	**4.0-4.9**	**5.0-5.9**	**6.0-6.9**	**3.0-3.9**	**4.0-4.9**	**5.0-5.9**	**6.0-6.9**

Non-HDL cholesterol (mmol/L)

150 200 250
mg/dL

LIFE-CVD2 model

CVD-free lifetime gain from smoking cessation (in years) in the low risk region

Legend
< 0.5 years
0.5 - 0.9 years
1.0 - 1.4 years
1.5 - 2.0 years
≥ 2.0 years

Age		Women Non-smoking 3.0-3.9	Women Non-smoking 4.0-4.9	Women Non-smoking 5.0-5.9	Women Non-smoking 6.0-6.9	Women Smoking 3.0-3.9	Women Smoking 4.0-4.9	Women Smoking 5.0-5.9	Women Smoking 6.0-6.9	Men Non-smoking 3.0-3.9	Men Non-smoking 4.0-4.9	Men Non-smoking 5.0-5.9	Men Non-smoking 6.0-6.9	Men Smoking 3.0-3.9	Men Smoking 4.0-4.9	Men Smoking 5.0-5.9	Men Smoking 6.0-6.9
85 - 89	160-179	-	-	-	-	1.2	1.4	1.6	1.8	-	-	-	-	1.4	1.5	1.6	1.7
	140-159	-	-	-	-	1.2	1.3	1.5	1.7	-	-	-	-	1.3	1.4	1.5	1.6
	120-139	-	-	-	-	1.1	1.3	1.4	1.6	-	-	-	-	1.2	1.2	1.3	1.4
	100-119	-	-	-	-	1.1	1.2	1.4	1.5	-	-	-	-	1.1	1.1	1.2	1.3
80 - 84	160-179	-	-	-	-	1.7	1.9	2.1	2.2	-	-	-	-	1.9	2.0	2.1	2.2
	140-159	-	-	-	-	1.7	1.8	2.0	2.2	-	-	-	-	1.7	1.8	1.9	2.0
	120-139	-	-	-	-	1.6	1.8	1.9	2.1	-	-	-	-	1.6	1.7	1.8	1.9
	100-119	-	-	-	-	1.6	1.7	1.8	2.0	-	-	-	-	1.5	1.6	1.6	1.7
75 - 79	160-179	-	-	-	-	2.2	2.4	2.6	2.8	-	-	-	-	2.3	2.4	2.6	2.7
	140-159	-	-	-	-	2.1	2.3	2.5	2.6	-	-	-	-	2.2	2.3	2.4	2.5
	120-139	-	-	-	-	2.1	2.2	2.4	2.5	-	-	-	-	2.0	2.1	2.2	2.3
	100-119	-	-	-	-	2.0	2.1	2.3	2.4	-	-	-	-	1.9	2.0	2.1	2.2
70 - 74	160-179	-	-	-	-	2.7	2.8	3.0	3.2	-	-	-	-	2.9	3.0	3.1	3.3
	140-159	-	-	-	-	2.5	2.7	2.9	3.1	-	-	-	-	2.7	2.8	2.9	3.0
	120-139	-	-	-	-	2.4	2.6	2.8	2.9	-	-	-	-	2.5	2.6	2.7	2.8
	100-119	-	-	-	-	2.4	2.5	2.6	2.8	-	-	-	-	2.4	2.5	2.5	2.6
65 - 69	160-179	-	-	-	-	3.1	3.2	3.4	3.7	-	-	-	-	3.5	3.6	3.8	3.9
	140-159	-	-	-	-	2.9	3.1	3.3	3.5	-	-	-	-	3.2	3.3	3.5	3.6
	120-139	-	-	-	-	2.8	2.9	3.1	3.3	-	-	-	-	3.0	3.1	3.2	3.3
	100-119	-	-	-	-	2.9	3.0	3.2	3.4	-	-	-	-	3.1	3.2	3.3	3.4
60 - 64	160-179	-	-	-	-	3.5	3.7	3.9	4.1	-	-	-	-	4.0	4.2	4.4	4.6
	140-159	-	-	-	-	3.2	3.4	3.6	3.8	-	-	-	-	3.7	3.8	4.0	4.2
	120-139	-	-	-	-	3.1	3.2	3.4	3.6	-	-	-	-	3.4	3.5	3.6	3.8
	100-119	-	-	-	-	2.9	3.0	3.2	3.4	-	-	-	-	3.1	3.2	3.3	3.4
55 - 59	160-179	-	-	-	-	3.8	4.1	4.3	4.6	-	-	-	-	4.6	4.8	5.0	5.3
	140-159	-	-	-	-	3.6	3.7	4.0	4.2	-	-	-	-	4.2	4.3	4.5	4.8
	120-139	-	-	-	-	3.3	3.5	3.7	3.9	-	-	-	-	3.8	3.9	4.1	4.2
	100-119	-	-	-	-	3.1	3.2	3.4	3.6	-	-	-	-	3.4	3.5	3.6	3.8
50 - 54	160-179	-	-	-	-	4.2	4.5	4.7	5.0	-	-	-	-	5.1	5.4	5.7	6.0
	140-159	-	-	-	-	3.8	4.0	4.3	4.5	-	-	-	-	4.6	4.8	5.0	5.3
	120-139	-	-	-	-	3.5	3.7	3.9	4.1	-	-	-	-	4.1	4.2	4.4	4.7
	100-119	-	-	-	-	3.2	3.4	3.6	3.8	-	-	-	-	3.7	3.8	3.9	4.1
45 - 49	160-179	-	-	-	-	4.5	4.8	5.2	5.5	-	-	-	-	5.6	5.9	6.3	6.7
	140-159	-	-	-	-	4.1	4.3	4.6	4.9	-	-	-	-	4.9	5.2	5.5	5.8
	120-139	-	-	-	-	3.7	3.9	4.1	4.3	-	-	-	-	4.3	4.5	4.7	5.0
	100-119	-	-	-	-	3.4	3.5	3.7	3.9	-	-	-	-	3.8	4.0	4.1	4.3
40 - 44	160-179	-	-	-	-	4.8	5.2	5.6	6.0	-	-	-	-	5.9	6.3	6.8	7.3
	140-159	-	-	-	-	4.3	4.5	4.8	5.2	-	-	-	-	5.1	5.4	5.8	6.2
	120-139	-	-	-	-	3.8	4.0	4.3	4.5	-	-	-	-	4.5	4.7	5.0	5.3
	100-119	-	-	-	-	3.5	3.6	3.8	4.1	-	-	-	-	4.0	4.1	4.3	4.5

Non-HDL cholesterol (mmol/L)

150 200 250
mg/dL

LIFE-CVD2 model

CVD-free lifetime gain from smoking cessation (in years) in the moderate risk region

Legend
< 0.5 years
0.5 - 0.9 years
1.0 - 1.4 years
1.5 - 2.0 years
≥ 2.0 years

		Women								Men							
		Non-smoking				Smoking				Non-smoking				Smoking			
Age		3.0-3.9	4.0-4.9	5.0-5.9	6.0-6.9	3.0-3.9	4.0-4.9	5.0-5.9	6.0-6.9	3.0-3.9	4.0-4.9	5.0-5.9	6.0-6.9	3.0-3.9	4.0-4.9	5.0-5.9	6.0-6.9
85 - 89	160-179	-	-	-	-	1.3	1.4	1.6	1.8	-	-	-	-	1.4	1.5	1.6	1.7
	140-159	-	-	-	-	1.2	1.4	1.5	1.7	-	-	-	-	1.3	1.4	1.5	1.6
	120-139	-	-	-	-	1.2	1.3	1.4	1.6	-	-	-	-	1.2	1.3	1.4	1.5
	100-119	-	-	-	-	1.1	1.2	1.4	1.5	-	-	-	-	1.1	1.2	1.3	1.3
80 - 84	160-179	-	-	-	-	1.8	1.9	2.1	2.3	-	-	-	-	1.9	2.0	2.1	2.2
	140-159	-	-	-	-	1.7	1.9	2.0	2.2	-	-	-	-	1.8	1.9	2.0	2.1
	120-139	-	-	-	-	1.7	1.8	2.0	2.1	-	-	-	-	1.7	1.7	1.8	1.9
	100-119	-	-	-	-	1.6	1.7	1.9	2.0	-	-	-	-	1.6	1.6	1.7	1.8
75 - 79	160-179	-	-	-	-	2.3	2.4	2.6	2.8	-	-	-	-	2.4	2.5	2.6	2.7
	140-159	-	-	-	-	2.2	2.4	2.5	2.7	-	-	-	-	2.3	2.4	2.5	2.6
	120-139	-	-	-	-	2.1	2.3	2.4	2.6	-	-	-	-	2.1	2.2	2.3	2.4
	100-119	-	-	-	-	2.0	2.2	2.3	2.5	-	-	-	-	2.0	2.1	2.2	2.3
70 - 74	160-179	-	-	-	-	2.7	2.9	3.1	3.3	-	-	-	-	3.0	3.1	3.2	3.4
	140-159	-	-	-	-	2.6	2.8	3.0	3.2	-	-	-	-	2.8	2.9	3.1	3.2
	120-139	-	-	-	-	2.5	2.7	2.8	3.0	-	-	-	-	2.7	2.8	2.9	3.0
	100-119	-	-	-	-	2.4	2.6	2.7	2.9	-	-	-	-	2.5	2.6	2.7	2.8
65 - 69	160-179	-	-	-	-	3.2	3.4	3.6	3.8	-	-	-	-	3.6	3.8	3.9	4.1
	140-159	-	-	-	-	3.0	3.2	3.4	3.6	-	-	-	-	3.4	3.5	3.7	3.8
	120-139	-	-	-	-	2.9	3.0	3.2	3.4	-	-	-	-	3.2	3.3	3.4	3.6
	100-119	-	-	-	-	3.0	3.1	3.3	3.5	-	-	-	-	3.3	3.5	3.6	3.7
60 - 64	160-179	-	-	-	-	3.6	3.8	4.1	4.3	-	-	-	-	4.2	4.4	4.6	4.9
	140-159	-	-	-	-	3.4	3.6	3.8	4.0	-	-	-	-	3.9	4.1	4.3	4.5
	120-139	-	-	-	-	3.2	3.3	3.5	3.7	-	-	-	-	3.6	3.8	3.9	4.1
	100-119	-	-	-	-	3.0	3.1	3.3	3.5	-	-	-	-	3.3	3.5	3.6	3.7
55 - 59	160-179	-	-	-	-	4.1	4.3	4.6	4.9	-	-	-	-	4.8	5.1	5.4	5.6
	140-159	-	-	-	-	3.7	3.9	4.2	4.4	-	-	-	-	4.4	4.6	4.9	5.1
	120-139	-	-	-	-	3.5	3.6	3.8	4.1	-	-	-	-	4.0	4.2	4.4	4.6
	100-119	-	-	-	-	3.2	3.4	3.6	3.7	-	-	-	-	3.7	3.8	4.0	4.2
50 - 54	160-179	-	-	-	-	4.5	4.8	5.1	5.4	-	-	-	-	5.4	5.7	6.1	6.5
	140-159	-	-	-	-	4.0	4.3	4.5	4.8	-	-	-	-	4.9	5.2	5.5	5.8
	120-139	-	-	-	-	3.7	3.9	4.1	4.4	-	-	-	-	4.4	4.6	4.9	5.1
	100-119	-	-	-	-	3.4	3.6	3.7	4.0	-	-	-	-	4.0	4.1	4.3	4.5
45 - 49	160-179	-	-	-	-	4.9	5.2	5.6	6.0	-	-	-	-	5.9	6.3	6.8	7.2
	140-159	-	-	-	-	4.3	4.6	4.9	5.2	-	-	-	-	5.3	5.6	6.0	6.4
	120-139	-	-	-	-	3.9	4.1	4.3	4.6	-	-	-	-	4.7	4.9	5.2	5.6
	100-119	-	-	-	-	3.5	3.7	3.9	4.1	-	-	-	-	4.2	4.4	4.6	4.9
40 - 44	160-179	-	-	-	-	5.2	5.6	6.1	6.6	-	-	-	-	6.3	6.8	7.3	7.9
	140-159	-	-	-	-	4.5	4.8	5.2	5.6	-	-	-	-	5.6	6.0	6.4	6.9
	120-139	-	-	-	-	4.0	4.3	4.5	4.8	-	-	-	-	4.9	5.2	5.5	6.0
	100-119	-	-	-	-	3.6	3.8	4.0	4.3	-	-	-	-	4.3	4.5	4.8	5.1

Non-HDL cholesterol (mmol/L)

150 200 250
mg/dL

LIFE-CVD2 model

CVD-free lifetime gain from smoking cessation reduction (in years) in the high risk region

Colour	Years
Lightest	< 0.5 years
Light	0.5 - 0.9 years
Medium	1.0 - 1.4 years
Darker	1.5 - 2.0 years
Darkest	≥ 2.0 years

Non-HDL cholesterol (mmol/L)

Age	Systolic BP	Women Non-smoking 3.0-3.9	Women Non-smoking 4.0-4.9	Women Non-smoking 5.0-5.9	Women Non-smoking 6.0-6.9	Women Smoking 3.0-3.9	Women Smoking 4.0-4.9	Women Smoking 5.0-5.9	Women Smoking 6.0-6.9	Men Non-smoking 3.0-3.9	Men Non-smoking 4.0-4.9	Men Non-smoking 5.0-5.9	Men Non-smoking 6.0-6.9	Men Smoking 3.0-3.9	Men Smoking 4.0-4.9	Men Smoking 5.0-5.9	Men Smoking 6.0-6.9
85 - 89	160-179	-	-	-	-	1.3	1.4	1.6	1.7	-	-	-	-	1.3	1.3	1.4	1.5
	140-159	-	-	-	-	1.3	1.4	1.5	1.7	-	-	-	-	1.2	1.3	1.3	1.4
	120-139	-	-	-	-	1.3	1.4	1.5	1.6	-	-	-	-	1.1	1.2	1.2	1.3
	100-119	-	-	-	-	1.2	1.3	1.5	1.6	-	-	-	-	1.0	1.1	1.1	1.2
80 - 84	160-179	-	-	-	-	1.8	2.0	2.1	2.3	-	-	-	-	1.7	1.8	1.8	1.9
	140-159	-	-	-	-	1.8	1.9	2.1	2.2	-	-	-	-	1.6	1.7	1.8	1.8
	120-139	-	-	-	-	1.8	1.9	2.0	2.2	-	-	-	-	1.5	1.6	1.7	1.7
	100-119	-	-	-	-	1.7	1.8	2.0	2.1	-	-	-	-	1.4	1.5	1.6	1.7
75 - 79	160-179	-	-	-	-	2.3	2.5	2.6	2.8	-	-	-	-	2.2	2.3	2.4	2.5
	140-159	-	-	-	-	2.3	2.4	2.6	2.7	-	-	-	-	2.1	2.2	2.3	2.4
	120-139	-	-	-	-	2.2	2.4	2.5	2.7	-	-	-	-	2.0	2.1	2.2	2.3
	100-119	-	-	-	-	2.2	2.3	2.4	2.6	-	-	-	-	1.9	2.0	2.1	2.1
70 - 74	160-179	-	-	-	-	2.8	3.0	3.2	3.3	-	-	-	-	2.8	2.9	3.1	3.2
	140-159	-	-	-	-	2.8	2.9	3.1	3.2	-	-	-	-	2.7	2.8	2.9	3.0
	120-139	-	-	-	-	2.7	2.8	2.9	3.1	-	-	-	-	2.6	2.7	2.8	2.9
	100-119	-	-	-	-	2.6	2.7	2.8	3.0	-	-	-	-	2.4	2.5	2.6	2.7
65 - 69	160-179	-	-	-	-	3.3	3.5	3.7	3.9	-	-	-	-	3.5	3.6	3.7	3.9
	140-159	-	-	-	-	3.2	3.3	3.5	3.7	-	-	-	-	3.3	3.4	3.5	3.7
	120-139	-	-	-	-	3.0	3.2	3.3	3.5	-	-	-	-	3.1	3.2	3.3	3.5
	100-119	-	-	-	-	3.2	3.3	3.5	3.7	-	-	-	-	3.3	3.4	3.6	3.7
60 - 64	160-179	-	-	-	-	3.8	4.0	4.2	4.5	-	-	-	-	4.1	4.2	4.4	4.6
	140-159	-	-	-	-	3.6	3.8	4.0	4.2	-	-	-	-	3.8	4.0	4.1	4.3
	120-139	-	-	-	-	3.4	3.5	3.7	3.9	-	-	-	-	3.6	3.7	3.9	4.0
	100-119	-	-	-	-	3.2	3.3	3.5	3.7	-	-	-	-	3.3	3.4	3.6	3.7
55 - 59	160-179	-	-	-	-	4.4	4.6	4.9	5.1	-	-	-	-	4.7	4.9	5.1	5.4
	140-159	-	-	-	-	4.0	4.2	4.4	4.7	-	-	-	-	4.3	4.5	4.8	5.0
	120-139	-	-	-	-	3.7	3.9	4.1	4.3	-	-	-	-	4.0	4.2	4.4	4.6
	100-119	-	-	-	-	3.4	3.6	3.8	3.9	-	-	-	-	3.7	3.8	4.0	4.2
50 - 54	160-179	-	-	-	-	4.8	5.2	5.5	5.9	-	-	-	-	5.2	5.5	5.8	6.2
	140-159	-	-	-	-	4.4	4.6	4.9	5.2	-	-	-	-	4.8	5.1	5.3	5.7
	120-139	-	-	-	-	4.0	4.2	4.4	4.6	-	-	-	-	4.4	4.6	4.8	5.1
	100-119	-	-	-	-	3.6	3.8	4.0	4.2	-	-	-	-	4.0	4.1	4.3	4.5
45 - 49	160-179	-	-	-	-	5.3	5.7	6.1	6.6	-	-	-	-	5.7	6.1	6.5	6.9
	140-159	-	-	-	-	4.7	5.0	5.3	5.7	-	-	-	-	5.2	5.5	5.9	6.3
	120-139	-	-	-	-	4.2	4.4	4.7	5.0	-	-	-	-	4.7	4.9	5.2	5.6
	100-119	-	-	-	-	3.8	4.0	4.2	4.4	-	-	-	-	4.2	4.4	4.6	4.9
40 - 44	160-179	-	-	-	-	5.7	6.2	6.8	7.4	-	-	-	-	6.1	6.5	7.0	7.5
	140-159	-	-	-	-	4.9	5.3	5.7	6.1	-	-	-	-	5.5	5.8	6.3	6.7
	120-139	-	-	-	-	4.3	4.6	4.9	5.2	-	-	-	-	4.9	5.1	5.5	5.9
	100-119	-	-	-	-	3.9	4.1	4.3	4.6	-	-	-	-	4.3	4.5	4.8	5.1

150 200 250 mg/dL

LIFE-CVD2 model

CVD-free lifetime gain from smoking cessation (in years) in the very high risk region

Shade	Gain
	< 0.5 years
	0.5 - 0.9 years
	1.0 - 1.4 years
	1.5 - 2.0 years
	≥ 2.0 years

	Women									Men							
	Non-smoking				Smoking				*Age*	Non-smoking				Smoking			
160-179	-	-	-	-	1.3	1.4	1.5	1.6	85 - 89	-	-	-	-	1.2	1.3	1.3	1.4
140-159	-	-	-	-	1.3	1.4	1.5	1.6		-	-	-	-	1.2	1.3	1.3	1.4
120-139	-	-	-	-	1.3	1.4	1.5	1.6		-	-	-	-	1.2	1.2	1.3	1.3
100-119	-	-	-	-	1.3	1.4	1.5	1.5		-	-	-	-	1.1	1.2	1.2	1.3
160-179	-	-	-	-	1.8	1.9	2.0	2.1	80 - 84	-	-	-	-	1.7	1.7	1.8	1.8
140-159	-	-	-	-	1.8	1.9	2.0	2.1		-	-	-	-	1.7	1.7	1.7	1.8
120-139	-	-	-	-	1.8	1.9	2.0	2.1		-	-	-	-	1.6	1.7	1.7	1.8
100-119	-	-	-	-	1.8	1.9	2.0	2.1		-	-	-	-	1.6	1.6	1.7	1.7
160-179	-	-	-	-	2.4	2.5	2.6	2.7	75 - 79	-	-	-	-	2.3	2.3	2.4	2.5
140-159	-	-	-	-	2.4	2.5	2.6	2.7		-	-	-	-	2.2	2.3	2.4	2.4
120-139	-	-	-	-	2.3	2.5	2.6	2.7		-	-	-	-	2.2	2.2	2.3	2.4
100-119	-	-	-	-	2.3	2.4	2.5	2.6		-	-	-	-	2.1	2.2	2.2	2.3
160-179	-	-	-	-	2.9	3.1	3.2	3.3	70 - 74	-	-	-	-	2.9	3.0	3.1	3.1
140-159	-	-	-	-	2.9	3.0	3.1	3.3		-	-	-	-	2.9	2.9	3.0	3.1
120-139	-	-	-	-	2.9	3.0	3.1	3.2		-	-	-	-	2.8	2.9	3.0	3.0
100-119	-	-	-	-	2.8	2.9	3.0	3.2		-	-	-	-	2.7	2.8	2.9	3.0
160-179	-	-	-	-	3.6	3.7	3.8	4.0	65 - 69	-	-	-	-	3.6	3.7	3.7	3.8
140-159	-	-	-	-	3.5	3.6	3.7	3.9		-	-	-	-	3.5	3.6	3.7	3.8
120-139	-	-	-	-	3.4	3.5	3.6	3.8		-	-	-	-	3.4	3.5	3.6	3.7
100-119	-	-	-	-	3.7	3.8	4.0	4.1		-	-	-	-	3.8	3.9	4.0	4.2
160-179	-	-	-	-	4.2	4.4	4.6	4.8	60 - 64	-	-	-	-	4.2	4.3	4.4	4.5
140-159	-	-	-	-	4.1	4.2	4.4	4.6		-	-	-	-	4.1	4.2	4.4	4.5
120-139	-	-	-	-	3.9	4.0	4.2	4.4		-	-	-	-	4.0	4.1	4.2	4.4
100-119	-	-	-	-	3.7	3.8	4.0	4.1		-	-	-	-	3.8	3.9	4.0	4.2
160-179	-	-	-	-	5.0	5.2	5.4	5.7	55 - 59	-	-	-	-	4.9	5.0	5.1	5.2
140-159	-	-	-	-	4.7	4.9	5.1	5.4		-	-	-	-	4.7	4.9	5.1	5.2
120-139	-	-	-	-	4.4	4.6	4.8	5.0		-	-	-	-	4.5	4.7	4.9	5.1
100-119	-	-	-	-	4.1	4.2	4.4	4.6		-	-	-	-	4.2	4.4	4.6	4.8
160-179	-	-	-	-	5.7	6.0	6.3	6.6	50 - 54	-	-	-	-	5.5	5.7	5.9	6.0
140-159	-	-	-	-	5.3	5.5	5.8	6.2		-	-	-	-	5.3	5.5	5.8	6.0
120-139	-	-	-	-	4.8	5.0	5.3	5.6		-	-	-	-	5.0	5.2	5.5	5.8
100-119	-	-	-	-	4.4	4.6	4.8	5.1		-	-	-	-	4.7	4.9	5.1	5.4
160-179	-	-	-	-	6.5	6.8	7.2	7.5	45 - 49	-	-	-	-	6.1	6.4	6.6	6.8
140-159	-	-	-	-	5.8	6.2	6.6	7.0		-	-	-	-	5.9	6.1	6.4	6.7
120-139	-	-	-	-	5.2	5.5	5.9	6.2		-	-	-	-	5.4	5.7	6.1	6.4
100-119	-	-	-	-	4.7	4.9	5.2	5.5		-	-	-	-	5.0	5.3	5.6	5.9
160-179	-	-	-	-	7.1	7.5	8.0	8.4	40 - 44	-	-	-	-	6.6	6.9	7.2	7.4
140-159	-	-	-	-	6.3	6.8	7.3	7.8		-	-	-	-	6.3	6.6	6.9	7.3
120-139	-	-	-	-	5.6	5.9	6.3	6.8		-	-	-	-	5.8	6.1	6.5	6.9
100-119	-	-	-	-	4.9	5.2	5.5	5.9		-	-	-	-	5.2	5.5	5.9	6.3
	3.0-3.9	4.0-4.9	5.0-5.9	6.0-6.9	3.0-3.9	4.0-4.9	5.0-5.9	6.0-6.9		3.0-3.9	4.0-4.9	5.0-5.9	6.0-6.9	3.0-3.9	4.0-4.9	5.0-5.9	6.0-6.9

Non-HDL cholesterol (mmol/L)

150 200 250
mg/dL
