# Supplementary material for: Prediction of individual lifetime cardiovascular risk and potential treatment benefit: development and recalibration of the LIFE-CVD2 model to four European risk regions
Source: Eur J Prev Cardiol. 2024 May 16;31(14):1690–9. doi: 10.1093/eurjpc/zwae174 (PMC11464100; doi:10.1093/eurjpc/zwae174)

# Prediction of individual lifetime cardiovascular risk and potential treatment benefit: development and recalibration of the LIFE-CVD2 model to four European risk regions

# Online Supplement

## Supplementary Figure 1. Risk regions based on standardised CVD mortality rates (From SCORE2, Hageman et al 2021)

## Supplementary Figure 2: Cardiovascular mortality and derived incidence in all risk regions


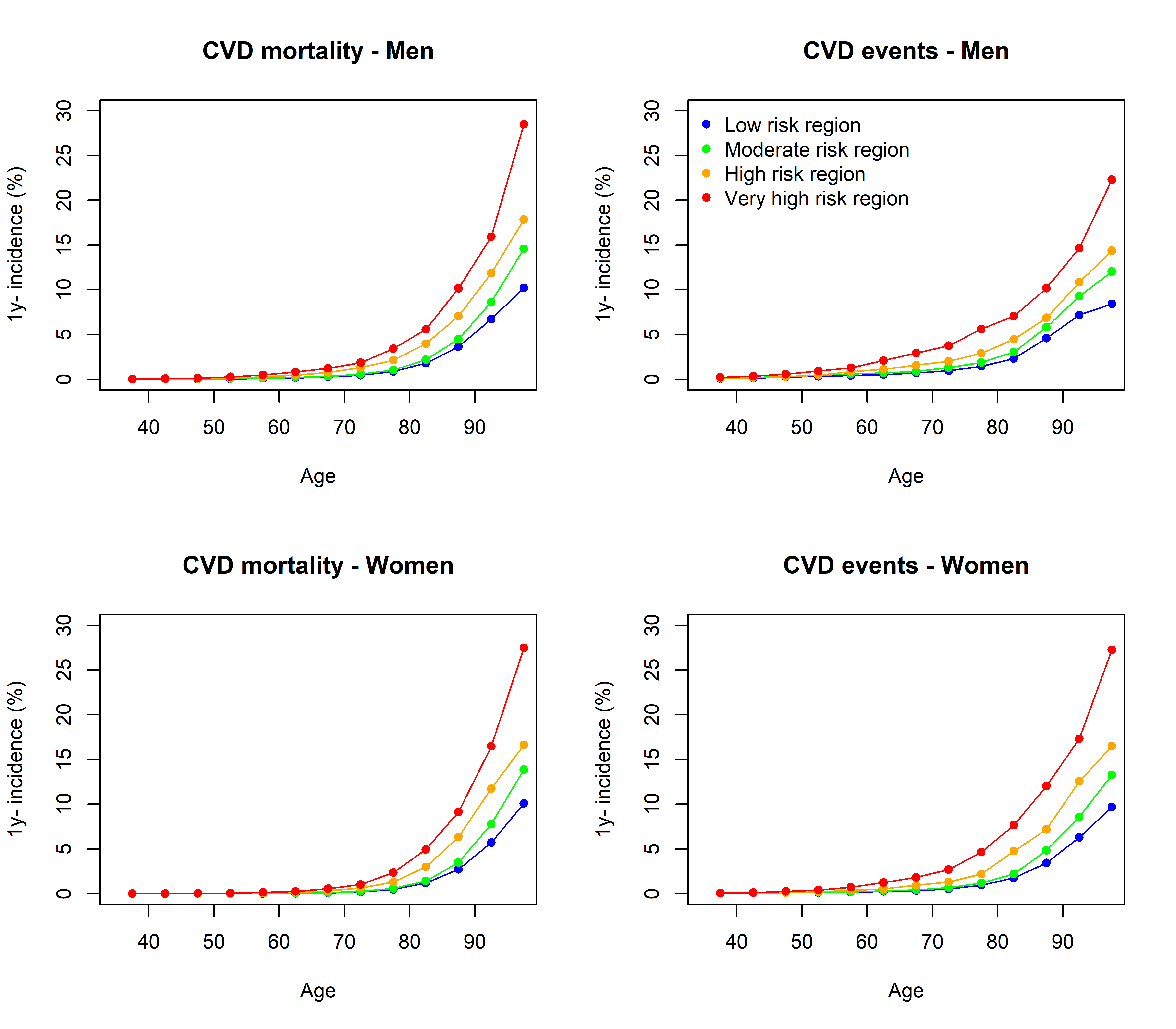


1-year cumulative incidences of cardiovascular mortality in the complete population (left) and fatal or non-fatal cardiovascular events in those without prior cardiovascular disease (right) in every region for every age-group.

## Supplementary Figure 3: Estimated CVD incidence rates and predicted risks


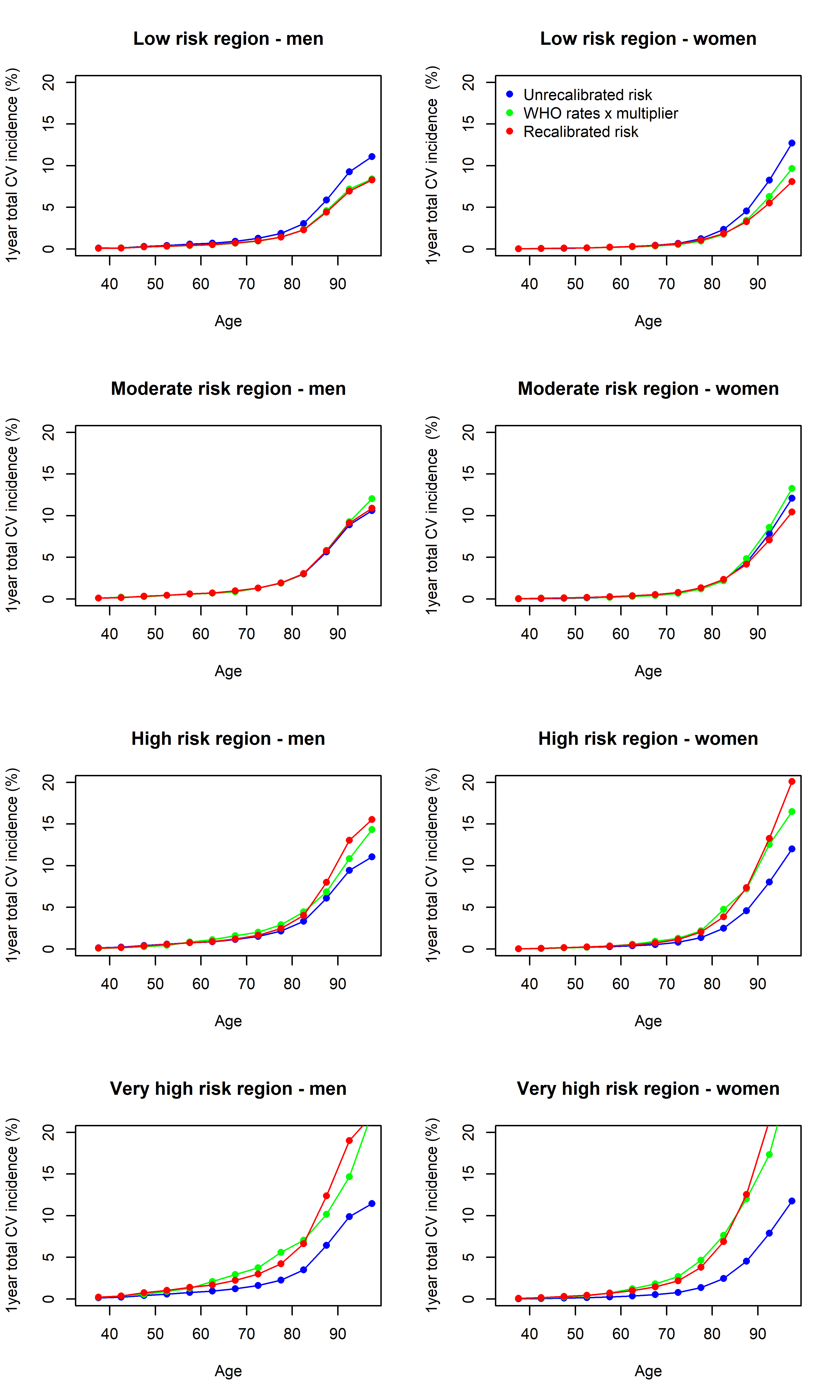


## Supplementary Figure 4: Estimated non-CVD fatal incidence rates and predicted risks


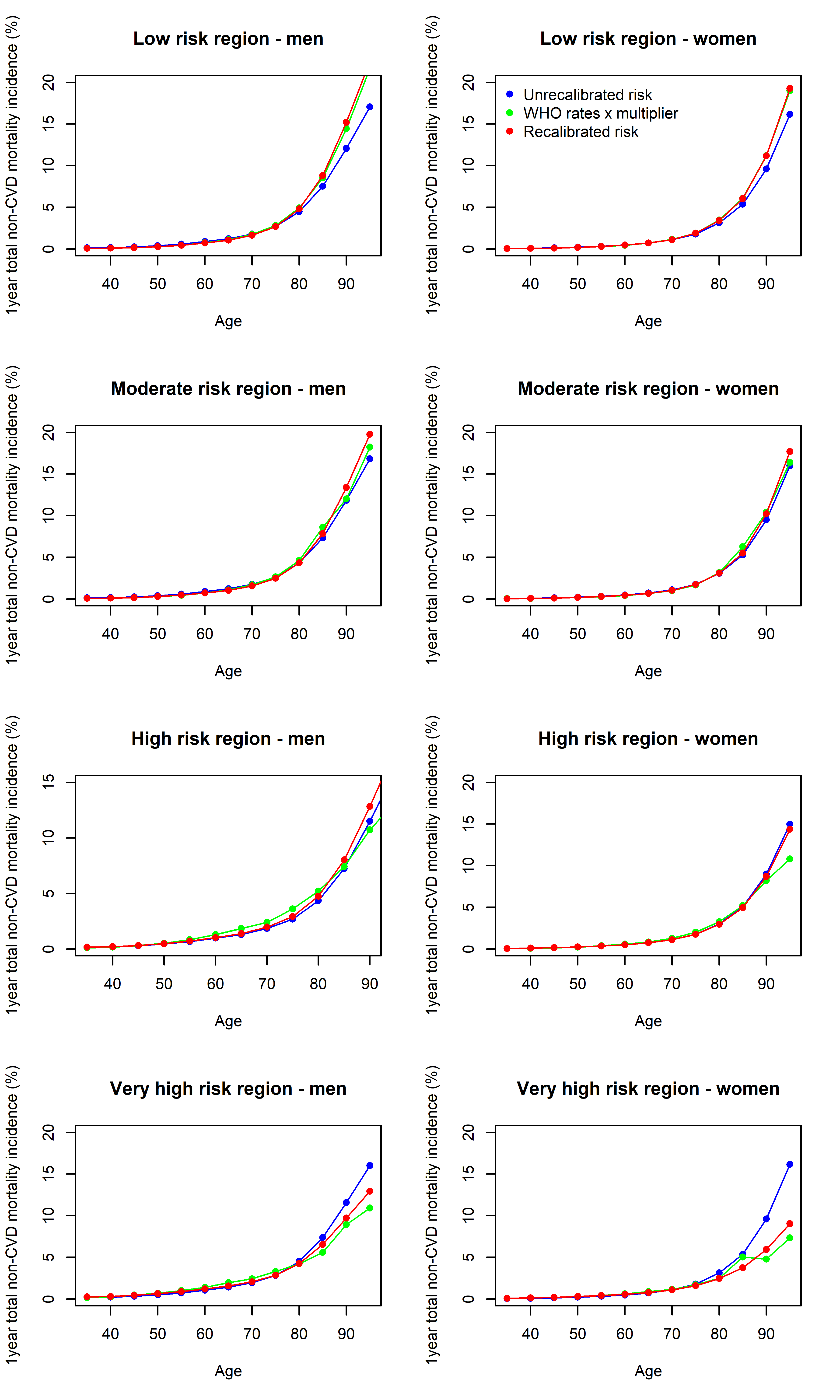


## Supplementary Figure 5: Validation of estimated CVD incidence against independent registry data


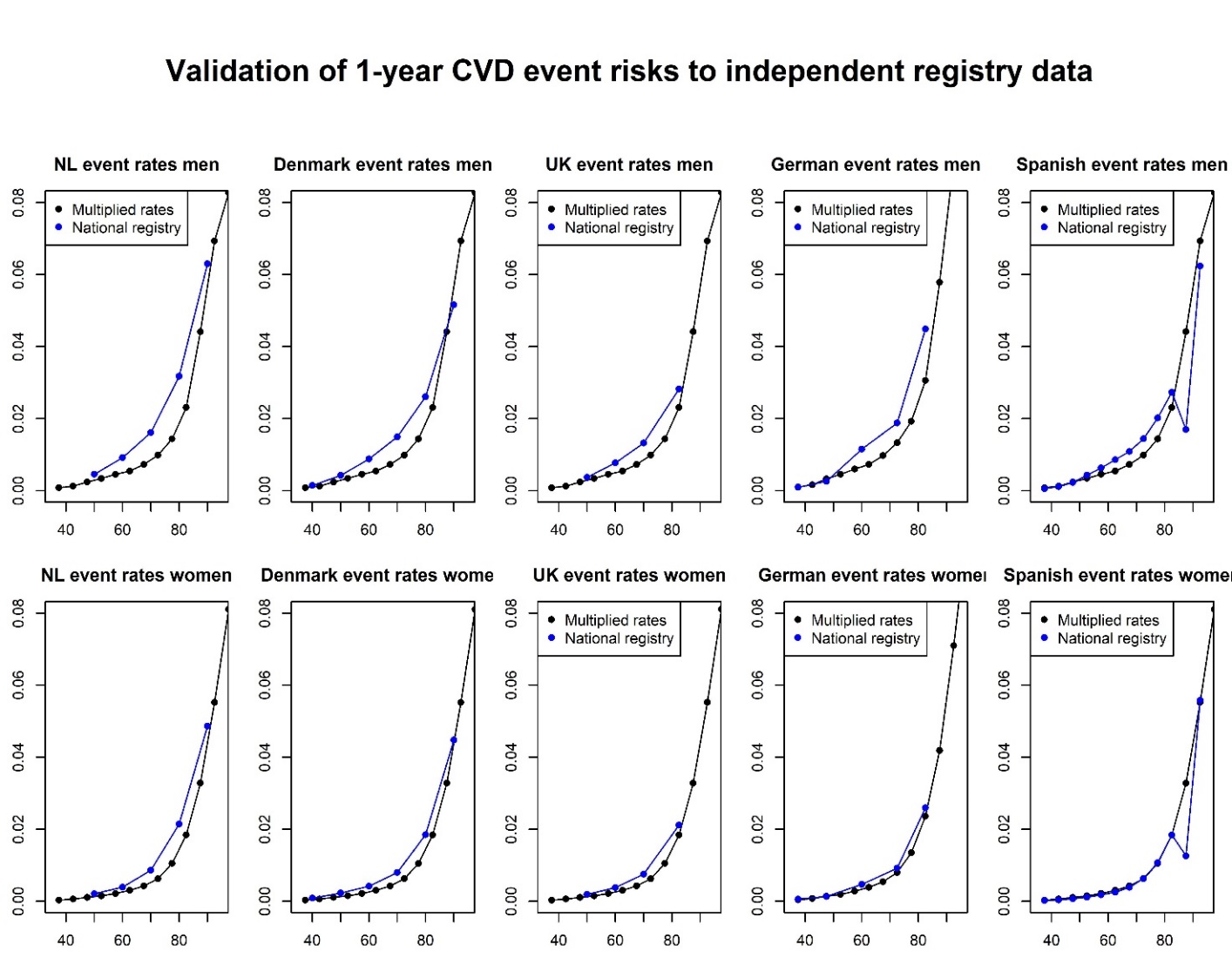


**Registry data sources (all from year 2018):**

|  | Country | Source | Reference | ICD codes included in endpoint |
| --- | --- | --- | --- | --- |
| Mortality data | The Netherlands | CBS statistics Netherlands | https://www.volksgezondheidenzorg.info/ | I0-99 |
|  | Denmark | Hjerteforeningen | https://hjerteforeningen.dk/ | I0-99 |
|  | United Kingdom | Office of National Statistics | https://www.nomisweb.co.uk/ | I0-99 |
|  | Germany | Destatis | https://www-genesis.destatis.de/ | I0-99 |
|  | Spain | National Institute of Statistics | <https://www.ine.es> | I10:16, 20:25 46:52, 60:69; 70:73; R96 |
|  |  |  |  |  |
| Incidence data | The Netherlands | Dutch Hospital Data | https://www.dhd.nl/ | I21; I60-69 |
|  | Denmark | Hjerteforeningen | https://hjerteforeningen.dk/ | I21-22; I60-69 |
|  | United Kingdom | British Heart Foundation | https://www.bhf.org.uk/ | I21; I60-69 |
|  | Germany | Destatis | https://www-genesis.destatis.de/ | I21-23; I60-69 |
|  | Spain | National Institute of Statistics | <https://www.ine.es> | I21-23; I6-69 |

Comparison of registry-based incidence rates of total and fatal cardiovascular disease, with those used in the recalibrated LIFE-CVD2. The LIFE-CVD2 predicted risks were obtained by combining regional NCD risk factor levels to the risk algorithms. Note that Dutch, German and Spanish registry-based incidences include also CVD events in patients with prior vascular disease. Only the Spanish registry rates have been corrected for competing risks. NL = The Netherlands, UK = United Kingdom.

## **Supplementary Figure 6**: Aggregated and cohort-specific multiplication factors


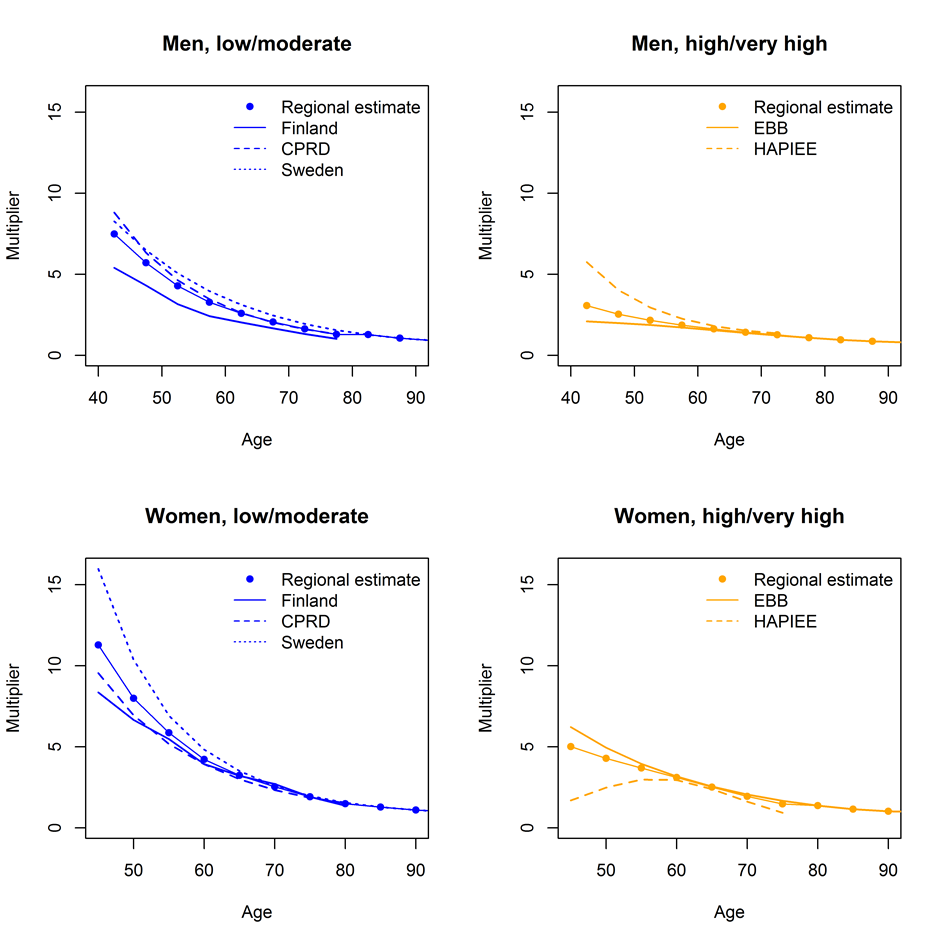


Multiplication factors shown are those used in the previously published recalibration of the SCORE2 and SCORE2-OP models:

Hageman S, Pennells L, Ojeda F, et al. SCORE2 risk prediction algorithms: new models to estimate 10-year risk of cardiovascular disease in Europe. *Eur Heart J*. 2021;42(25):2439-2454. doi:10.1093/eurheartj/ehab309

de Vries TI, Cooney MT, Selmer RM, et al. SCORE2-OP risk prediction algorithms: estimating incident cardiovascular event risk in older persons in four geographical risk regions. *Eur Heart J*. 2021;42(25):2455-2467. doi:10.1093/eurheartj/ehab312

## Supplementary Figure 7: multipliers used to correct for people with prior CVD in national non-CVD mortality rates


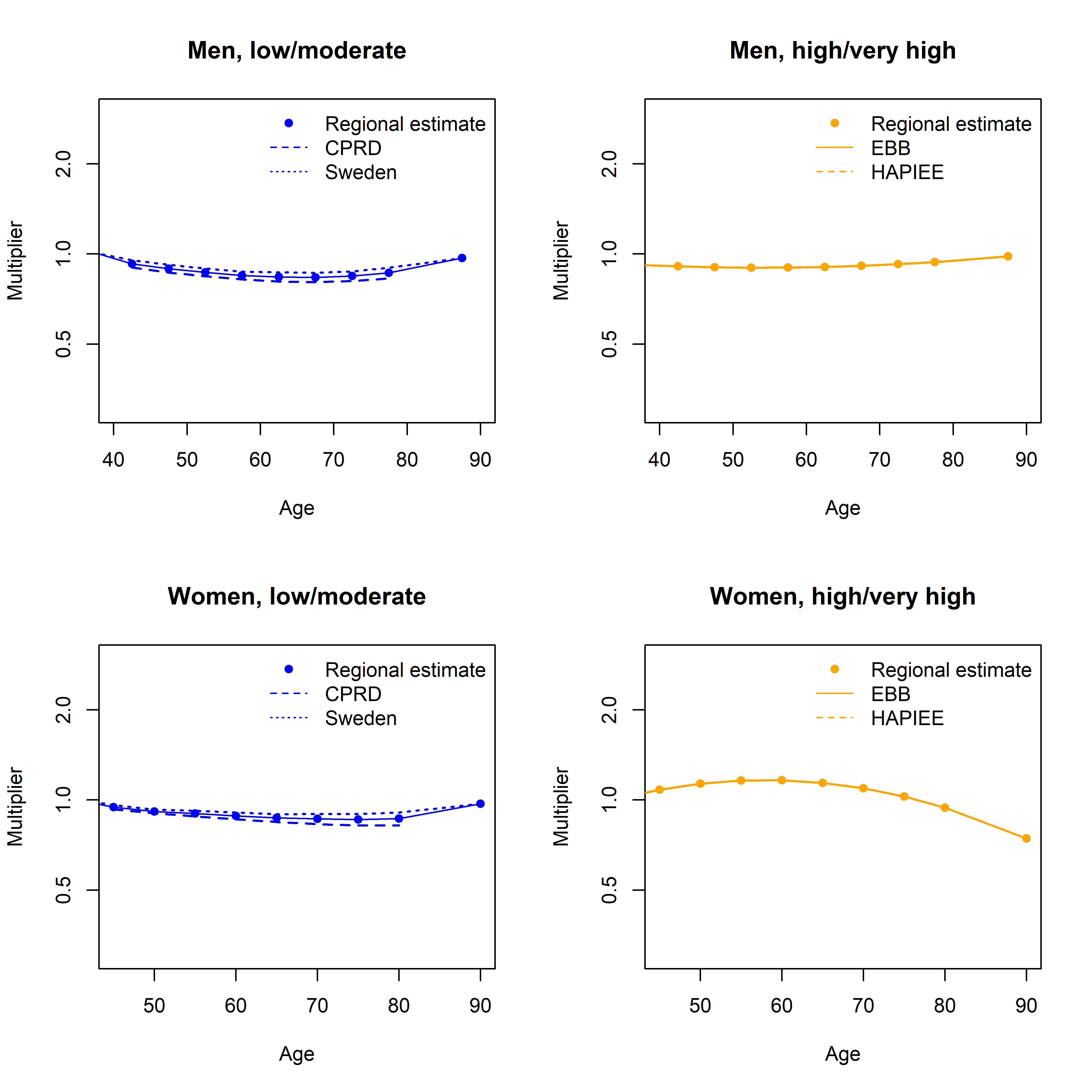


## Supplementary Figure 8: Comparison of 10-year versus 1-year multipliers


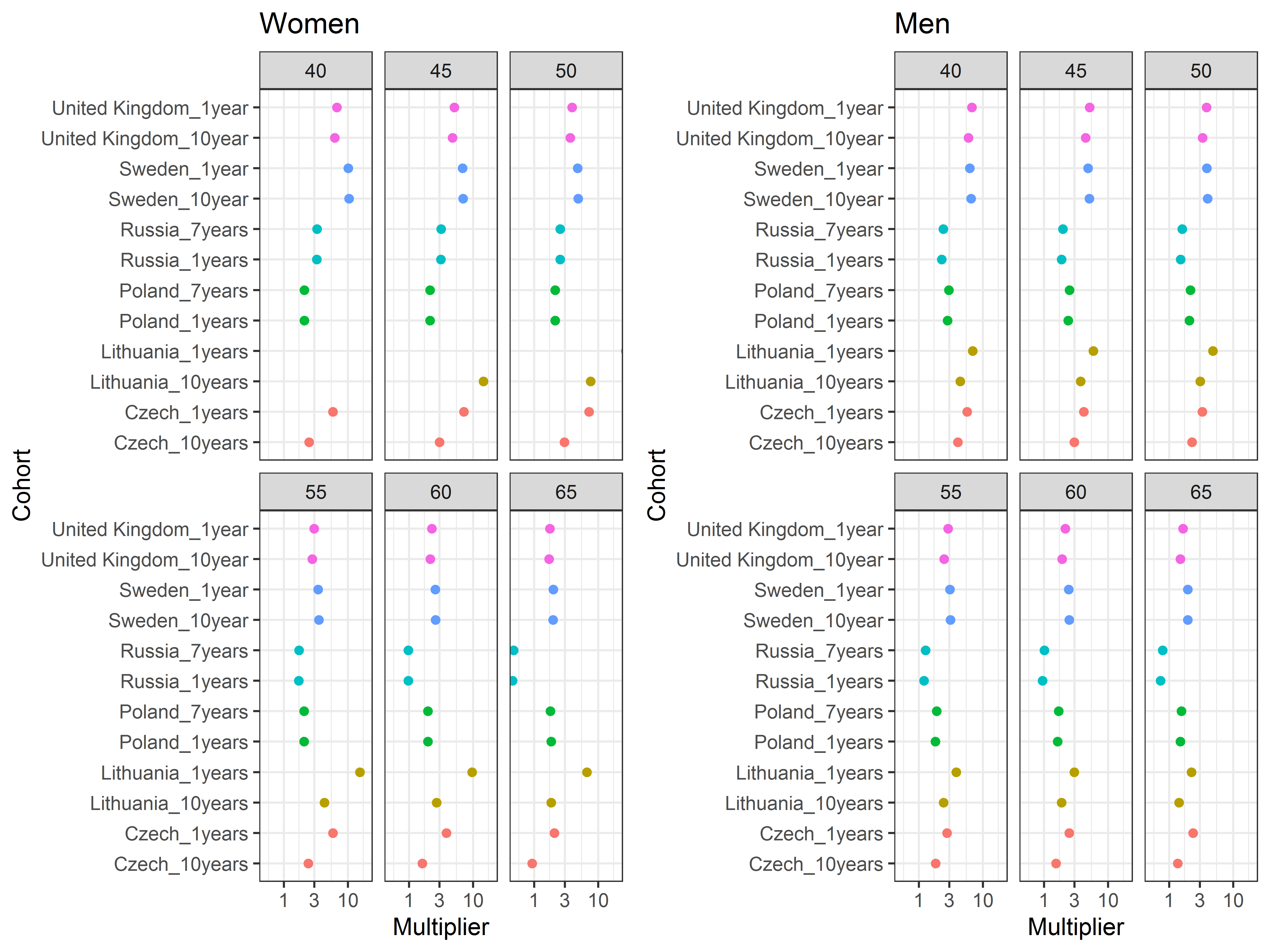


Comparison of the multipliers used for recalibration (10 years if available, else maximum follow-up duration) with multipliers that would be derived at 1-year follow-up.

## Supplementary Figure 9: C-index upon assessing ability of the LIFE-CVD model to discriminate non-CVD mortality in external validation cohorts


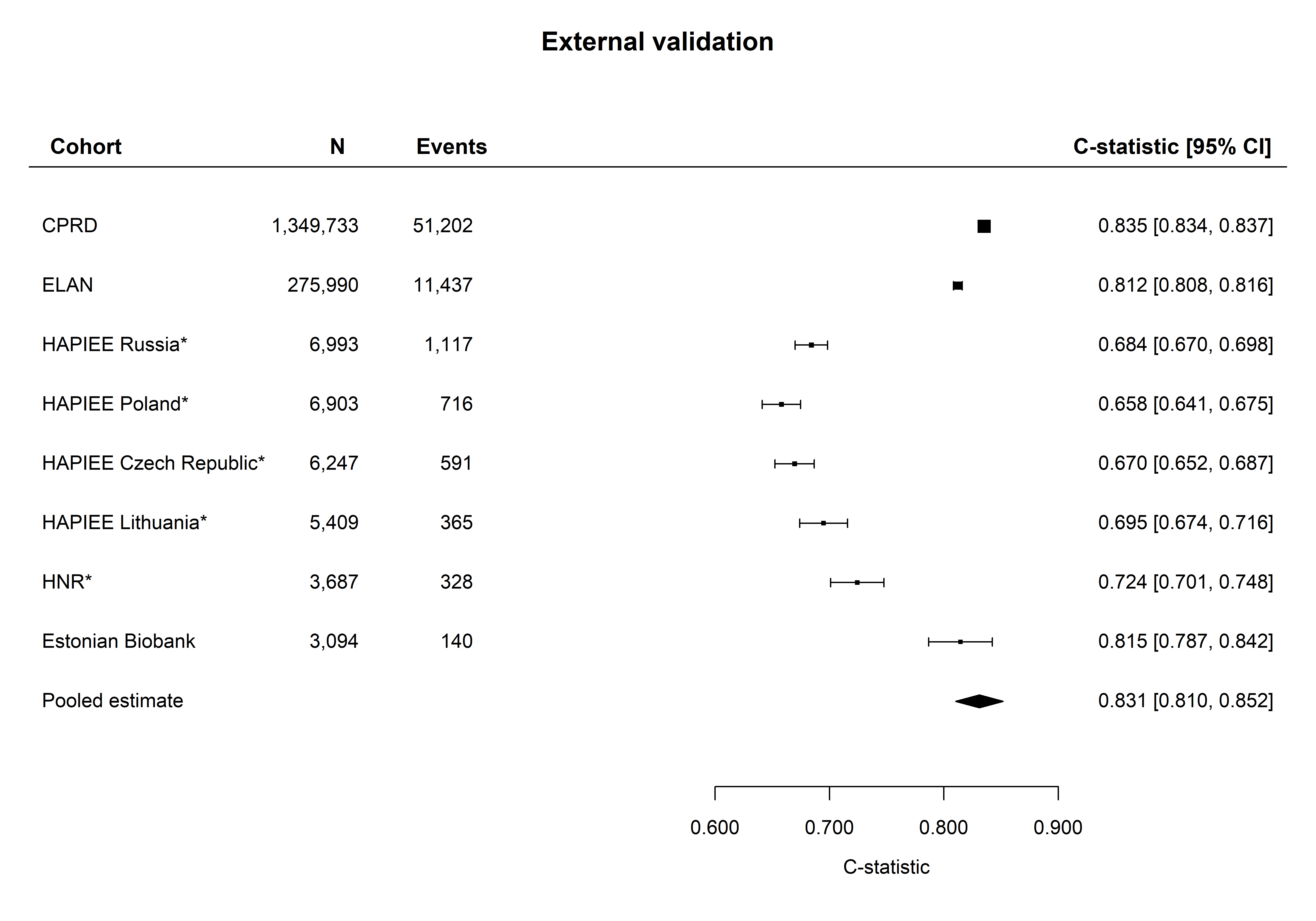


* = as discrimination result may be underestimated because the entire age range of the LIFE-CVD2 model could not be included from this cohort, this result is not included in the pooled estimate. The overall pooled estimate including all studies is 0.824 (95%CI 0.711-0.937)

## Supplementary Figure 10: Calibration for prediction of non-CVD mortality of the recalibrated LIFE-CVD2 model in CPRD (n=1,349,377) and ELAN Vascular (n=275,990)


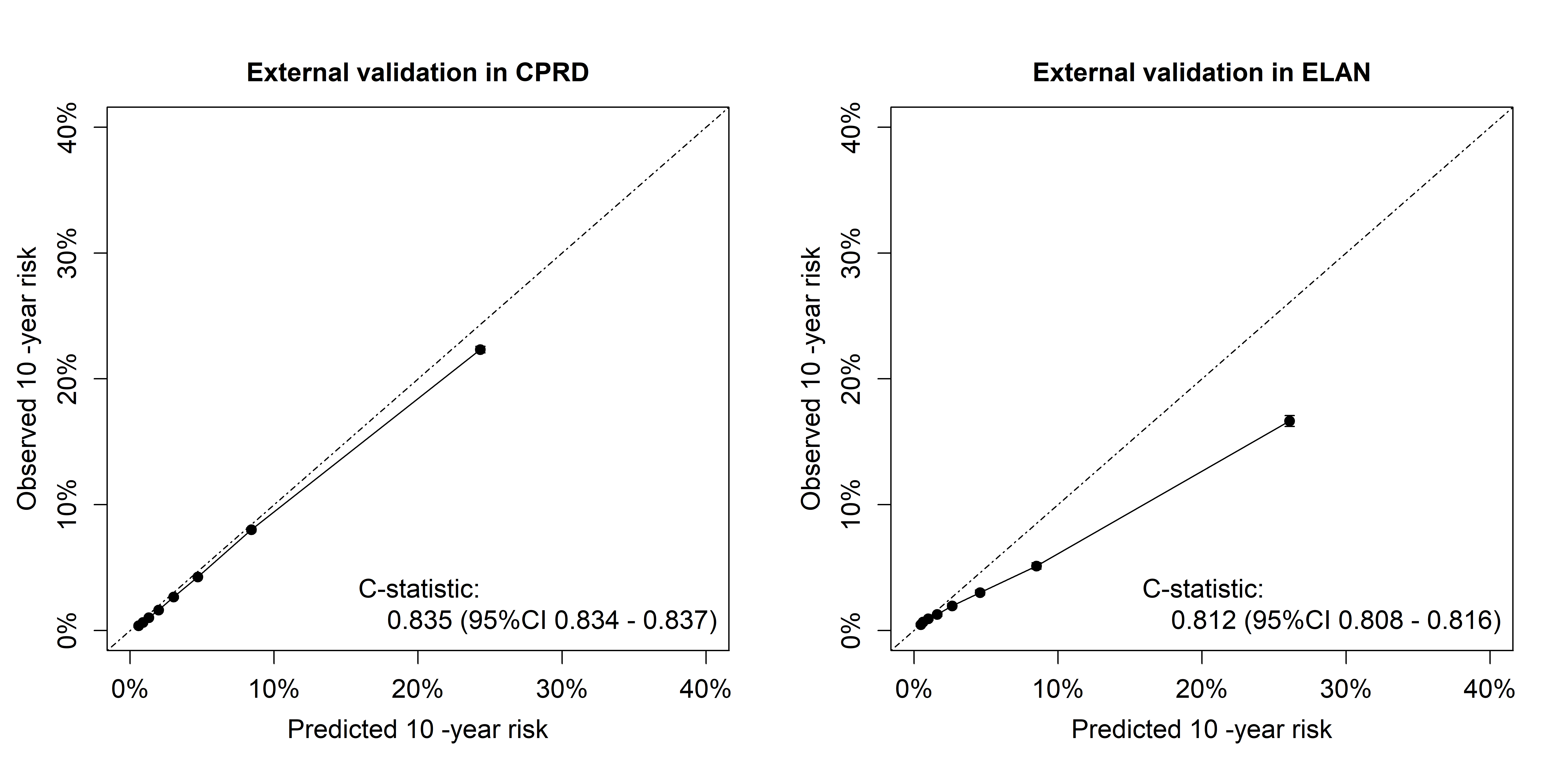


Predicted versus observed non-CVD risks in deciles of predicted risk for the recalibrated LIFE-CVD2 model.

## Supplementary Figure 11: Calibration of all endpoints of LIFE-CVD2 in CPRD (n=1,349,377), also in comparison to SCORE2


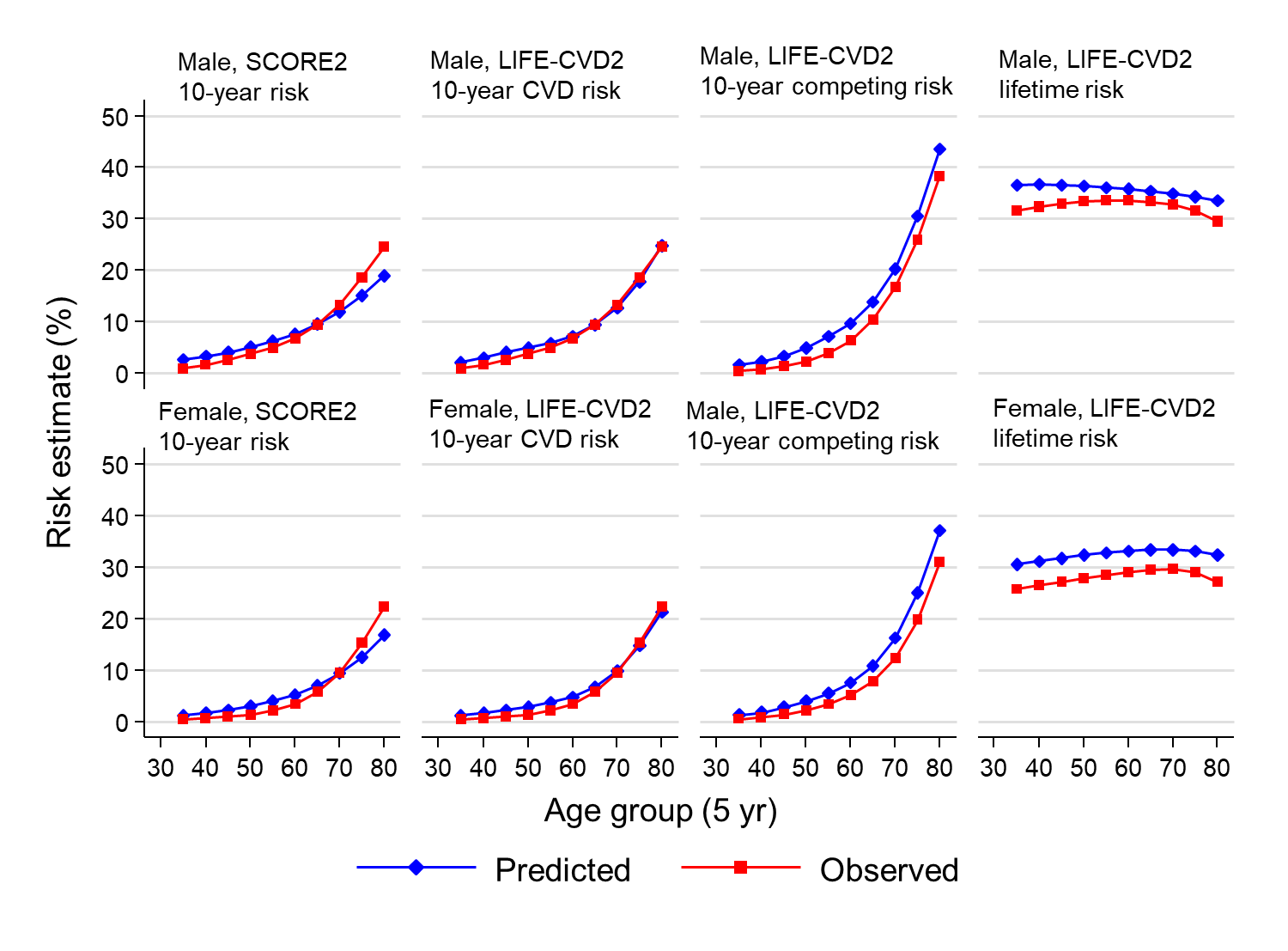


Predicted versus observed risks in every age group for the SCORE2 model, as well as the recalibrated LIFE-CVD2 model. LIFECVD-CVD = predicted 10-year CVD risk, non-CVD = predicted 10-year risk of non-CVD mortality

## Supplementary Figure 12: Predicted gain in CVD-free life expectancy from smoking cessation for an individual with total cholesterol concentration of 5.5 mmol/L, HDL cholesterol of 1.3 mmol/L, and systolic blood pressure of 140 mm Hg, for each region


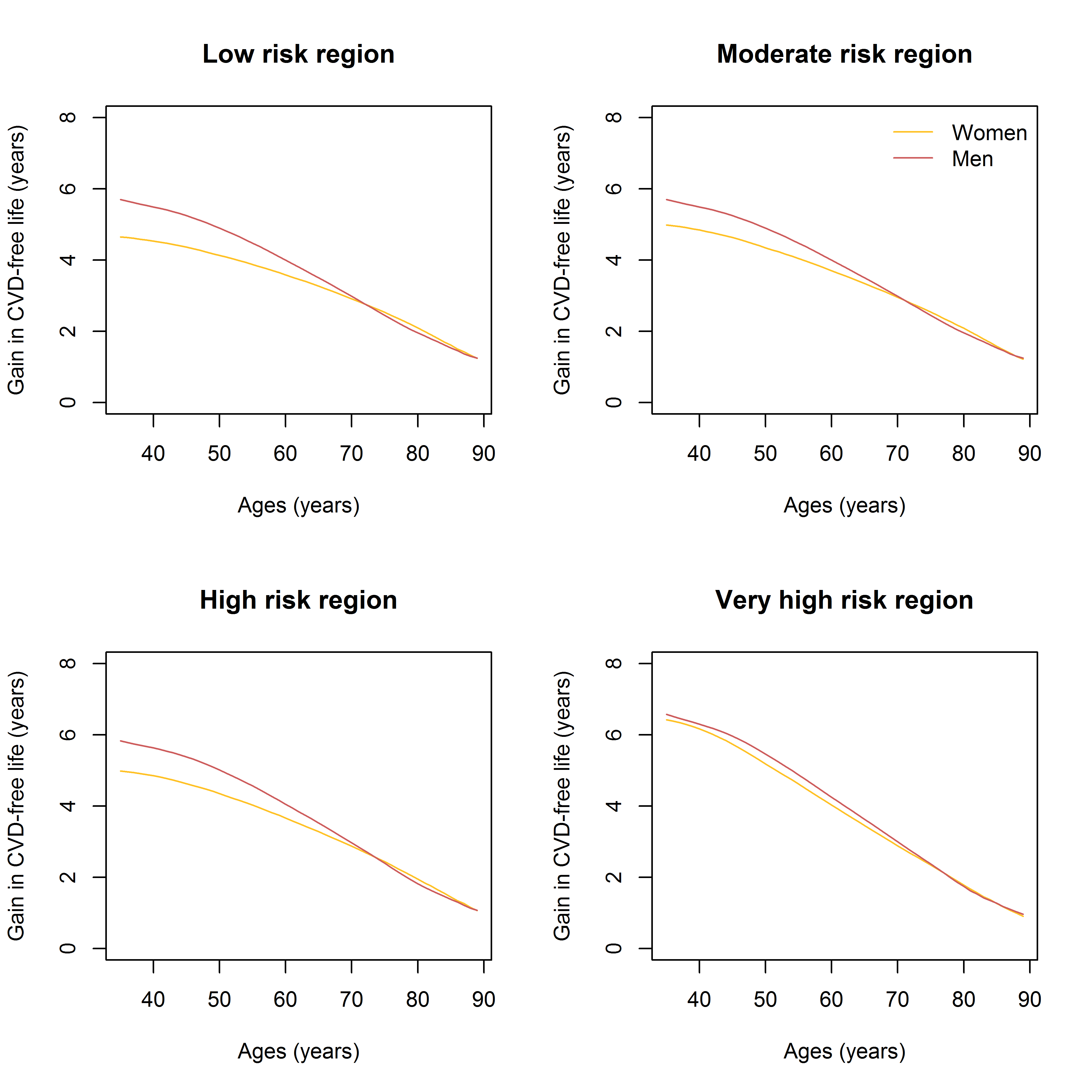


## Supplementary Figure 13: Predicted 10-year CVD risk, lifetime CVD risk, median CVD-free life expectancy, and gain in CVD-free life expectancy from 10 mmHg SBP reduction for a female, non-smoking individual with total cholesterol concentration of 5.5 mmol/L, HDL cholesterol of 1.3 mmol/L, and systolic blood pressure of 140 mm Hg.


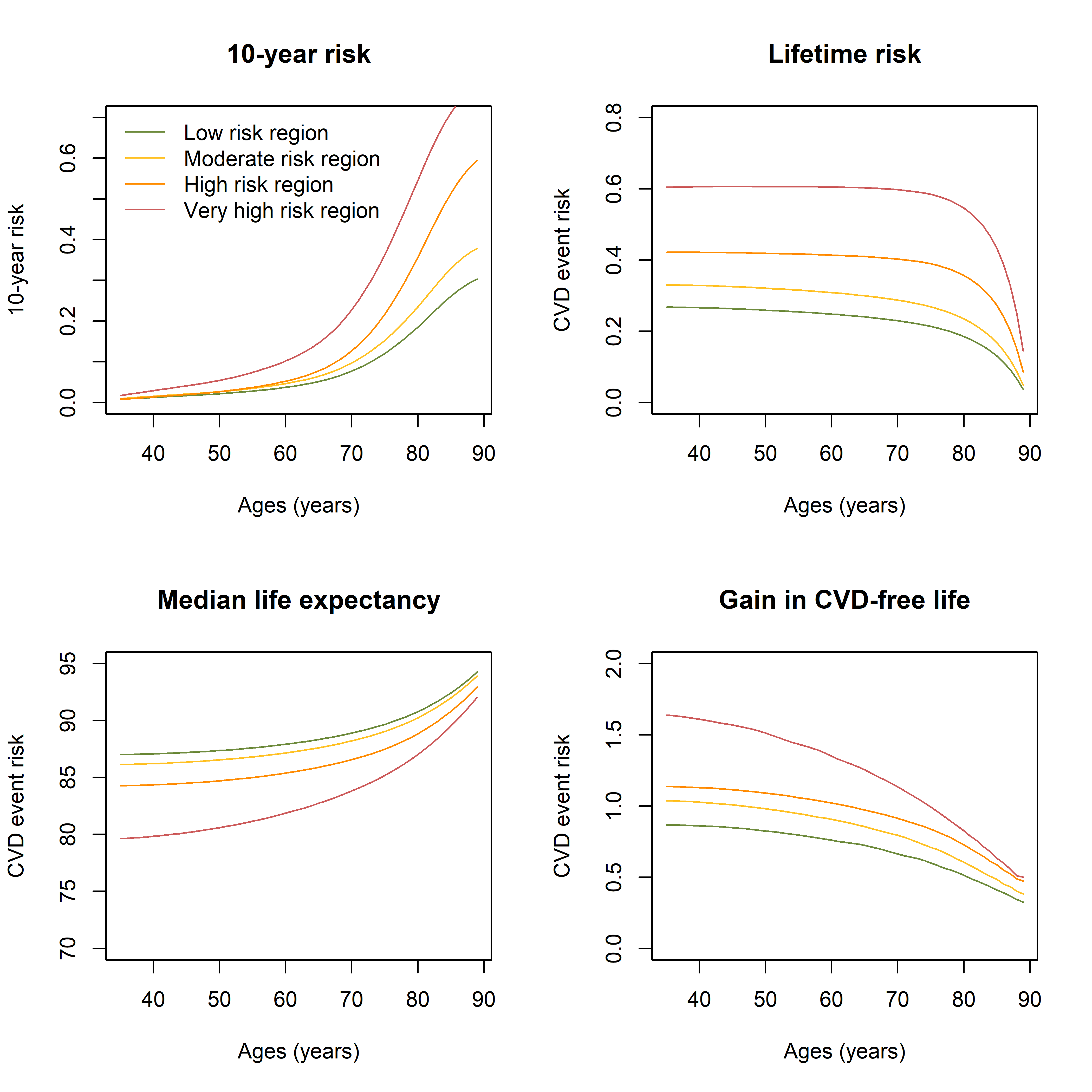


## Supplementary Table 1: Summary of available data in cohorts used for model derivation

| **Cohort** | **Country** | **Median year of baseline** | **Maximum year of follow up** | **Total participants** | **Age at survey (yrs) mean (sd)** | **Male sex, n (%)** | **Diabetes, n (%)** | **Current smoking, n (%)** | **SBP (mmHg) mean (sd)** | **Total cholesterol (mmol/l) mean (sd)** | **HDL-C (mmol/l) mean (sd)** | **Median follow-up (5th & 95th percentiles)** | **CVD, first event** | **Non-CVD death, first event** | **Person-years of follow up, first event** |
| --- | --- | --- | --- | --- | --- | --- | --- | --- | --- | --- | --- | --- | --- | --- | --- |
| **Western Europe** |  |  |  |  |  |  |  |  |  |  |  |  |  |  |  |
| ATENA | Italy | 1995 | 2001 | 4751 | 50 (7) | 0 (0) | 118 (2.5) | 1904 (40) | 134 (21) | 6.1 (1.2) | 1.62 (0.42) | 6.7 (5.2 to 8.1) | 30 | 31 | 31.738 |
| ATTICA | Greek | 2001 | 2012 | 2282 | 51 (11) | 1159 (51) | 199 (8.7) | 968 (42) | 126 (19) | 5.2 (1.0) | 1.24 (0.38) | 5.0 (5.0 to 10.0) | 30 | 20 | 14.948 |
| BRUN | Italy | 1990 | 2010 | 800 | 57 (11) | 391 (49) | 29 (3.6) | 195 (24) | 145 (22) | 5.7 (1.0) | 1.47 (0.37) | 20.2 (4.5 to 20.5) | 143 | 150 | 13.582 |
| BWHHS | UK | 2000 | 2014 | 3266 | 69 (5) | 0 (0) | 141 (4.3) | 392 (12) | 147 (25) | 6.7 (1.2) | 1.67 (0.45) | 12.2 (3.3 to 13.3) | 347 | 496 | 35.929 |
| COPEN | Denmark | 1993 | 2013 | 7733 | 61 (13) | 3272 (42) | 245 (3.2) | 3805 (49) | 140 (22) | 6.3 (1.3) | 1.60 (0.50) | 16.4 (2.3 to 18.4) | 1771 | 1893 | 102.018 |
| DESIR | France | 1995 | 2010 | 4266 | 50 (8) | 2092 (49) | 348 (8.2) | 819 (19) | 132 (16) | 5.8 (1.0) | 1.64 (0.43) | 8.9 (0.0 to 9.3) | 51 | 1 | 32.894 |
| DRECE | Spain | 1991 | 2011 | 1852 | 47 (7) | 885 (48) | 169 (9.1) | 561 (30) | 125 (18) | 5.6 (1.1) | 1.41 (0.37) | 19.3 (12.5 to 19.6) | 38 | 141 | 34.298 |
| EMOFRI | Italy | 1996 | 2002 | 360 | 55 (6) | 176 (49) | 17 (4.7) | 92 (26) | 146 (18) | 5.9 (1.1) | 1.54 (0.42) | 6.8 (6.5 to 7.2) | 8 | 5 | 2.396 |
| EPICNOR | UK | 1996 | 2005 | 20842 | 59 (9) | 9239 (44) | 597 (2.9) | 2422 (12) | 135 (18) | 6.2 (1.2) | 1.42 (0.43) | 9.7 (5.9 to 12.0) | 960 | 0 | 199.266 |
| ESTHER | Germany | 2001 | 2015 | 7962 | 61 (7) | 3381 (42) | 942 (11.8) | 1384 (17) | 139 (19) | 5.7 (1.3) | 1.40 (0.40) | 5.0 (1.5 to 5.9) | 223 | 74 | 36.551 |
| FINNMARK | Norway | 2002 | 2009 | 5373 | 59 (10) | 2395 (45) | 211 (3.9) | 1984 (37) | 140 (23) | 6.1 (1.1) | 1.49 (0.42) | 7.5 (5.0 to 7.5) | 117 | 268 | 38.882 |
| GOTO43 | Sweden | 1993 | 2003 | 775 | 50 (0) | 775 (100) | 16 (2.1) | 239 (31) | 130 (16) | 5.9 (1.0) | 1.31 (0.34) | 11.0 (7.9 to 11.7) | 47 | 21 | 8.334 |
| HCS | UK | 2001 | 2012 | 2707 | 66 (3) | 1356 (50) | 351 (13.0) | 369 (14) | 133 (19) | 6.3 (1.1) | 1.54 (0.42) | 8.9 (5.6 to 11.6) | 62 | 205 | 23.765 |
| HOORN | Netherlands | 1991 | 2005 | 2285 | 61 (7) | 1009 (44) | 219 (9.6) | 720 (32) | 135 (20) | 6.7 (1.2) | 1.34 (0.37) | 8.8 (3.5 to 9.9) | 177 | 149 | 18.954 |
| HUBRO | Norway | 2001 | 2009 | 14143 | 54 (13) | 6132 (43) | 411 (2.9) | 4035 (29) | 135 (20) | 5.8 (1.1) | 1.52 (0.43) | 8.5 (6.0 to 9.5) | 241 | 749 | 122.642 |
| LEADER | UK | 1997 | 2001 | 460 | 68 (9) | 460 (100) | 70 (15.2) | 178 (39) | 150 (22) | 5.6 (0.9) | 1.22 (0.39) | 4.2 (1.0 to 6.8) | 87 | 40 | 1.863 |
| MATISS93 | Italy | 1994 | 2002 | 1218 | 49 (9) | 591 (49) | 58 (4.8) | 326 (27) | 139 (23) | 5.7 (1.1) | 1.30 (0.36) | 8.3 (7.1 to 9.3) | 32 | 16 | 9.971 |
| MIDFAM | UK | 1996 | 2013 | 2162 | 46 (6) | 958 (44) | 26 (1.2) | 536 (25) | 127 (16) | 5.3 (1.0) | 1.41 (0.36) | 17.4 (10.6 to 17.8) | 103 | 109 | 36.100 |
| MONFRI94 | Italy | 1994 | 2002 | 1295 | 49 (8) | 631 (49) | 59 (4.6) | 349 (27) | 137 (20) | 5.8 (1.1) | 1.50 (0.39) | 8.5 (8.0 to 8.8) | 39 | 27 | 10.736 |
| MONICA_KORA2 | Germany | 1990 | 1998 | 3821 | 54 (11) | 1881 (49) | 200 (5.2) | 893 (23) | 135 (19) | 6.3 (1.2) | 1.49 (0.44) | 7.9 (2.1 to 8.4) | 129 | 135 | 27.363 |
| MONICA_KORA3 | Germany | 1995 | 2009 | 3772 | 54 (11) | 1844 (49) | 203 (5.4) | 863 (23) | 135 (20) | 6.1 (1.1) | 1.40 (0.43) | 13.9 (3.7 to 14.7) | 291 | 267 | 46.734 |
| MORGEN | Netherlands | 1995 | 2007 | 16092 | 48 (7) | 7351 (46) | 240 (1.5) | 5978 (37) | 123 (17) | 5.5 (1.0) | 1.36 (0.39) | 10.7 (3.6 to 13.0) | 146 | 437 | 165.082 |
| MOSWEGOT | Sweden | 1990 | 2003 | 3467 | 50 (9) | 1626 (47) | 95 (2.7) | 1022 (29) | 130 (19) | 6.0 (1.2) | 1.45 (0.41) | 13.8 (6.6 to 19.6) | 299 | 165 | 45.613 |
| OPPHED | Norway | 2001 | 2009 | 9485 | 52 (12) | 4283 (45) | 238 (2.5) | 2972 (31) | 135 (22) | 5.8 (1.1) | 1.38 (0.37) | 8.5 (7.0 to 9.5) | 163 | 424 | 80.015 |
| OSLO2 | Norway | 2000 | 2009 | 5109 | 69 (7) | 5109 (100) | 270 (5.3) | 1116 (22) | 147 (21) | 6.1 (1.0) | 1.44 (0.40) | 9.5 (3.0 to 9.5) | 270 | 829 | 43.840 |
| PREVEND | Netherlands | 1998 | 2010 | 5888 | 51 (11) | 2775 (47) | 229 (3.9) | 1971 (33) | 130 (21) | 5.8 (1.1) | 1.34 (0.40) | 10.6 (3.9 to 11.2) | 258 | 297 | 58.081 |
| **Cohort** | **Country** | **Median year of baseline** | **Maximum year of follow up** | **Total participants** | **Age at survey (yrs) mean (sd)** | **Male sex, n (%)** | **Diabetes, n (%)** | **Current smoking, n (%)** | **SBP (mmHg) mean (sd)** | **Total cholesterol (mmol/l) mean (sd)** | **HDL-C (mmol/l) mean (sd)** | **Median follow-up (5th & 95th percentiles)** | **CVD, first event** | **Non-CVD death, first event** | **Person-years of follow up, first event** |
| PROSPER | Scotland/Ireland/Netherland | 1998 | 2002 | 1662 | 75 (3) | 695 (42) | 207 (12.5) | 569 (34) | 157 (21) | 5.7 (0.9) | 1.31 (0.35) | 3.2 (1.2 to 3.9) | 208 | 74 | 5.147 |
| ProspectEPIC | Netherlands | 1995 | 2012 | 15819 | 57 (6) | 0 (0) | 321 (2.0) | 3599 (23) | 133 (20) | 6.1 (1.1) | 1.49 (0.41) | 14.3 (7.6 to 17.2) | 740 | 935 | 218.365 |
| RS_I | Netherlands | 1992 | 2005 | 4923 | 68 (8) | 1874 (38) | 361 (7.3) | 1145 (23) | 139 (22) | 6.7 (1.2) | 1.38 (0.37) | 12.0 (2.9 to 14.2) | 784 | 980 | 52.474 |
| RS_II | Netherlands | 2000 | 2011 | 2206 | 64 (7) | 961 (44) | 216 (9.8) | 527 (24) | 143 (21) | 5.8 (1.0) | 1.39 (0.37) | 10.1 (3.6 to 10.9) | 173 | 188 | 20.657 |
| SHIP | Germany | 1999 | 2011 | 2865 | 56 (12) | 1388 (48) | 370 (12.9) | 767 (27) | 140 (21) | 6.0 (1.2) | 1.46 (0.45) | 10.4 (0.0 to 12.1) | 58 | 0 | 17.872 |
| TROMS | Norway | 2002 | 2009 | 1982 | 51 (12) | 853 (43) | 57 (2.9) | 682 (34) | 135 (21) | 5.8 (1.1) | 1.41 (0.37) | 7.5 (7.5 to 7.5) | 27 | 52 | 14.702 |
| UKB | UK | 2009 | 2020 | 431971 | 56 (8) | 192339 (45) | 18451 (4.3) | 45310 (10) | 138 (19) | 5.8 (1.1) | 1.46 (0.38) | 10.8 (7.8 to 12.4) | 13778 | 15947 | 4.513.415 |
| WHITEII | UK | 1992 | 2004 | 8621 | 50 (6) | 5907 (69) | 145 (1.7) | 1627 (19) | 121 (14) | 6.5 (1.2) | 1.43 (0.41) | 12.2 (3.9 to 13.0) | 323 | 280 | 95.278 |
| WOSCOPS | UK | 1990 | 1995 | 3293 | 55 (6) | 3293 (100) | 35 (1.1) | 1460 (44) | 136 (17) | 7.0 (0.6) | 1.14 (0.25) | 4.8 (2.5 to 6.0) | 300 | 59 | 15.172 |
| ZARAGOZA | Spain | 1994 | 1999 | 4845 | 57 (13) | 2118 (44) | 580 (12.0) | 1004 (21) | 132 (16) | 5.9 (1.0) | 1.42 (0.36) | 5.1 (4.6 to 5.1) | 140 | 5 | 23.961 |
| **SUBTOTAL Western Europe** | | **1995** | **2020** | **610353** | **57 (8)** | **269199 (44)** | **26444 (4.3)** | **92783 (15)** | **136 (19)** | **6.0 (1.1)** | **1.43 (0.39)** | **10.6 (5.0 to 13.1)** | **22593** | **25469** | **6.218.638** |
| **North America** |  |  |  |  |  |  |  |  |  |  |  |  |  |  |  |
| ARIC | USA | 1991 | 2017 | 12006 | 57 (6) | 5365 (45) | 1771 (14.8) | 2926 (24) | 121 (19) | 5.4 (1.0) | 1.29 (0.43) | 25.3 (4.3 to 27.6) | 2798 | 3126 | 250.531 |
| CHS1 | USA | 1989 | 2007 | 3794 | 72 (5) | 1445 (38) | 492 (13.0) | 452 (12) | 136 (21) | 5.5 (1.0) | 1.43 (0.41) | 12.1 (2.0 to 12.9) | 1067 | 791 | 37.254 |
| CHS2 | USA | 1993 | 2005 | 465 | 72 (5) | 173 (37) | 108 (23.2) | 77 (17) | 143 (23) | 5.4 (1.0) | 1.50 (0.41) | 9.1 (1.8 to 9.5) | 108 | 61 | 3.594 |
| HONOL | USA | 1992 | 1999 | 2571 | 78 (4) | 2571 (100) | 651 (25.3) | 197 (8) | 150 (23) | 4.9 (0.9) | 1.34 (0.35) | 6.2 (1.4 to 7.6) | 318 | 456 | 14.575 |
| MESA | USA | 2001 | 2018 | 6805 | 62 (10) | 3207 (47) | 861 (12.7) | 1019 (15) | 126 (21) | 5.0 (0.9) | 1.32 (0.38) | 15.7 (2.6 to 17.1) | 559 | 548 | 89.689 |
| NHANESIII | USA | 1990 | 2013 | 10993 | 57 (15) | 5052 (46) | 1040 (9.5) | 2852 (26) | 132 (21) | 5.5 (1.1) | 1.34 (0.41) | 18.2 (3.1 to 22.4) | 1727 | 2911 | 175.328 |
| NSHS | Canada | 1995 | 2005 | 1402 | 56 (14) | 672 (48) | 74 (5.3) | 389 (28) | 128 (17) | 5.6 (1.1) | 1.28 (0.37) | 9.7 (4.0 to 10.0) | 67 | 138 | 12.666 |
| USPHS2 | USA | 1997 | 2008 | 10724 | 64 (8) | 10724 (100) | 25 (0.2) | 509 (5) | 128 (12) | 5.3 (0.9) | 1.15 (0.38) | 10.9 (4.9 to 11.5) | 645 | 690 | 103.502 |
| WHS | USA | 1994 | 2013 | 28022 | 55 (7) | 0 (0) | 768 (2.7) | 3267 (12) | 127 (12) | 5.5 (1.1) | 1.39 (0.39) | 19.1 (8.8 to 20.0) | 1057 | 94 | 485.254 |
| **SUBTOTAL North America** | | **1995** | **2020** | **76782** | **64 (9)** | **29209 (38)** | **5790 (7.5)** | **11688 (15)** | **132 (17)** | **5.4 (1.0)** | **1.34 (0.40)** | **16.8 (4.1 to 26.1)** | **8346** | **8815** | **1.172.393** |
| **TOTAL** |  | **1995** | **2020** | **687135** | **58 (8)** | **298408 (43)** | **32234 (4.7)** | **104471 (15)** | **135 (19)** | **5.9 (1.1)** | **1.41 (0.39)** | **10.7 (5.0 to 18.5)** | **30939** | **34284** | **7.391.031** |

CVD =cardiovascular disease, HDL = high density lipoprotein, SBP = systolic blood pressure

## Supplementary Table 2: Endpoint definitions

| **Fatal cardiovascular disease– cause specific mortality due to any of the following:** | | |
| --- | --- | --- |
| *Endpoints included* | *ICD10-codes* | *ICD9-codes* |
| Hypertensive disease | I10-16 | 401 – 405 |
| Ischemic heart disease | I20-25 | 410 - 414 |
| Arrhythmias, heart failure | I46-52 | 426 - 429 |
| Cerebrovascular disease | I60-69 | 430 - 438 |
| Atherosclerosis/AAA | I70-73 | 440 - 443 |
| Sudden death and death within 24h of symptom onset | R96.0-96.1 | 798.1 , 798.2 |
|  |  |  |
| Endpoints excluded from the above endpoint: | | |
| Myocarditis, unspecified | I51.4 | 426.7 |
| Subarachnoid haemorrhage | I60 | 429 |
| Subdural haemorrhage | I62 | 430 |
| Cerebral aneurysm | I67.1 | 432.1 |
| Cerebral arteritis | I68.2 | 437.3 |
| Moyamoya | I67.5 | 437.4 |
|  |  |  |
| ***Non cardiovascular mortality – any mortality not included in ‘fatal cardiovascular disease’.*** |  |  |
|  |  |  |
| ***Non-fatal cardiovascular disease*** | | |
| Non-fatal myocardial infarction | I21-I23 | *410* |
| Non-fatal stroke | I60-69 | *430-438* |
|  |  |  |
| *Excluded from the non-fatal stroke endpoint:* | | |
| Subarachnoid hemorrhage | I60 | 429 |
| Subdural hemorrhage | I62 | 430 |
| Cerebral aneurysm | I67.1 | 432.1 |
| Cerebral arteritis | I68.2 | 437.3 |
| Moyamoya | I67.5 | 437.4 |

## Supplementary Table 3: Unrounded model coefficients

|  | **Men, CVD events** | **Men, non-CVD mortality** | **Women, CVD events** | **Women, non-CVD mortality** |
| --- | --- | --- | --- | --- |
| Age (per 5 years) | 0.0977 | -0.0081 | 0.119 | -0.1035 |
| Current smoking | 0.6425 | 0.7987 | 0.8202 | 0.7806 |
| SBP (per 20mmHg) | 0.2894 | 0.0745 | 0.3358 | 0.058 |
| History of diabetes mellitus^+^ | 0.6627 | 0.4738 | 0.8541 | 0.5418 |
| Total cholesterol (per 1 mmol/L) | 0.1521 | -0.0878 | 0.1019 | -0.0461 |
| HDL cholesterol (per 0.5 mmol/L) | -0.2744 | 0.0979 | -0.2457 | -0.0524 |
| Current smoking* age (per 5 year) | -0.063 | -0.0682 | -0.1001 | -0.0154 |
| SBP (per 20mmHg) * age (per 5 year) | -0.0373 | -0.0371 | -0.0509 | -0.0187 |
| History of diabetes mellitus* age (per 5 year) | -0.0911 | -0.088 | -0.1217 | -0.0652 |
| Total cholesterol (per 1 mmol/L) * age (per 5 year) | -0.0291 | -0.0007 | -0.0299 | -0.0174 |
| HDL cholesterol (per 0.5 mmol/L) * age (per 5 year) | 0.0424 | -0.0245 | 0.041 | 0.0143 |

^+^ ^Diabetes mellitus was included in the modelling as diabetes patients are included in the recalibration data. For use in clinical practice this coefficient should be ignored.^

## Supplementary Table 4: Baseline survival

| **Age (years)** | **CVD, men** | **CVD, women** | **non-CVD mortality, men** | **non-CVD mortality, women** |
| --- | --- | --- | --- | --- |
| 35 | 0.99905 | 0.99975 | 0.99921 | 0.99964 |
| 36 | 0.99912 | 0.99970 | 0.99923 | 0.99960 |
| 37 | 0.99915 | 0.99964 | 0.99922 | 0.99955 |
| 38 | 0.99912 | 0.99957 | 0.99920 | 0.99950 |
| 39 | 0.99906 | 0.99951 | 0.99916 | 0.99945 |
| 40 | 0.99897 | 0.99944 | 0.99911 | 0.99939 |
| 41 | 0.99886 | 0.99936 | 0.99906 | 0.99934 |
| 42 | 0.99873 | 0.99929 | 0.99899 | 0.99928 |
| 43 | 0.99859 | 0.99921 | 0.99892 | 0.99921 |
| 44 | 0.99837 | 0.99912 | 0.99884 | 0.99915 |
| 45 | 0.99810 | 0.99902 | 0.99874 | 0.99909 |
| 46 | 0.99782 | 0.99893 | 0.99862 | 0.99902 |
| 47 | 0.99757 | 0.99885 | 0.99850 | 0.99894 |
| 48 | 0.99739 | 0.99879 | 0.99835 | 0.99885 |
| 49 | 0.99724 | 0.99875 | 0.99818 | 0.99873 |
| 50 | 0.99710 | 0.99871 | 0.99799 | 0.99859 |
| 51 | 0.99697 | 0.99867 | 0.99777 | 0.99843 |
| 52 | 0.99683 | 0.99862 | 0.99754 | 0.99827 |
| 53 | 0.99666 | 0.99856 | 0.99731 | 0.99810 |
| 54 | 0.99647 | 0.99847 | 0.99705 | 0.99792 |
| 55 | 0.99626 | 0.99836 | 0.99678 | 0.99772 |
| 56 | 0.99604 | 0.99824 | 0.99649 | 0.99752 |
| 57 | 0.99585 | 0.99812 | 0.99617 | 0.99730 |
| 58 | 0.99568 | 0.99801 | 0.99581 | 0.99708 |
| 59 | 0.99554 | 0.99790 | 0.99539 | 0.99686 |
| 60 | 0.99541 | 0.99778 | 0.99492 | 0.99662 |
| 61 | 0.99527 | 0.99766 | 0.99442 | 0.99637 |
| 62 | 0.99511 | 0.99752 | 0.99390 | 0.99609 |
| 63 | 0.99488 | 0.99737 | 0.99337 | 0.99576 |
| 64 | 0.99455 | 0.99720 | 0.99284 | 0.99537 |
| 65 | 0.99415 | 0.99701 | 0.99227 | 0.99492 |
| 66 | 0.99370 | 0.99680 | 0.99166 | 0.99443 |
| 67 | 0.99323 | 0.99656 | 0.99097 | 0.99391 |
| 68 | 0.99278 | 0.99631 | 0.99019 | 0.99335 |
| 69 | 0.99232 | 0.99602 | 0.98929 | 0.99277 |
| 70 | 0.99184 | 0.99570 | 0.98826 | 0.99213 |
| 71 | 0.99131 | 0.99533 | 0.98712 | 0.99142 |
| 72 | 0.99072 | 0.99490 | 0.98586 | 0.99062 |
| 73 | 0.99005 | 0.99438 | 0.98450 | 0.98970 |
| 74 | 0.98926 | 0.99373 | 0.98299 | 0.98865 |
| 75 | 0.98836 | 0.99294 | 0.98130 | 0.98742 |
| 76 | 0.98734 | 0.99202 | 0.97939 | 0.98603 |
| 77 | 0.98621 | 0.99100 | 0.97722 | 0.98445 |
| 78 | 0.98497 | 0.98988 | 0.97473 | 0.98264 |
| 79 | 0.98359 | 0.98857 | 0.97170 | 0.98044 |
| 80 | 0.98201 | 0.98705 | 0.96816 | 0.97785 |
| 81 | 0.98017 | 0.98531 | 0.96414 | 0.97492 |
| 82 | 0.97801 | 0.98335 | 0.95971 | 0.97170 |
| 83 | 0.97534 | 0.98112 | 0.95484 | 0.96822 |
| 84 | 0.97152 | 0.97839 | 0.94918 | 0.96432 |
| 85 | 0.96682 | 0.97520 | 0.94274 | 0.95992 |
| 86 | 0.96166 | 0.97161 | 0.93559 | 0.95494 |
| 87 | 0.95646 | 0.96768 | 0.92781 | 0.94933 |
| 88 | 0.95143 | 0.96338 | 0.91932 | 0.94292 |
| 89 | 0.94587 | 0.95830 | 0.90943 | 0.93512 |
| 90 | 0.94009 | 0.95259 | 0.89842 | 0.92606 |
| 91 | 0.93461 | 0.94648 | 0.88671 | 0.91596 |
| 92 | 0.92996 | 0.94021 | 0.87473 | 0.90507 |
| 93 | 0.92647 | 0.93397 | 0.86279 | 0.89349 |
| 94 | 0.92337 | 0.92755 | 0.85053 | 0.88056 |
| 95 | 0.92060 | 0.92089 | 0.83786 | 0.86630 |
| 96 | 0.91827 | 0.91401 | 0.82481 | 0.85085 |
| 97 | 0.91651 | 0.90691 | 0.81139 | 0.83435 |
| 98 | 0.91543 | 0.89962 | 0.79762 | 0.81694 |
| 99 | 0.91515 | 0.89215 | 0.78351 | 0.79878 |
| 100 | 0.91579 | 0.88449 | 0.76909 | 0.77999 |

## Supplementary Table 5: Region-specific recalibration scales for calculation of the 1-year CVD event and non-CVD mortality risks

|  | **Male** | | **Female** | |
| --- | --- | --- | --- | --- |
|  | **Scale 1** | **Scale 2** | **Scale 1** | **Scale 2** |
| *CVD events* |  |  |  |  |
| Low risk region | -1.1835 | 0.8139 | -1.4978 | 0.7438 |
| Moderate risk region | -0.8411 | 0.8303 | -1.1887 | 0.759 |
| High risk region | -0.2823 | 0.9427 | -0.2276 | 0.9132 |
| Very high risk region | -0.0096 | 0.8783 | 0.2217 | 0.8675 |
| *Non-CVD mortality* |  |  |  |  |
| Low risk region | 0.4309 | 1.1526 | 1.2916 | 1.2578 |
| Moderate risk region | 0.2261 | 1.1082 | 1.2425 | 1.2582 |
| High risk region | 0.218 | 1.0521 | 0.5902 | 1.0991 |
| Very high risk region | -0.4217 | 0.8938 | -0.3845 | 0.8872 |

Rescaling factors for the LIFE-CVD model to scale individual predicted risks within the life table to the target population, based on recent nationally representative estimates of incident cardiovascular disease and risk factor levels.

## Supplementary Table 6: Baseline characteristics of the external validation populations

| **Geographic region \ Cohort** | **Country** | **Median year of baseline** | **Total participants** | **Age at survey (yrs)** | **Age range (yrs)** |  | **Male sex,**  **n (%)** | **Current smoking,**  **n (%)** | **SBP (mmHg)** | **Total cholesterol (mmol/l)** | **HDL-C (mmol/l)** | **Median follow-up (IQR)** | **CVD, first event** | **Non-CVD death, first event** |
| --- | --- | --- | --- | --- | --- | --- | --- | --- | --- | --- | --- | --- | --- | --- |
| CPRD | United Kingdom | 2006 | 1349377 | 54 ± 13 | 35-85 |  | 648209 (48%) | 595637 (44%) | 132 ± 17 | 5.5 (4.7-6.2) | 1.4 (1.2-1.7) | 7.5 (5.0-10.3) | 44004 | 51202 |
| ELAN | The Netherlands | 2010 | 275990 | 48 ± 13 | 35-99 |  | 130955 (47%) | 84642 (31%) | 133 ± 21 | 5.3 (4.6-6.0) | 1.3 (1.1-1.6) | 9.8 (4.8-13.1) | 14342 | 11437 |
| HNR | Germany | 2001 | 3687 | 59 ± 6 | 45-75 |  | 1677 (45%) | 851 (23%) | 130 ± 20 | 6.0 (5.3-6.9) | 1.5 (1.2-1.8) | 14.0 (10.5-15.6) | 240 | 328 |
| Est BB | Estonia | 2008 | 3094 | 48 ± 10 | 35-84 |  | 1,017 (33%) | 764 (13%) | 123 ± 14 | 5.9 (5.2-6.7) | 1.3 (1.1-1.6) | 13.8 (12.7-14.1) | 285 | 140 |
| HAPIEE | Poland | 2003 | 6903 | 56 ± 7 | 45-69 |  | 3232 (47%) | 2367 (34%) | 137 ± 21 | 5.8 (5.1-6.5) | 1.2 (1.4-1.7) | 6.3 (6.0-6.9) | 369 | 716 |
| HAPIEE | Russia | 2003 | 6993 | 57 ± 7 | 45-69 |  | 3125 (45%) | 2079 (30%) | 141 ± 24 | 6.2 (5.4-7.0) | 1.5 (1.3-1.7) | 9.2 (6.7-10.4) | 732 | 1117 |
| HAPIEE | Czech Republic | 2003 | 6247 | 57 ± 7 | 45-69 |  | 2793 (45%) | 1755 (28%) | 138 ± 20 | 5.0 (4.5-5.6) | 1.4 (1.1-1.6) | 15.0 (13.8-15.8) | 808 | 591 |
| HAPIEE | Lithuania | 2006 | 5409 | 60 ± 8 | 45-69 |  | 2471 (46%) | 1125 (21%) | 139 ± 22 | 5.9 (5.2-6.6) | 1.5 (1.2-1.7) | 8.3 (7.8-9.0) | 611 | 365 |

n (%), mean ± SD, or median (interquartile range; IQR). SBP = systolic blood pressure, HDL-C = high density lipoprotein cholesterol, CVD = cardiovascular disease.

## Supplementary Methods

### Recalibration

The interlinked stages of recalibration are summarised in **Supplementary Methods Figure 1**. The LIFE-CVD2 model was derived in individual patient data from the UK Biobank and ERFC using Cox regression models separately for both sexes (**Box 1**); Four risk regions in Europe were similar to those defined by SCORE2.^2^ The SCORE2 investigators have defined these risk regions according to the age-standardised country-specific cardiovascular mortality rates. For each region, annual age and sex-specific mortality rates were obtained from the WHO and converted to risk estimates, for both CVD mortality and the risk of non-CVD death (**Box 2**); In order to translate 1-year mortality to 1-year risk of fatal and non-fatal CVD, the SCORE2 region- age- and sex-specific multiplication factors were applied to the 1-year CVD mortality risks.; Region, sex and age-specific predicted 1-year risks were then estimated using the un-calibrated LIFE-CVD2 model with region, sex and age-specific risk factors from the Non-Communicable Disease Risk Factor Collaboration (NCD-RisC) (**Box 5**). The region and sex and age-specific predicted risks (from **Box 5**) were compared to expected risks (from **Box 4**) and rescaling factors were estimated to recalibrate the models for each region and sex (**Box 6**). Finally, the rescaling factors are applied with the original un-calibrated model to give new, recalibrated risk predictions in new individuals (**Box 7**).

### Box 1: Model coefficients

The LIFE-CVD2 was derived sex-specific, and for each sex the model consists of two complementary Cox proportional hazards functions for cardiovascular events and non-CVD mortality respectively. These functions use age as the time axis (i.e. left truncation).

### Box 2: Estimation of 10-year competing risk adjusted mortality for each risk region

WHO cause-specific mortality rates were supplied by country and coded in ICD-9 or ICD-10. Rates included all mortality which was included in the original SCORE endpoint. Non-CVD mortality was defined as all mortality not included in the SCORE endpoint. Region-level estimates were obtained by taking the age- and sex- specific median of all country-specific estimates of CVD mortality rates from the relevant region.

In the SCORE2 project, for every age-group, WHO rates representative of the midpoint of the 10-year interval ahead were used - i.e. for the 40 to 44 year age-group the rates for 45 to 49 years was used. **As currently 1-year rather than 10-year intervals are used for recalibration, this is done differently in comparison to the SCORE2 project. Instead, the 40-44 year age-group is recalibrated based on rates observed in the 40-44 year age group**. WHO rates of both the fatal cardiovascular outcome and the competing outcome non-CVD mortality were converted to 1–year mortality risks ($r)$ using the following formula:

$$r=1- e^{(-fatal rate)}$$

As the WHO rates cover 1-year intervals already, no extrapolation is required to 10-year risks as is done in the SCORE2 recalibration procedure.

### Box 3: Use of Multipliers to convert mortality to incidence estimates in each risk region

To convert mortality estimates to incidence estimates, age- and sex-specific multiplication factors were defined as:

$$\frac{{Cumulative incidence total CV events}_{without prior CVD}}{{Cumulative incidence fatal CV events}_{entire population}}$$

These allowed the population level mortality statistics, which are calculated among the whole population, regardless of prior disease status, to be converted into first event incidence estimates, representative of the target primary prevention population (those without prior CVD). To be as consistent as possible to the SCORE2 methodology, no new multipliers were derived for the CVD endpoint. Instead, SCORE2 multipliers were applied to these 1-year CVD mortality rates. The validity of this methodology was further assessed in additional analyses (**Supplementary Figure 5**). As for SCORE2 there were no multipliers available to correct for the competing endpoint of non-CVD mortality, non-CVD mortality multipliers were derived using similar methods. These were defined the following:

$$\frac{{Cumulative incidence non-CVD mortality}_{without prior CVD}}{{Cumulative incidencenon-CVD mortality}_{entire population}}$$

Multiplication factors were assumed to be stable within each region and over time which was additionally verified in several analyses in the SCORE2 project (SCORE2 Supplementary Figure 3-5).^2^

### Box 6 Relate expected to predicted risks to calculate rescaling factors for model recalibration

Recalibration of the core LIFE-CVD2 models was completed separately for each target region and sex using the previously published general process described in **Supplementary Methods Figure 2**. This involved the use of country-sex-specific mean risk factor levels (from NCDRisc) and region-sex-specific estimates of expected cumulative 1-year risk, estimated as described above and in **Boxes 2** and **3**. We used the recalibrated 1-year risk models to estimate 1-year predicted risk of each endpoint for each of the age groups using the mean risk factor values as described in **Box 5**. Having completed this process for each age group, as shown in **Supplementary Methods Figure 2** we then regressed transformed expected 1-year risk across age groups on that predicted by the core LIFE-CVD models to derive recalibration factors (the intercept and slope of the resulting regression line, **Supplementary Table 1**). The LIFE-CVD2 risk models, rescaled using the recalibration factors were then used to estimate appropriate risks for each potential risk factor combination, for a new individual or for formation of the example risk charts.

### Missing data

Because complete case analysis may lead to loss of statistical power and possible bias^1^, values of predictors were imputed by single regression imputation with predictive mean matching for all cohort data.

As the CPRD and ELAN Vascular consist of routine care data, missing data was much more frequent and missingness was more likely to correlate with cardiovascular disease risk. Therefore, multiple imputation was performed for the external validation in these cohorts with fully conditional specification using 5 imputed datasets.

### Predicting treatment effects from risk factor treatment

It has previously been shown that risk estimations can be combined with relative treatment effects from trials to calculate absolute individualized treatment effects.^3,4^ To show the potential use of using LIFE-CVD2 in daily clinical practice, we included an example on the individual absolute benefit of blood pressure lowering (**Figure 4**). To estimate the effect of blood pressure lowering on CVD, average relative treatment effects were added to the risk function predicting CVD events, using a hazard ratio (HR) of 0.80 per 10 mmHg SBP reduction taken from a large meta-analysis for blood pressure lowering, and estimating the benefit from a 10 mmHg blood pressure lowering for a patient example. To calculate the effect of lowering SBP until the guideline recommended target of <140 mm Hg, a target SBP value of 135 mm Hg was modelled. For lipid lowering, an HR of 0.78 per 1 mmol/L LDL reduction was used and the treatment benefit of a 1.5 mmol/L lowering was likewise estimated for a patient example. For both treatment effects, it was assumed that the HR can be applied across the entire age range. Indeed, no evidence for heterogeneity of these treatment effects across different age ranges has been found.

Treatment benefit was calculated for the respective risk factor treatment by combining the HR with the individualized estimated CVD event risks as used in the lifetable (here an example shown for a HR per 10 mmHg SBP reduction):

$${Risk}_{with treatment}=1-{{(1-risk}_{original})}^{\exp\left( \log\left( HR \right)\times(\frac{SBP reduction}{10}) \right)}$$

Treatment effects are calculated in the lifetable for every 1-year separately, thereby taking into account the probability of having a CVD event or non-CVD fatal event before the moment of interest. For smoking cessation both the CVD event curve as well as the non-CVD mortality risk is decreased in the estimation of treatment effects.

Treatment benefit for individual patients is defined as the gain in life years with the initiated treatment:

$Gain in CVDfree life years=median CVDfree life expectancy-treated median CVDfree life expectancy$

### Calculating treatment effects when the life expectancy exceeds 100 years

Treatment benefit for each risk factor treatment is estimated as the difference between on- and off-treatment median CVD-free life expectancy. In the very rare case in which someone’s life expectancy exceeds the model’s maximum age, this approach cannot be used as the cumulative survival curve does not drop below 50%. Previously, we proposed using the difference in area under the curve (AUC) in such cases as a possible solution. However, the AUC-method gives underestimation of true lifetime treatment benefit (as the complete survival would needs to be modelled up to 0% survival probability to give a good estimate based on the AUC). As a more suitable alternative, we here propose a new method of using the last observed cumulative survival. This means that in the case the on-treatment CVD-free cumulative survival exceeds 50% at the maximum age (i.e. 100 years), lifetime treatment benefit is defined as the difference between the maximum age and the age with the corresponding predicted percentage off-treatment cumulative survival. For example, should the predicted survival at the end of the lifetable be 54%, then the median survival can’t be read from the lifetable. Instead, the age at which the survival of 54% rather than 50% is compared on- and off- treatment. With the extended age range due to the extrapolation of the baseline hazard though, this is seldom required, but more accurate in those theoretical cases in which it is necessary.

## **Supplementary Methods, Figure 1**: Model recalibration process


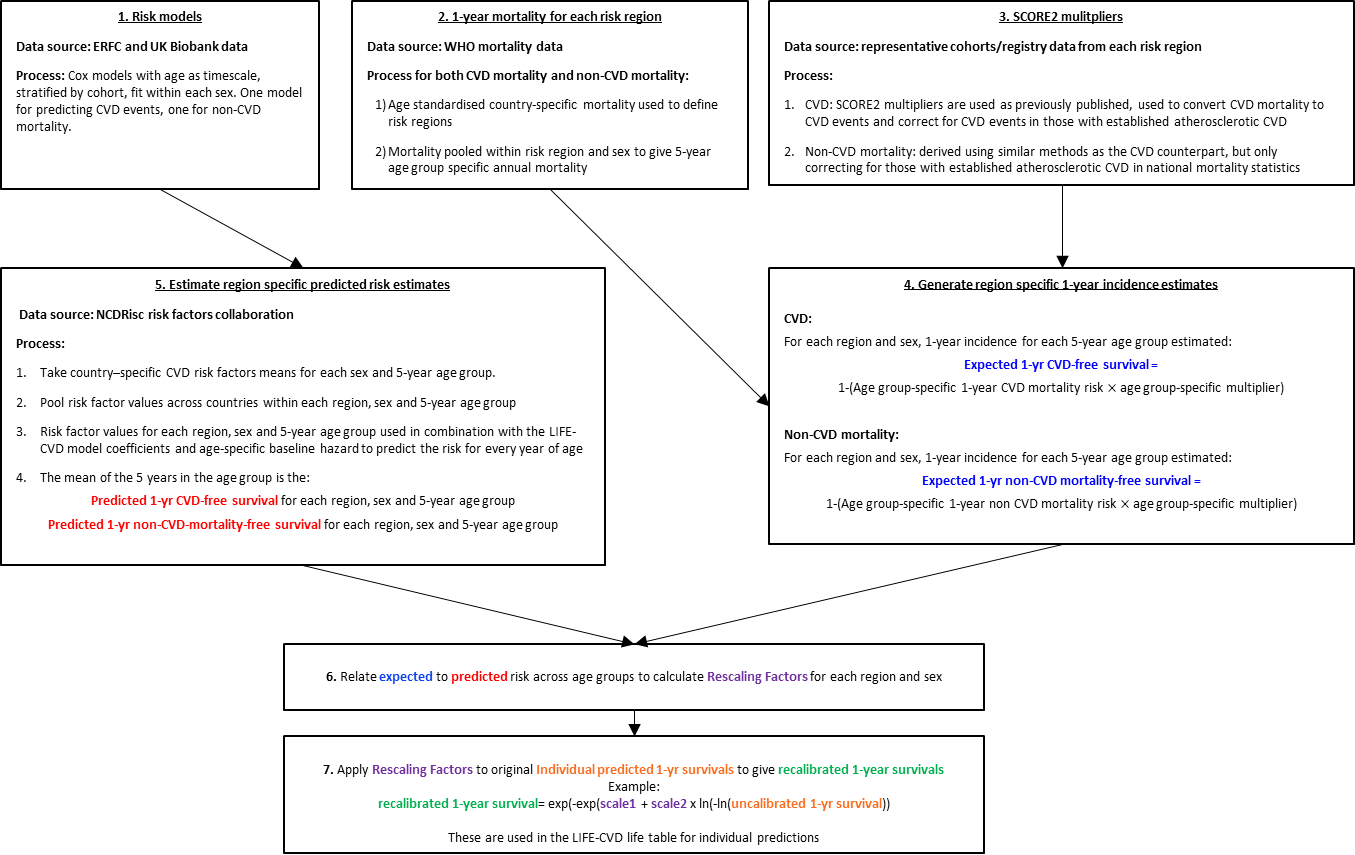


## **Supplementary Methods, Figure 2: Methods used for recalibration of risk scores**


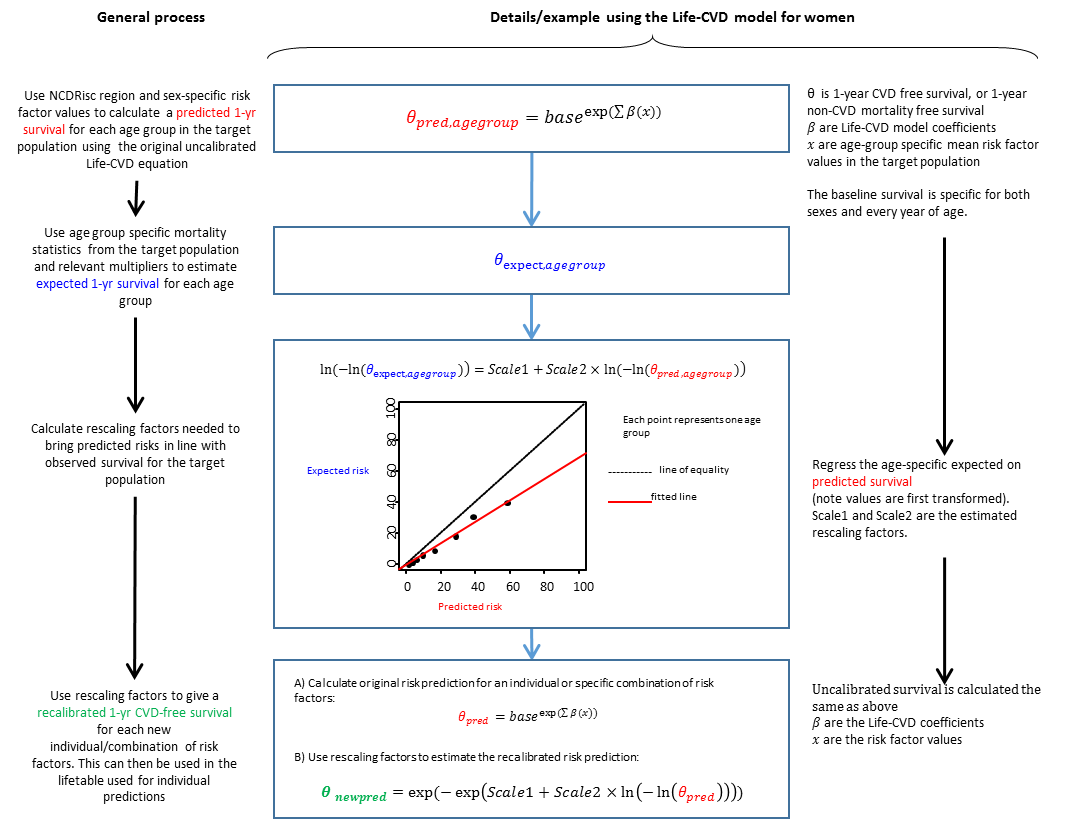

Supplement: zwae174_Supplementary_Data [file zwae174_supplementary_data.zip › Supplement_lifeCVD2_20240213.docx]
